# RNA G‐Quadruplex RIBOTAC‐Mediated Targeted Degradation of lncRNA TERRA

**DOI:** 10.1002/advs.202512715

**Published:** 2025-10-06

**Authors:** Elias Khaskia, Dipak Dahatonde, Raphael I. Benhamou

**Affiliations:** ^1^ The Institute for Drug Research of the School of Pharmacy Faculty of Medicine The Hebrew University of Jerusalem Jerusalem 9112002 Israel

**Keywords:** alternative lengthening of telomeres, G‐quadruplex, lncRNA, RIBOTAC, small molecules, targeted degradation

## Abstract

Long noncoding RNAs (lncRNAs) regulate gene expression and play crucial roles in development and disease, including cancer. One important but still poorly understood lncRNA is TERRA (telomeric repeat‐containing RNA), transcribed from telomeres and essential for telomere maintenance, genome stability, and cellular aging. TERRA adopts two structural features,R‐loops and G‐quadruplexes (G4s), that drive its biological activity. Its dysregulation is linked to telomere dysfunction and is prominent in Alternative Lengthening of Telomeres (ALT) cancers, where TERRA is highly upregulated and promotes telomere recombination. Here, first‐in‐class small molecules targeting TERRA using RIBOTAC (Ribonuclease‐Targeting Chimera) technology is developed. These compounds selectively bind TERRA's G4 structures and recruit RNase L, inducing its degradation while sparing DNA G4s and other G4 RNAs. This selectivity results from ternary complex formation with RNase L and the repetitive G4 motifs enriched in TERRA. TERRA‐RIBOTACs in HeLa and U2OS cells, the latter representing an ALT cancer model, are evaluated. TERRA degradation, particularly at 7p, 13q, 15q, and 20q loci, impairs telomere function and reduces colony formation. Manipulating lncRNAs like TERRA with chemical tools opens new avenues for drug development and deepens the understanding of RNA‐based regulation in cancer biology.

## Graphical abstract

This study introduces a selective RNA degradation strategy targeting long noncoding RNA (lncRNA) TERRA, upregulated in ALT (alternative lengthening of telomeres) cancer cells. Using ribonuclease‐targeting chimeras (RIBOTACs), RNase L is recruited to catalytically degrade TERRA, uncovering a novel approach to affect a disease associated lncRNA in ALT‐driven cancers.

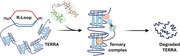

## 1 Introduction

Long noncoding RNAs (lncRNAs), defined as transcripts longer than 200 nucleotides with no protein‐coding capacity, are emerging as pivotal regulators of genome stability, epigenetic programming, and RNA processing.^[^ ^1^, ^2^ ^]^ These transcripts can modulate gene expression, transcription factors, or RNA‐binding proteins, thereby influencing diverse cellular processes and disease pathways.^[^ ^3^ ^]^ Unlike proteins, their functionality is often encoded in higher‐order RNA structures, allowing them to engage dynamically with chromatin modifiers, transcription factors, and RNA‐binding proteins.^[^ ^4^, ^5^ ^]^ In cancer, lncRNA dysregulation has been implicated in nearly every hallmark, including unchecked proliferation, genomic instability, and resistance to therapy.^[^ ^6^, ^7^ ^]^ Despite their critical biological roles, lncRNAs remain largely undrugged due to the inherent challenge of selectively targeting structured RNAs with small molecules.^[^ ^8^ ^]^ Recently, significant efforts have been devoted to drugging RNA structures with small molecules as a novel strategy for therapeutic development.^[^ ^9^ ^]^

**Figure 1:**
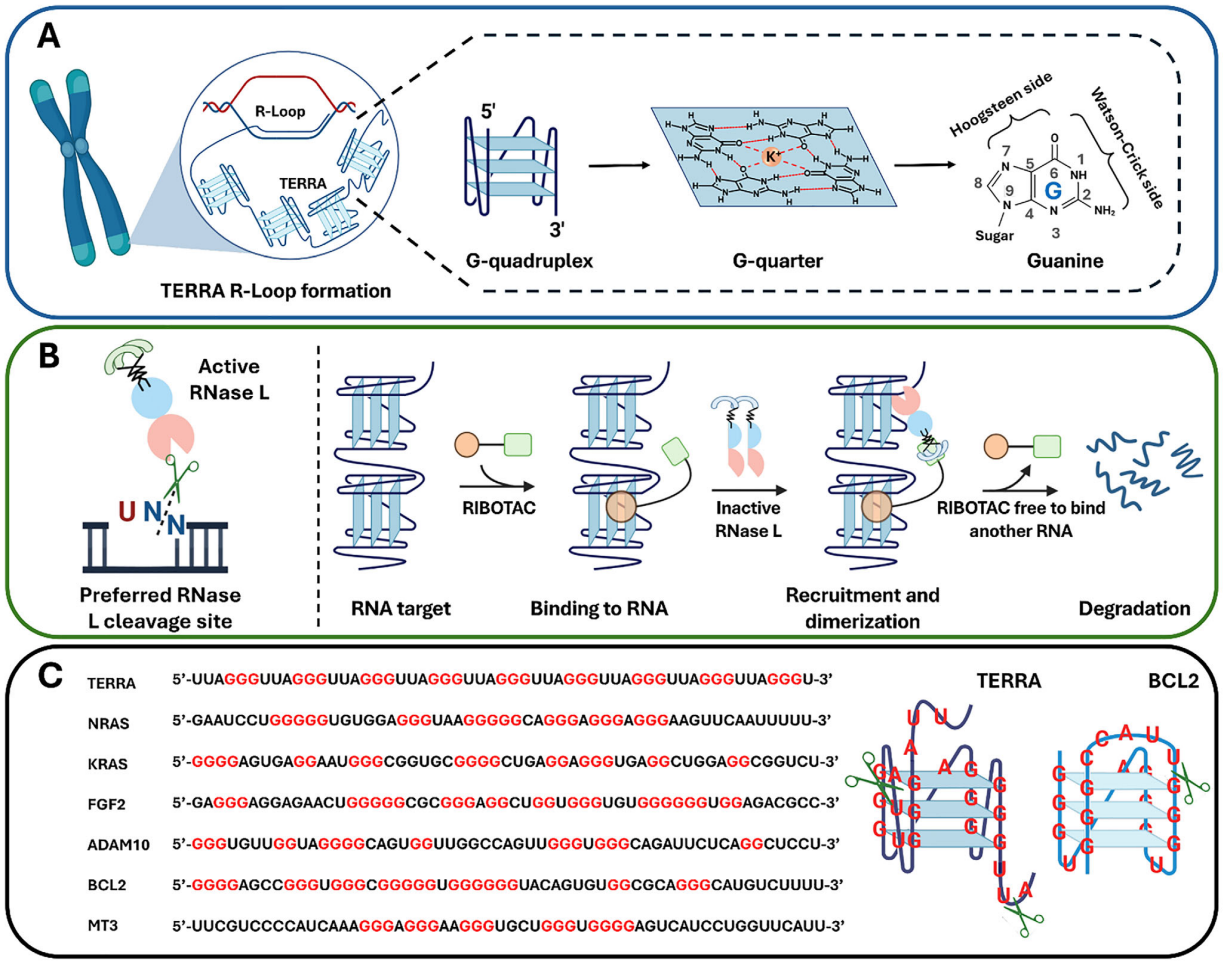
Schematic illustration of TERRA structure and RIBOTAC‐mediated mechanism of action. A) Schematic showing TERRA's localization at telomeres, where it forms R‐loops and folds into stacked RNA G4 structures composed of planar G‐quartets stabilized by Hoogsteen hydrogen bonds. B) RIBOTAC mechanism of action: bifunctional molecules bind the G4 structure and recruit latent RNase L to adjacent UN^N cleavage motifs, promoting RNA degradation through RNase L activation and dimerization. C) Sequence comparison of G4‐forming regions in TERRA and other G4‐containing RNAs (*NRAS*, *KRAS*, *FGF2*, *ADAM10*, *BCL2*, *MT3*). G‐rich sequences capable of forming G‐quartets are highlighted in red. TERRA uniquely displays high G4 density and repetitive UN motifs favorable for RNase L activity.

One unique and not yet fully understood lncRNA is TERRA (telomeric repeat‐containing RNA), a transcript of RNA polymerase II from sub‐telomeric promoters located at the ends of chromosomes, consisting of long stretches of 5′‐UUAGGG‐3′ repeats, with lengths ranging from hundreds to thousands of repeats, such as those found on chromosomes 7p, 13q, 15q, and 20q.^[^ ^10^, ^11^ ^]^ TERRA exhibits a dynamic structure, ranging from highly structured regions containing consecutive G‐quadruplex (G4) to segments that stably hybridize with telomeric DNA, forming R‐loops and contributing to its regulatory functions at telomeres (**Figure** **1**A).^[^ ^12^, ^13^ ^]^ In healthy cells, TERRA contributes to telomere surveillance and replication timing.^[^ ^14^ ^]^ In contrast, in Alternative Lengthening of Telomeres (ALT) cancer cells, which rely on homologous recombination instead of telomerase, TERRA is markedly upregulated.^[^ ^15^, ^16^ ^]^ contributing to the assembly of ALT‐associated PML (Promyelocytic Leukemia) bodies (APBs).^[^ ^17^, ^18^ ^]^

The combination of structural repetitiveness, dense functional topology, and essential roles in ALT telomere maintenance makes TERRA not only biologically intriguing but also a highly compelling target for selective therapeutic intervention.^[^ ^13^ ^]^ Despite its defined structure and interactions, TERRA remains largely underexplored, and its biological role is not yet fully understood.^[^ ^11^ ^]^ Its complex and stable 3D conformation presents significant challenges for precise targeting, particularly by antisense oligonucleotides. This structure arises from guanine‐rich sequences that form G4‐four‐stranded helical motifs stabilized by Hoogsteen hydrogen bonding between stacked guanine tetrads and coordinated monovalent cations, especially potassium (K⁺) (Figure 1A).^[^ ^19^ ^]^ G4s have been extensively studied in DNA, but RNA G4s are more thermodynamically stable due to the absence of competing Watson–Crick base pairing and the rigidity of the ribose backbone.^[^ ^20^, ^21^ ^]^ These motifs are not merely structural artifacts: they are functional regulators of transcription, splicing, RNA localization, and translation.^[^ ^22^ ^]^

To investigate TERRA's role, we aimed to develop molecules that selectively reduce its abundance in ALT cancer cell lines. For targeted degradation of this uniquely structured lncRNA, we have employed RIBOTAC (Ribonuclease‐Targeting Chimera) technology, which links an RNA‐binding small molecule to a recruiter of RNase L, an endoribonuclease activated by small molecules that induce its dimerization and enzymatic activation.^[^ ^23^, ^24^, ^25^ ^]^ RNase L preferentially cleaves RNA at UN motifs (where N represents any ribonucleotide) located in unpaired regions.^[^ ^26^, ^27^ ^]^ Notably, the (UUAGGG)n repeat architecture provides a high density of UN motifs between G4 planes, positioning TERRA as an ideal substrate for RNase L, when brought into proximity by a RIBOTAC molecule.^[^ ^28^ ^]^

Our RIBOTAC design consists of two components: 1) a G4‐binding scaffold based on ISCH, a small molecule with fluorogenic turn‐on properties toward RNA G4 structures,^[^ ^29^ ^]^ and 2) a covalently linked RNase L recruiting module that engages the RNase L active site to trigger site‐specific RNA cleavage upon ternary complex formation (Figure 1B).^[^ ^30^, ^31^, ^32^ ^]^ While G4s are also found in oncogenic transcripts such as *NRAS*, *KRAS*, *FGF2*, *ADAM10*, and *BCL2*,^[^ ^33^, ^34^, ^35^, ^36^, ^37^ ^]^ and have inspired efforts to develop G4‐binding small molecules,^[^ ^38^, ^39^, ^40^ ^]^ distinguishing disease‐associated from physiological G4s remains challenging.^[^ ^41^ ^]^ We therefore focus on the lncRNA TERRA, whose repetitive G4 planes and dense UN‐motif spacing together provide a practical selectivity handle for RIBOTAC design (Figure 1C).

**Scheme 1:**
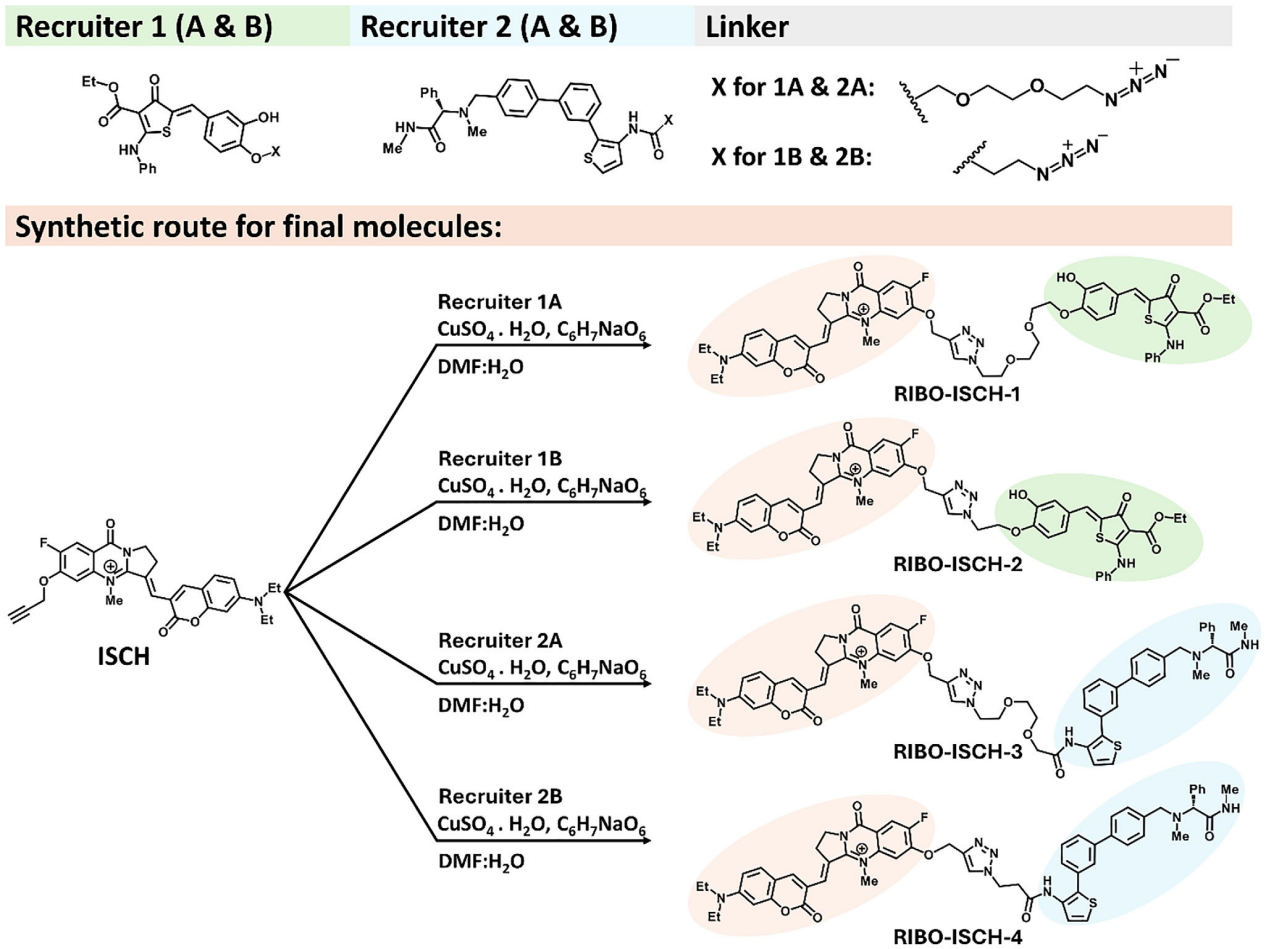
Synthetic routes for RIBOTAC degrader molecules. The G4‐binding scaffold ISCH (highlighted in orange) was conjugated via Cu(I)‐catalyzed click chemistry to RNase L recruiters through two types of linkers: an aliphatic linker or a PEG‐based linker. Recruiter 1 (highlighted in green) and Recruiter 2 (highlighted in blue) represent two distinct RNase L recruiters. For each recruiter, both linker variants were used to yield four RIBOTACs: RIBO‐ISCH‐1 and RIBO‐ISCH‐2 (based on Recruiter 1) and RIBO‐ISCH‐3 and RIBO‐ISCH‐4 (based on Recruiter 2).

In this study, we developed RIBOTACs that selectively degrade TERRA in both telomerase‐positive and ALT‐positive cancer cells. These chimeric molecules specifically recognize TERRA's G4 structure, promoting its targeted RNA cleavage by RNase L without affecting other G4‐containing RNAs or inducing DNA damage. Selective degradation of TERRA disrupts ALT‐associated PML body formation, a hallmark of ALT activity, and suppresses key cancer‐associated phenotypes. Together, our findings highlight RIBOTACs as a promising targeted therapeutic strategy for ALT‐driven cancers and as a precise molecular tool to dissect TERRA's biological functions.

## 2 Results and Discussion

A series of RIBOTACs capable of selectively degrading the G4‐forming long noncoding RNA TERRA was designed and synthesized using the previously reported ISCH scaffold, a selective small‐molecule binder of G4 DNA and RNA structures (Scheme 1).^[^ ^29^ ^]^ Our strategy centered on the modular assembly of ISCH with two chemically distinct RNase L recruiters via copper(I)‐catalyzed azide–alkyne cycloaddition (CuAAC).^[^ ^30^, ^32^ ^]^ This design enabled us to explore the influence of recruiter identity and linker composition on RNase L recruitment and TERRA degradation. The ISCH core was modified to include a terminal alkyne via a synthetic route that retained its G4‐binding capability.

**Figure 2:**
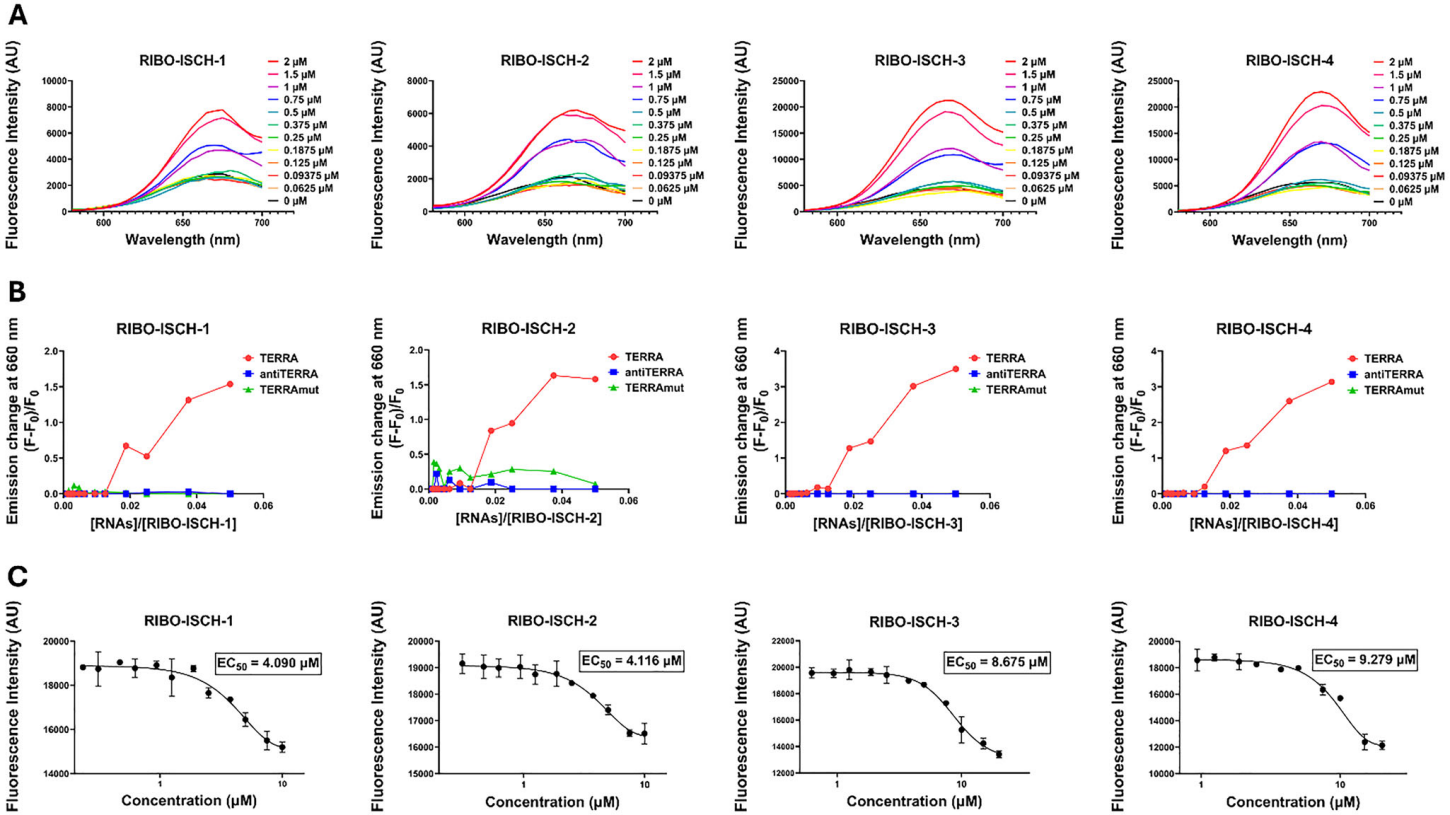
Fluorescence‐based evaluation of G4 binding of the RIBOTAC degraders. A) Emission spectra of RIBO‐ISCH‐1 through RIBO‐ISCH‐4 (40 µm) following titration with increasing concentrations of RNA TERRA (0–2 µm, 24‐point dilution series) (*n* = 3). B) Fold change in fluorescence emission at 660 nm for each compound (40 µm) upon incubation with TERRA compared to non‐G4 controls (TERRAmut and antiTERRA), all at 2 µM concentration (*n* = 3). C) EC_50_ determination based on fluorescence quenching of 5′FAM‐labeled TERRA (2 µm) upon titration with RIBOTACs. Decreasing fluorescence reflects compound binding to the labeled RNA target (*n* = 3).

To assess whether conjugation to RNase L recruiters affected the G4‐binding capacity of the ISCH scaffold, we first evaluated the intrinsic fluorescence properties of the newly synthesized RIBOTACs in response to various nucleic acid structures. Fluorescence titrations were carried out by adding RNA TERRA at increasing concentrations to a fixed concentration of each compound (ISCH, RIBO‐ISCH‐1 through RIBO‐ISCH‐4), followed by spectral scanning to monitor changes in emission intensity. As expected, all compounds exhibited minimal fluorescence in buffer alone, consistent with the low emissive character of the free ligands. However, the addition of RNA or DNA G4 structures, specifically RNA TERRA, DNA TERRA, and RNA MT3, elicited a pronounced fluorescence turn‐on response, with maximal emission centered ≈660 nm (**Figure** **2**A; Figures S1–S5, Supporting Information). In contrast, control sequences lacking G4 potential (antiTERRA and TERRAmut) produced negligible changes in fluorescence, supporting the structural specificity of the interaction (Figure 2B; Figures S1–S5, Supporting Information).

To directly measure binding affinity and selectivity, we titrated RIBO‐ISCH‐1 against several nucleic acid structures: RNA TERRA, RNA MT3, DNA TERRA, TERRAmut (non‐G4 RNA), and anti‐TERRA (non‐G4 DNA). RIBO‐ISCH‐1 exhibited the strongest binding affinity toward TERRA G4 RNA (K_d_ = 1.2 µm). By contrast, its affinity for MT3 RNA G4 and DNA TERRA G4 was weaker (K_d_ = 20.5 and 17.5 µm, respectively), and no measurable binding occurred with the non‐G4 controls. (Figure S6, Supporting Information). These results demonstrate that RIBO‐ISCH‐1 has marked selectivity for TERRA RNA G4 compared to other G4 or non‐G4 structures, showing approximately an order of magnitude higher affinity for RNA TERRA.

To confirm that fluorescence activation was dependent on G4 folding, parallel experiments were conducted under G4‐unfolding conditions (copper sulphate‐containing buffer). Under this condition, RNA sequences that previously activated fluorescence failed to induce any signal enhancement, and instead, the emission intensity dropped to background levels (Figure S7, Supporting Information), underscoring the requirement for a folded G4 conformation for productive binding. These findings indicate that the RIBOTACs preserve the G4‐selective binding and fluorescence turn‐on behavior of the ISCH scaffold and respond sensitively to the conformational status of the nucleic acid target. To quantify binding affinity, EC_50_ values were determined by titrating each compound into a solution of FAM‐labeled RNA TERRA and monitoring the corresponding changes in fluorescence. Interestingly, a concentration‐dependent diminution of the FAM signal was observed upon binding. This decrease suggests a close‐proximity interaction between the ligand and the fluorophore‐labeled RNA G4. The resulting EC_50_ values indicated micromolar binding affinities: 4.090 µm for RIBO‐ISCH‐1, 4.166 µm for RIBO‐ISCH‐2, 8.675 µm for RIBO‐ISCH‐3, and 9.279 µm for RIBO‐ISCH‐4 (Figure 2C).

**Figure 3:**
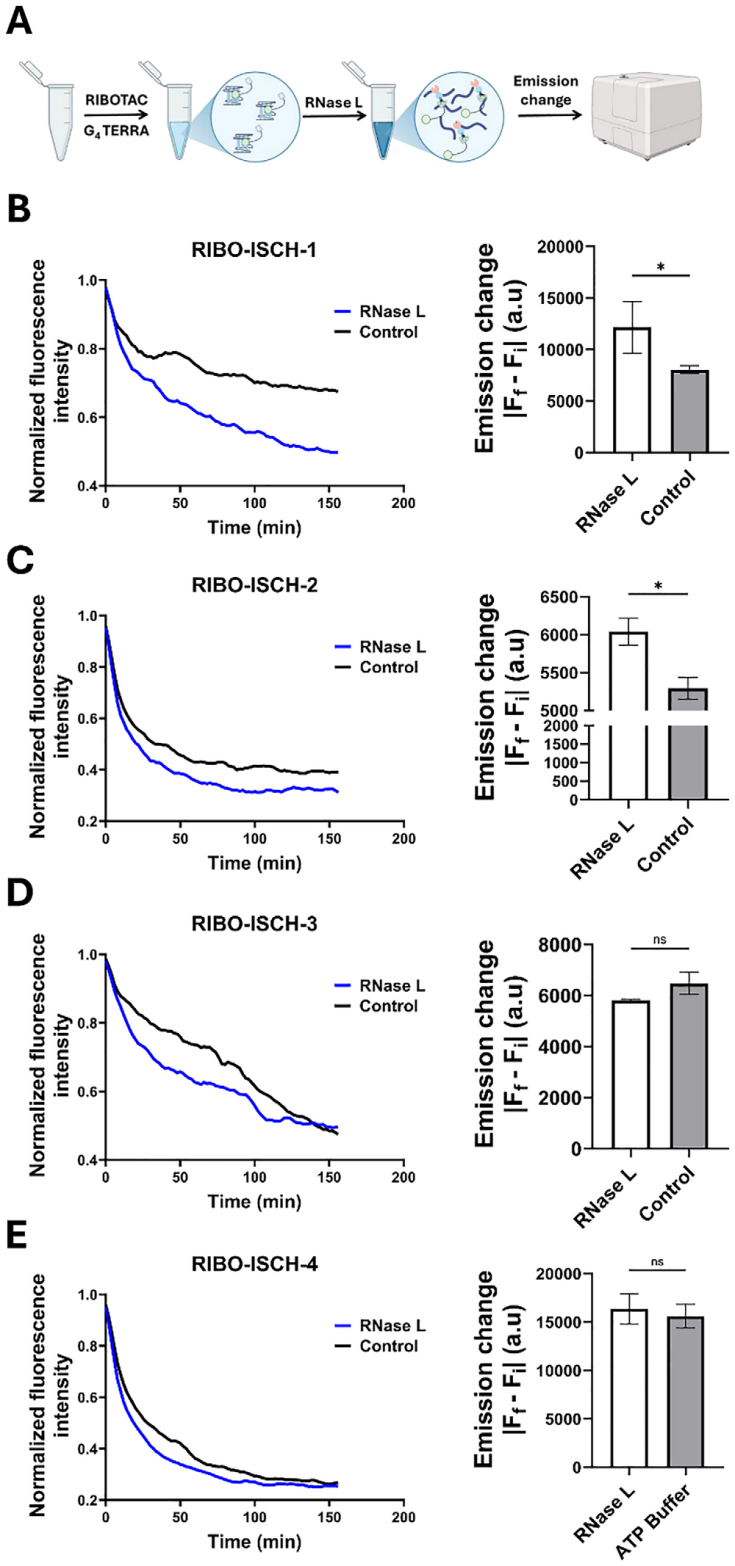
RNase L‐mediated degradation of RNA TERRA induced by RIBOTACs. A) Schematic illustration of the time‐resolved fluorescence assay. Pre‐folded RNA TERRA was incubated with each RIBOTAC, followed by the addition of RNase L, and fluorescence decay was monitored over time. B–E) Normalized fluorescence decay curves over 150 min and corresponding quantification of emission change. Curves represent fluorescence intensity over time in the presence (blue) or absence (black) of RNase L. B) Normalized fluorescence decay curve and quantification for RIBO‐ISCH‐1 (40 µm). C) Normalized fluorescence decay curve and quantification for RIBO‐ISCH‐2 (40 µm). D) Normalized fluorescence decay curve and quantification for RIBO‐ISCH‐3 (40 µm). E) Normalized fluorescence decay curve and quantification for RIBO‐ISCH‐4 (40 µm). Statistical comparison was performed by unpaired *t‐*test (^*^ *p* < 0.05; n.s.: not significant, *n* = 3).

To evaluate whether RIBOTAC binding facilitates RNase L‐mediated RNA degradation, we performed a time‐resolved fluorescence assay using prefolded RNA TERRA incubated with each compound, followed by the addition of RNase L. A decrease in fluorescence intensity was used as a readout for RNA degradation, as cleavage events disrupt the RNA‐RIBOTAC interaction. RNA was pre‐folded in a potassium‐containing buffer to promote G4 formation and incubated with a fixed concentration of each RIBOTAC. Upon addition of RNase L, a time‐dependent decrease in fluorescence was observed. RIBO‐ISCH‐1 and RIBO‐ISCH‐2 demonstrated significantly reduced fluorescence signal in the presence of RNase L compared to buffer‐only controls, indicating effective recruitment of RNase L and subsequent RNA cleavage (**Figure** **3**B,C). In contrast, RIBO‐ISCH‐3 and RIBO‐ISCH‐4 showed no significant difference between RNase L‐treated and control samples (Figure 3D,E), suggesting these variants do not promote RNase L activity under the tested conditions. These results suggest that RIBOTACs incorporating the first recruiter (RIBO‐ISCH‐1 and RIBO‐ISCH‐2) are competent in directing RNase L to cleave the G4‐containing TERRA RNA, while the second recruiter used in RIBO‐ISCH‐3 and RIBO‐ISCH‐4 fails to elicit cleavage, underscoring the importance of recruiter identity in determining RIBOTAC activity. To assess whether background RNase L activity could explain the fluorescence loss, we performed a control in which prefolded TERRA was complexed with the parent ISCH binder and then exposed to RNase L. Fluorescence declined modestly (≈20% over two and a half hours), which can be explained by proximity‐induced quenching or RNase L background cleavage activity. By comparison, RIBO‐ISCH‐1 and RIBO‐ISCH‐2 produced a pronounced ≈50% and ≈60% decrease, respectively, over the same period, indicating recruiter‐dependent engagement of RNase L and RNA degradation (Figure 3B,C; Figure S8, Supporting Information).

**Figure 4:**
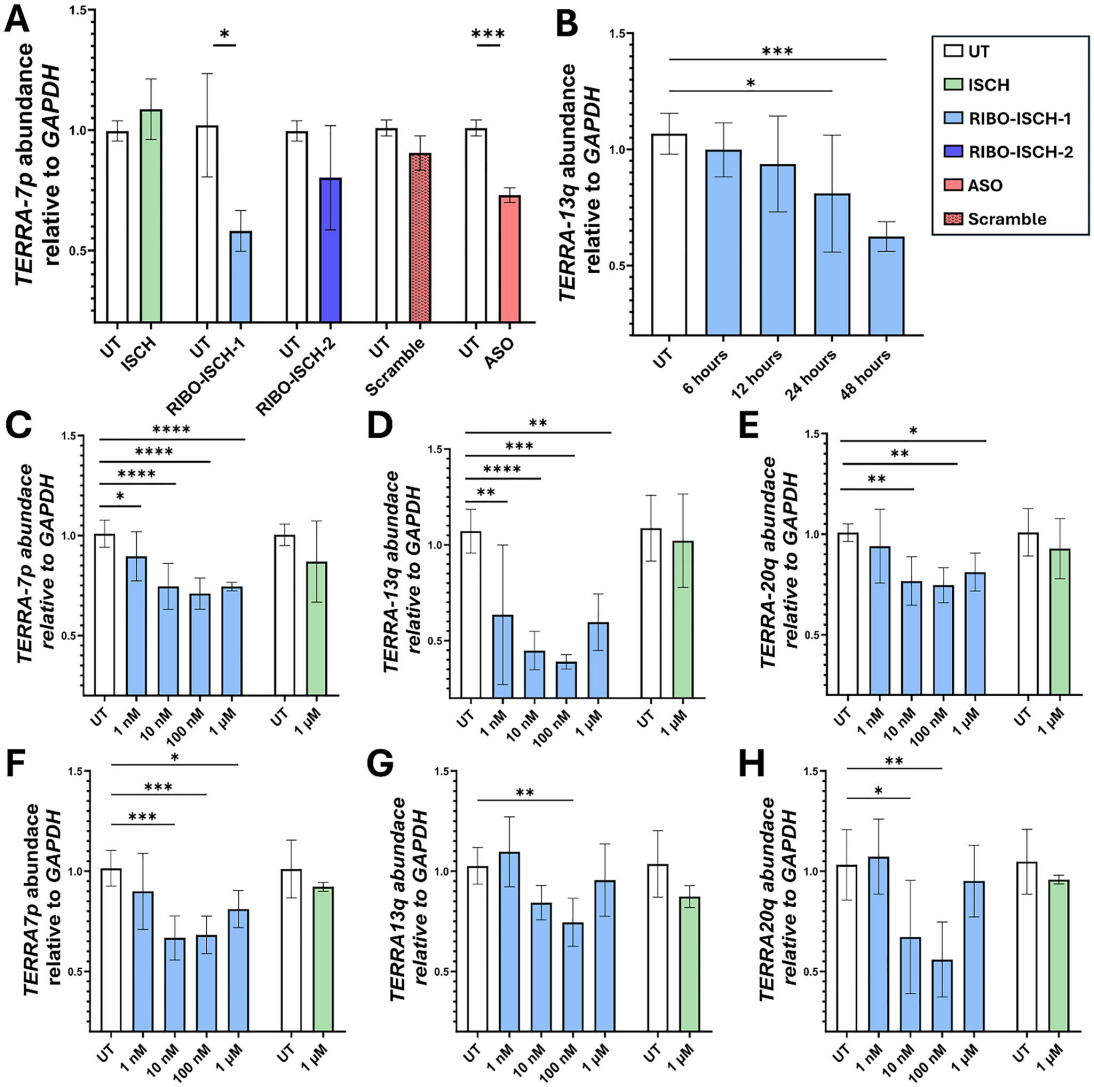
Relative abundance of TERRA transcript levels following treatment with RIBOTACs and controls in HeLa and U2OS cells. A) Relative abundance of TERRA‐7p following treatment in HeLa cells with ISCH (100 nM), RIBO‐ISCH‐1 (100 nM), Scr (50 nM), or ASO (50 nM) for 48 h as determined by RT‐qPCR. Data are normalized by the mean expression level in untreated control cells. Statistical comparison was performed by unpaired t‐test (^***^ *p* < 0.001; ^*^ *p* < 0.05). B) Relative abundance of TERRA‐13q following time‐dependent treatment in HeLa cells with RIBO‐ISCH‐1 (100 nM) as determined by RT‐qPCR. Data are normalized by the mean expression level in untreated control cells. Statistical comparison was performed by one‐way ANOVA (^***^ *p* < 0.001; ^*^ *p* < 0.05). C–E) Relative abundance of different TERRA loci levels following dose‐response treatment in HeLa cells with RIBO‐ISCH‐1 for 48 h, as determined by RT‐qPCR. Data are normalized by the mean expression level in untreated control cells. F–H) Relative abundance of different TERRA loci levels following dose‐response treatment in U2OS cells with RIBO‐ISCH‐1 for 48 h, as determined by RT‐qPCR. Data are normalized by the mean expression level in untreated control cells. Statistical comparison was performed by one‐way ANOVA (^****^ *p* < 0.0001; ^***^ *p* < 0.001; ^**^ *p* < 0.01; ^*^ *p* < 0.05). All experiments were performed in triplicate (*n* = 3).

Following the observation that RIBO‐ISCH‐1 and RIBO‐ISCH‐2 facilitate RNase L‐mediated cleavage of TERRA in vitro, we next sought to evaluate their activity in a cellular context. To this end, we selected HeLa cells, which, though telomerase‐positive rather than ALT, exhibit high TERRA abundance, allowing us to test whether RIBO‐ISCH‐1/2 activity is ALT‐independent and generalizable across cancer cell types. Cells were treated with ISCH, RIBO‐ISCH‐1, or RIBO‐ISCH‐2, alongside two RNA‐targeting controls: a TERRA‐specific antisense oligonucleotide (ASO) and a scrambled ASO control (Scr). *TERRA‐7p* levels were quantified by RT‐qPCR using locus‐specific primers (**Figure** **4**A; Figures S9 and S10, Supporting Information). Treatment with ISCH alone did not alter TERRA levels relative to untreated cells (UT), confirming that binding alone does not lead to RNA degradation. Interestingly, among the bifunctional compounds, only RIBO‐ISCH‐1 induced a significant reduction in *TERRA‐7p* abundance, while RIBO‐ISCH‐2 had no effect despite demonstrating RNase L recruitment in vitro (Figure 4A). Notably, the extent of degradation observed with RIBO‐ISCH‐1 was comparable to that achieved with the positive control ASO, and both were clearly distinguishable from the nontargeting Scr. These data highlight the requirement for productive RNase L recruitment and cellular compatibility to achieve effective RIBOTAC‐mediated RNA knockdown. To further characterize the temporal dynamics of TERRA degradation, we conducted a time‐course experiment with RIBO‐ISCH‐1 and monitored *TERRA‐7p*, *TERRA‐13q*, and *TERRA‐20q* levels at 6, 12, 24, and 48 h post‐treatment (Figure 4B; Figure S11, Supporting Information).

While a significant reduction was observed as early as 24 h, a statistically significant and time‐dependent decrease in TERRA abundance was evident, with maximal knockdown achieved at 48 h observed among multiple TERRA loci. This progressive decline suggests that cellular RIBOTAC activity accumulates over time and may reflect the combined effects of RNase L recruitment and endogenous RNA turnover mechanisms. We next performed a dose‐response analysis of RIBO‐ISCH‐1 across multiple TERRA loci. In HeLa cells, RIBO‐ISCH‐1 induced a clear, dose‐dependent reduction of TERRA transcripts at 7p, 20q, and 13q loci (Figure 4C–E). Statistically significant knockdown was observed at concentrations starting from 10 nM, with maximal effects at 100 nM. Interestingly, an increase was observed at 1 µm, likely due to a hook effect, a known mechanism in targeted degradation of hybrid molecules.^[^ ^42^, ^43^ ^]^

In contrast, treatment with ISCH alone did not result in any significant change in TERRA expression, reinforcing that binding alone is not sufficient for degradation and that enzymatic recruitment is required.

The effects of RIBO‐ISCH‐1 were comparable to the ASO (Figure S12, Supporting Information), highlighting the utility of this nonsequence‐based degrader for targeting structured lncRNAs. To determine whether RIBO‐ISCH‐1 retains activity in ALT‐positive cells, we performed the same dose‐response experiments in U2OS cells, which exhibit elevated TERRA expression due to their telomerase‐independent telomere maintenance mechanism. As expected, RIBO‐ISCH‐1 reduced transcript abundance at *TERRA‐7p*, *13q*, *20q*, and *15q* loci (Figure 4F–H; Figure S13, Supporting Information) in a dose‐dependent manner. Again, 100 nM was the most effective dose, with no decrease observed upon treatment with ISCH. These results indicate that RIBO‐ISCH‐1 remains active in the unique chromatin environment of ALT cells, demonstrating broad applicability across cancer subtypes.

**Figure 5:**
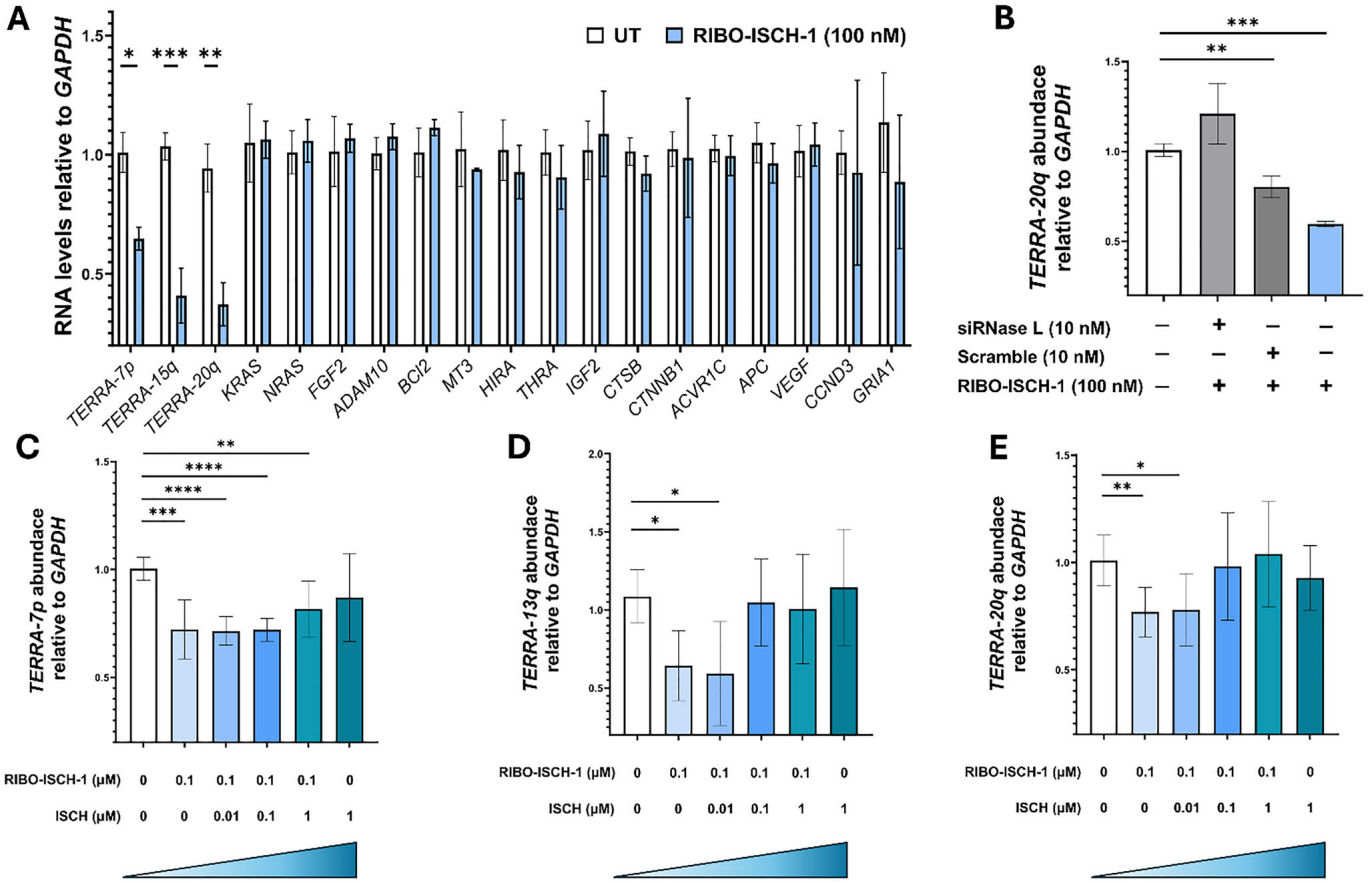
Selectivity, RNase L dependence, and competition binding of RIBO‐ISCH‐1 in HeLa and U2OS cells. A) Relative abundance of *TERRA* and other G4‐containing transcripts (*NRAS*, *KRAS*, *FGF2*, *ADAM10*, *BCL2*, *MT3, VEGFA, APC, ACVR1C, CCND3, CTNNB1, CTSB, GRIA1, HIRA, IGF2, and THRA*) following treatment with RIBO‐ISCH‐1 (100 nM) for 48 h in U2OS cells, as determined by RT‐qPCR. Data are normalized by the mean expression level in untreated control cells. Statistical comparisons were performed using unpaired *t‐*test (^*^ *p* < 0.05; ^**^ *p* < 0.01; ^***^ *p* < 0.001). B) Relative abundance of *TERRA‐20q* following treatment in HeLa cells with RIBO‐ISCH‐1 (100 nM) for 48 h in the presence or absence of siRNA targeting RNase L (10 nM) or nontargeting scramble control (10 nM), as determined by RT‐qPCR. Data are normalized by the mean expression level in untreated control cells. Statistical comparisons were performed using unpaired *t‐*test (^**^ *p* < 0.01; ^***^ *p* < 0.001). C–E) Relative abundance of different TERRA loci levels following treatment in HeLa cells with RIBO‐ISCH‐1 (100 nM) and increasing concentrations of ISCH (0, 10, 100, or 1000 nM), as determined by RT‐qPCR. Data are normalized by the mean expression level in untreated control cells. Statistical comparison was performed by one‐way ANOVA (^****^ *p* < 0.0001; ^***^ *p* < 0.001; ^*^ *p* < 0.01; ^*^ *p* < 0.05). All experiments were performed in triplicate (*n* = 3).

To evaluate the selectivity of RIBO‐ISCH‐1 for TERRA versus general rG4 targeting, we queried the G4RNA database (Scott lab) and retained entries with experimental evidence for rG4 formation (“True”); candidates were then ranked by QGRS Mapper score, and the top set was chosen for RT‐qPCR. The panel comprised *NRAS*, *KRAS*, *FGF2*, *BCL2*, *ADAM10*, *MT3*, *VEGFA*, *APC*, *ACVR1C*, *CCND3*, *CTNNB1*, *CTSB*, *GRIA1*, *HIRA*, *IGF2*, and *THRA* (Figure S14, Supporting Information).^[^ ^38^, ^44^ ^]^ Treatment with RIBO‐ISCH‐1 at 100 nM for 48 h selectively reduced TERRA levels in both U2OS (**Figure** **5**A) and HeLa (Figure S15, Supporting Information) cells, without affecting the expression of the other G4‐containing transcripts tested. In contrast, RIBO‐ISCH‐2, which was inactive against TERRA in cells, elicited modest reductions in non‐TERRA G4 RNAs such as *NRAS*, *FGF2*, *ADAM10*, and *BCL2* (Figure S16A, Supporting Information) indicating that its recruitment module may favor alternate RNA structures or conformations, the difference in cellular activity between the two compounds underscores the critical role of linker architecture: the flexible, hydrophilic PEG linker in RIBO‐ISCH‐1 may facilitate optimal spatial positioning for ternary complex formation with RNase L, whereas the rigid aliphatic linker in RIBO‐ISCH‐2 could hinder productive engagement. As expected, ISCH, used at the same concentration of 100 nM, showed no significant effect on any of the transcripts assessed, including TERRA, in either cell line (Figure S16B,C, Supporting Information). These findings demonstrate that RIBO‐ISCH‐1 is highly selective for TERRA over other G4 RNAs, likely due to the presence of repeat G4 structures unique to TERRA. This selective degradation highlights the utility of RIBO‐ISCH‐1 as a chemical probe for targeting TERRA in TERRA‐associated pathologies. Taken together, these data confirm that RIBO‐ISCH‐1 acts as a potent RIBOTAC capable of inducing degradation of endogenous TERRA in both telomerase‐ and ALT‐positive cancer cells. The activity is both time‐ and concentration‐dependent, robust across multiple genomic loci, and comparable in efficacy to a sequence‐specific antisense oligonucleotide.

We next sought to evaluate whether the observed TERRA degradation was dependent on RNase L enzymatic activity. Cells were pre‐treated with siRNA targeting RNase L (siRNase L), a nontargeting scramble control (Scr), or left untreated, followed by RIBO‐ISCH‐1 treatment. RT‐qPCR analysis showed that silencing RNase L abolished the TERRA knockdown effect (Figure 5B), confirming that degradation is RNase L‐dependent. To validate the efficiency and specificity of RNase L silencing, we confirmed knockdown at the mRNA level by RT‐qPCR and western blot (Figure S17A,B, Supporting Information). To ensure that the siRNA treatment did not nonspecifically affect other gene transcripts, we also measured the expression of unrelated G4‐containing genes following siRNase L transfection. No significant changes were observed in these control transcripts, indicating that RNase L depletion did not affect TERRA abundance (Figure S17C, Supporting Information). In a separate mechanistic evaluation, we examined whether RIBO‐ISCH‐2, also operated via RNase L. Using *FGF2* as a representative transcript, we found that siRNase L pretreatment diminished the RIBO‐ISCH‐2‐induced knockdown of *FGF2*, indicating that this compound also requires RNase L activity to mediate RNA degradation (Figure S18, Supporting Information). These findings strongly support that the RIBOTAC mechanism relies on RNase L enzymatic activity for targeted RNA cleavage and is not the result of off‐target effects or transcriptional suppression. To determine whether RIBO‐ISCH‐1 and its parent scaffold ISCH engage the same RNA binding site in cells, we conducted a competition assay in which cells were pre‐treated with increasing concentrations of ISCH, followed by a fixed concentration of RIBO‐ISCH‐1. RT‐qPCR analysis showed that ISCH pre‐treatment attenuated the RNA‐degrading activity of RIBO‐ISCH‐1 in a dose‐dependent manner (Figure 5C–E), leading to partial or complete rescue of TERRA levels at higher ISCH concentrations. These results indicate that ISCH competes with RIBO‐ISCH‐1 for binding to TERRA, suggesting that both molecules recognize the same or overlapping binding pocket within the G4 structure. This functional competition highlights the specificity of the RIBOTAC for its structured RNA target and confirms that effective degradation requires direct target engagement through the G4‐binding module.

**Figure 6:**
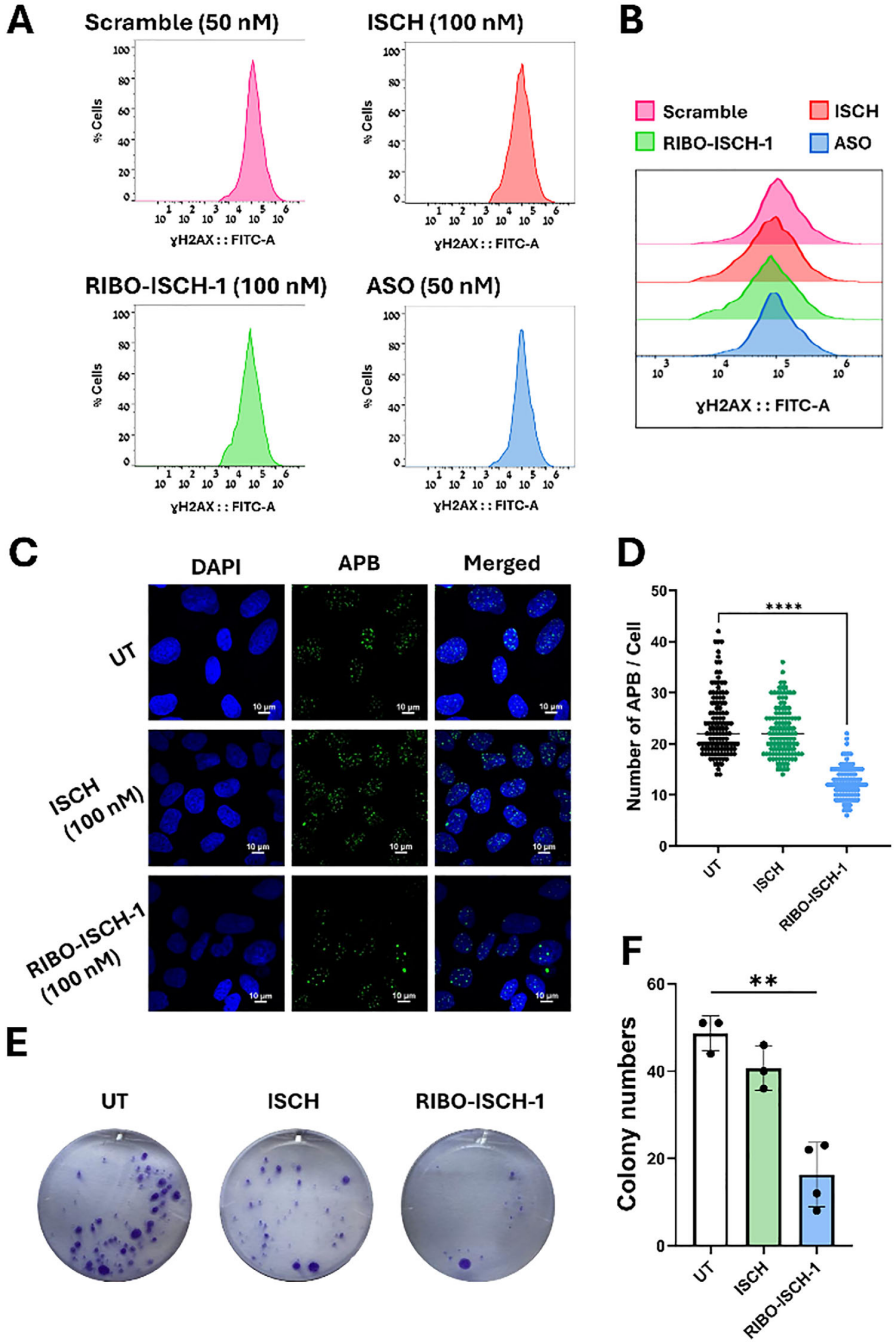
Evaluation of DNA damage, APBs formation, and clonogenic potential following treatment with RIBO‐ISCH‐1. A) Representative flow cytometry histograms showing γH2AX fluorescence in U2OS cells treated with Scr (50 nM), ISCH (100 nM), RIBO‐ISCH‐1 (100 nM), or ASO (50 nM) for 48 h (*n* = 3). B) Overlay of γH2AX fluorescence intensity histograms comparing all treatment conditions. C) Immunofluorescence images of ALT‐associated PML bodies (APBs) in U2OS cells stained with anti‐PML antibody (green) and DAPI (blue) after treatment with ISCH (100 nM) or RIBO‐ISCH‐1 (100 nM) for 48 h. Scale bar = 10 nm. D) Quantification of APBs number per nucleus in treated U2OS cells. (*n* = 120 cells for each treatment over three different experiments), statistical comparison was performed by a two‐tailed unpaired *t‐test* (^****^ *p* < 0.0001). E) Representative images of crystal violet–stained U2OS cell colonies after treatment with ISCH (1 µm) or RIBO‐ISCH‐1 (1 µm). F) Quantification of colony numbers over 4 different experiments. Statistical analysis was performed by unpaired *t‐test* (^*^ *p* < 0.05).

The next step was to confirm whether the intracellular fluorescence of RIBO‐ISCH‐1 derives from RNA or DNA engagement in cells. We performed a nuclease‐sensitivity assay; U2OS cells treated with RIBO‐ISCH‐1 were exposed to DNase I or RNase A, and the change in fluorescence was recorded. RNase A significantly decreased the fluorescence signal, whereas DNase I had no significant effect relative to RIBO‐ISCH‐1 alone (Figure S19A,B, Supporting Information), indicating that the cellular signal primarily reflects binding to RNA G4 rather than DNA G4. With this specificity established, we next assessed whether RIBO‐ISCH‐1 impacts genomic DNA integrity. We evaluated DNA damage using a γH2AX‐based flow cytometry assay in U2OS cells. γH2AX is a well‐established marker of DNA double‐strand breaks, and increased fluorescence intensity correlates with the presence of DNA damage. This experiment was particularly important given that RIBO‐ISCH‐1 binds G4 structures, which are present in both RNA and DNA; therefore, it was critical to determine whether its activity extends to genomic DNA. Importantly, no significant shift in γH2AX fluorescence intensity was observed across any treatment group, including RIBO‐ISCH‐1, ISCH, or the positive control (**Figure** **6**A). ASO, which also promotes depletion of TERRA, was included as a positive control to ensure that any DNA damage linked to TERRA depletion would be detected. Observing comparable results for both RIBO‐ISCH‐1 and ASO strengthens the conclusion that reducing TERRA levels does not elicit a DNA damage response. Overlay of histograms further confirmed the absence of a detectable DNA damage response (Figure 6B), suggesting that RIBO‐ISCH‐1 does not induce global DNA cleavage despite its capacity to bind G4 structures. These findings indicate that the compound exhibits RNA selectivity, as TERRA is targeted for degradation while G4‐forming DNA remains unaffected. To examine whether TERRA depletion alters ALT‐associated homologous recombination (HR) pathways, we measured levels of RAD51 and FANCD2 by Western blot in U2OS cells treated for 48 h. RAD51 is the central recombinase that mediates strand invasion and contributes to ALT telomeric HR (e.g., T‐SCE and break‐induced telomere synthesis),^[^ ^45^ ^]^ whereas FANCD2 is a Fanconi‐anemia pathway effector that responds to replication stress and localizes to APBs to coordinate telomeric repair in ALT cells.^[^ ^46^, ^47^ ^]^ Protein abundance was unchanged following RIBO‐ISCH‐1 (100 nM) or ISCH (100 nM) relative to untreated cells or control groups (Figure S20A,B, Supporting Information). Together with the absence of γH2AX induction, these findings indicate that RIBO‐ISCH‐1 does not trigger broad DNA‐repair activation, supporting a selective, RNA‐directed mechanism (TERRA targeting) rather than direct DNA engagement.

We next examined the impact of RIBO‐ISCH‐1 on hallmarks of ALT‐positive cancer cells, including the presence of ALT‐associated PML bodies and the ability to form colonies. APBs are nuclear foci formed by the colocalization of PML protein with telomeric DNA and recombination proteins and are considered a defining feature of ALT activity. Immunofluorescence staining in U2OS cells revealed a 50% reduction in the number of APBs upon treatment with RIBO‐ISCH‐1, while ISCH had no measurable effect (Figure 6C). Quantification of APBs confirmed this reduction (Figure 6D), suggesting that TERRA depletion disrupts the formation or stability of APBs. To determine whether this effect was specific to ALT cells, we also performed immunofluorescence staining in HeLa cells, which showed a similar reduction in PML body number following RIBO‐ISCH‐1 treatment (Figure S21, Supporting Information), indicating that the effect is not restricted to ALT‐positive backgrounds.

Finally, to determine whether RIBO‐ISCH‐1 impacts the long‐term proliferative capacity of ALT cells, we performed a colony formation assay. U2OS cells treated with RIBO‐ISCH‐1 formed significantly fewer and smaller colonies compared to untreated or ISCH‐treated controls (Figure 6E), with quantification showing a clear decrease in clonogenic potential over a 21‐day experiment (Figure 6F). This phenotype is consistent with impaired ALT‐mediated telomere maintenance following selective TERRA degradation.^[^ ^13^ ^]^ We next evaluated whether RIBO‐ISCH‐1 affected proliferation rate and observed a modest, but statistically significant, decrease at 10 µm (Figure S22A, Supporting Information). To determine whether this reduction in proliferation and colony formation resulted from apoptosis, we examined caspase‐3 activation. Cleaved caspase‐3 was detected only in the doxorubicin positive control, with no signal in untreated, ISCH‐treated, RIBO‐ISCH‐1‐treated, ASO‐treated, or scramble control samples (Figure S22B, Supporting Information). Therefore, the impaired colony formation likely arises from a nonapoptotic, slow‐acting mechanism centered on TERRA targeting, rather than from apoptotic cell death.

Collectively, these findings show that while RIBO‐ISCH‐1 does not induce DNA damage, it disrupts key features of the ALT phenotype, namely, the formation of PML bodies and the ability to sustain colony growth, supporting a functional role for TERRA in maintaining ALT activity and cellular proliferation.

## 3 Conclusion

In summary, we have developed a RIBOTAC degrader that selectively cleaves the G4‐portion of the lncRNA TERRA by harnessing a bifunctional approach combining G4‐specific binding with RNase L recruitment. While the parent compound recognizes both RNA and DNA G4 structures, its transformation into a RIBOTAC reprograms its activity exclusively toward RNA, resulting in selective degradation of TERRA without affecting genomic DNA. Among the series, RIBO‐ISCH‐1 emerged as a potent and selective degrader that effectively reduces TERRA levels across multiple chromosomal loci in both telomerase‐positive and ALT‐positive cancer cells. Notably, despite micromolar binding affinity to TERRA, RIBO‐ISCH‐1 induces degradation at nanomolar concentrations, underscoring the catalytic nature of RNase L recruitment. This amplification highlights the power of RIBOTAC design, where a single binding event can trigger multiple cleavage events, enhancing potency through enzymatic turnover. This selectivity is driven by the repetitive G4 motifs uniquely enriched in TERRA, enabling precise engagement and RNase L‐mediated cleavage. The resulting degradation suppresses ALT‐associated phenotypes such as APB formation and clonogenic growth. Unlike previously described antisense strategies, this small‐molecule‐based approach allows for modular tuning of activity and structure–function relationships. Importantly, RIBO‐ISCH‐1 spares unrelated G4‐containing RNAs, highlighting its utility as a precision tool to dissect TERRA biology. Beyond its immediate impact, this technology enables the development of novel chemical tools to study the still largely unexplored roles of TERRA and presents a promising therapeutic strategy for targeting ALT‐driven cancers. Although our functional readouts center on ALT, TERRA also regulates telomere biology in telomerase‐positive cancers and in normal tissues. Because TERRA both restrains telomerase and supports telomeric R‐loop homeostasis, its degradation is expected to be context‐dependent: modest reduction may alleviate telomeric R‐loop–driven replication stress, whereas deeper depletion could de‐repress telomerase. Accordingly, deployment in telomerase‐positive tumors will require tuned dosing and targeted delivery. These considerations broaden the relevance of our approach beyond ALT while acknowledging potential liabilities for normal telomere maintenance.

## 4 Experimental Section

### Cell Culture

HeLa cells (AC‐free, ECACC 08011102) were obtained from the European Collection of Authenticated Cell Cultures and cultured in DMEM medium containing Earle's salts and L‐glutamine, supplemented with 10% (v/v) fetal bovine serum (FBS; Sigma‐Aldrich, F9665) and 1% penicillin/streptomycin solution (Diagnovum, D910). U2OS cells (ATCC HTB‐96) were purchased from the American Type Culture Collection and maintained in McCoy's 5A medium with L‐glutamine, supplemented with 10% (v/v) fetal bovine serum (FBS; Sigma‐Aldrich, F9665) and 1% penicillin/streptomycin solution (Diagnovum, D910). Both cell lines were cultured at 37 °C in a humidified incubator with 5% CO_2_, and were confirmed to be mycoplasma‐free before use.

### Fluorescence Studies

The binding selectivity of synthesized compounds toward G‐quadruplex‐forming RNA (RNA TERRA, DNA TERRA, and MT3) over non‐G4 controls (anti‐TERRA and TERRA‐mutant) was evaluated by monitoring changes in fluorescence intensity. Briefly, increasing concentrations of RNA or DNA sequences were titrated into a fixed concentration of compounds in 1× folding buffer (10 mM Tris‐HCl, pH 7.6, and 100 mm KCl). Prior to titration, nucleic acids were folded by heating to 95 °C for 5 min and gradually cooled to room temperature. Fluorescence measurements were performed using the Synergy H1 Hybrid Multi‐Mode Microplate Reader (BioTek, Agilent), and changes in fluorescence were analyzed to assess the relative binding preferences of each compound.

Half‐maximal effective concentration (EC_50_) measurements for the interaction between synthesized compounds and 5'–FAM–labeled TERRA RNA were performed by monitoring changes in fluorescence intensity as a function of compound concentration. Briefly, the RNA was folded in 1× folding buffer (10 mm Tris‐HCl, pH 7.6, and 100 mm KCl) by heating at 95 °C for 5 min, followed by slow cooling to room temperature. Compound solutions were prepared in the same buffer. Compounds were titrated into a fixed concentration of RNA (1 µm), starting from 20 µm and serially diluted (1:2) down to 7.32 nM, yielding a total of 24 concentrations. EC_50_ values were determined from plots of compound concentration versus fluorescence change, measured using the Synergy H1 Hybrid Multi‐Mode Microplate Reader (BioTek, Agilent).

For the nuclease sensitivity assay, cells were seeded in 96‐well plates and, at ≈90% confluency, fixed with 4% (w/v) paraformaldehyde in PBS at room temperature, washed ×3 with cold PBS, and permeabilized with 0.2% Triton X‐100 for 30 min at 37 °C. After washing, cells were incubated for 3 h with DNase I or RNase A (200 U mL^−1^ in PBS) or left untreated, followed by the addition of RIBO‐ISCH‐1 (20 µM). Kinetic fluorescence readouts were collected on a Synergy H1 Hybrid Multi‐Mode Microplate Reader (BioTek, Agilent) for 2.5 h using the appropriate excitation/emission settings for the fluorophore, and the fluorescence change (Δ*F* relative to the pre‐addition baseline) was calculated.

For determining binding affinity (Kd), RNA TERRA, DNA TERRA, RNA MT3, TERRA‐mutant, or anti‐TERRA were folded in 1× folding buffer (10 mm Tris‐HCl, pH 7.6, 100 mm KCl) by heating at 95 °C for 5 min followed by slow cooling to room temperature. Increasing concentrations of the folded nucleic acid were titrated into a fixed concentration of RIBO‐ISCH‐1 prepared in the same buffer. Titrations comprised 24 twofold serial dilutions from a top strand concentration selected to reach signal saturation. Fluorescence was recorded on a Synergy H1 Hybrid Multi‐Mode Microplate Reader (BioTek, Agilent).

### Fluorescence‐Based In Vitro RNA Cleavage Assay

RNA degradation was assessed by monitoring the decrease in fluorescence upon addition of RNase L to prefolded RNA‐compound mixtures. Briefly, RNA was folded in 1× folding buffer (10 mm Tris‐HCl, pH 7.6, and 100 mm KCl) by heating at 95 °C for 5 min, followed by slow cooling to room temperature. Folded RNA was then mixed at a fixed concentration with synthesized compounds at a fixed concentration. RNase L was prepared in its activation buffer (7 mm β‐mercaptoethanol, 50 µm ATP, and 1 mm MgCl_2_), heated at 95 °C for 5 min, and rapidly cooled on ice for 5 min. It was then added to one set of triplicate wells. A second triplicate containing RNA, compound, and activation buffer (without RNase L) served as a negative control. Changes in fluorescence were measured using the Synergy H1 Hybrid Multi‐Mode Microplate Reader (BioTek, Agilent).

### General Protocol for mRNA RT‐qPCR

HeLa or U2OS cells were seeded in 6‐well plates at a density of ≈250 000 cells per well (60–70% confluency) and incubated for 12 h. Cells were then treated with the indicated concentrations of synthesized compounds for the specified durations. Following treatment, total RNA was extracted using the Quick‐RNA Miniprep Kit (Zymo Research) according to the manufacturer's protocol, including on‐column DNase I treatment. Reverse transcription was performed on 1 µg of total RNA using the LunaScript RT SuperMix Kit (New England BioLabs, cat #M3010), following the manufacturer's instructions. Approximately 30 ng of cDNA was used for each qPCR reaction, which was carried out using the Luna Universal qPCR Master Mix (New England BioLabs, cat. #E3010G) on a CFX Opus 384 Real‐Time PCR System (Bio‐Rad). Relative expression levels of *NRAS, KRAS, FGF2, BCL2, ADAM10, MT3, VEGFA, APC, ACVR1C, CCND3, CTNNB1, CTSB, GRIA1, HIRA, IGF2, THRA, RNase L, TERRA7p, TERRA13q, TERRA15q*, and *TERA20q* were quantified by normalizing to GAPDH expression using the ΔΔCt method.

### General Protocol for Western Blotting

U2OS cells were grown in 6‐well plates at ≈60% confluency in complete growth medium, and the cells were treated with different synthesized compounds and controls at the indicated concentration or vehicle for 48 h. Total protein was extracted using RIPA cell lysis buffer (BioPrep) containing protease inhibitor, and protein concentration was measured using BCA protein assay (Sigma–Aldrich) according to the manufacturer's protocol. Approximately 50 µg of total protein was resolved on a 10% SDS‐acrylamide gel and then transferred to a PVDF membrane (Immobilon FL transfer membrane‐ Merck). The membrane was washed with 1X Tris‐buffered saline (TBS) containing 0.1% (v/v) Tween‐20 (TBST; Tris‐base, pH 7.6, NaCl and Tween‐20), and then blocked in 1x TBST containing 5% (w/v) BSA for 2 h at room temperature. The membrane was then incubated with 1:1000 dilution of rabbit anti‐FANCD2 (Abcam: ab108928), 1:1000 dilution of mouse anti‐RAD51 (Abcam: ab88572), 1:1000 dilution of rabbit anti‐cleaved caspase‐3 (Cell Signaling Technology: #9661), 1:1000 dilution of rabbit anti‐RNase L (Cell Signaling Technology: #27281), 1:1000 dilution of rabbit anti‐GAPDH (Cell Signaling Technology:14C10) or 1:1000 dilution of rabbit anti‐Vinculin (Cell Signaling Technology: CS13901) in 1x TBST containing 5% (w/v) BSA overnight at 4 °C. The membrane was then washed with 1x TBST and incubated with 1:10 000 anti‐rabbit IgG horseradish‐peroxidase secondary antibody conjugate (Cell Signaling: CS7074) or anti‐mouse IgG horseradish‐peroxidase secondary antibody conjugate (Cell Signaling: CS7076) at room temperature for 2 hr. After washing three times with 1× TBST (10 min per wash), the target protein was detected by using SuperSignal West Pico PLUS Chemiluminescent Substrate (Thermo Scientific) on Azure C300 imaging System. The fold change of the target protein expression was calculated by normalizing the band intensity to housekeeping protein Vinculin or GAPDH band intensity using ImageJ.

### Flow Cytometry

U2OS cells were seeded onto 6‐well plates and treated with ISCH, RIBO‐ISCH‐1, ASO, or Scramble for 48 h, then were collected into 2 mL microcentrifuge tubes and washed with 1 mL of FACS buffer (0.1% BSA in PBS), followed by centrifugation at 400 × g for 5 min. The cell pellets were resuspended in 200 µL of 2% paraformaldehyde (PFA) in PBS and incubated at room temperature for 10 min in the dark. After fixation, cells were washed twice with FACS buffer and resuspended in 200 µL of 0.2% Triton X‐100 in PBS for permeabilization. Samples were incubated for 10 min at room temperature, followed by a PBS wash. Cells were then incubated with anti‐γH2AX primary antibody (Cell Signaling, cat. CST‐ 9718, 1:200 dilution in FACS buffer (500 µL per sample)) for 1 h on ice. Following primary antibody staining, cells were washed 2–3 times with FACS buffer and incubated with Alexa Fluor–conjugated secondary antibody (Abcam, Cat. AB‐ab150077, 1:2000 dilution in FACS buffer) for 45 min on ice, protected from light. After staining, cells were washed 2–3 times with FACS buffer and resuspended in 1 mL PBS. Prior to acquisition, samples were passed through a 70 µm cell strainer into FACS tubes and kept on ice. Data were acquired using CytoFLEX cytometer and analysis was performed using FlowJo software (FlowJo_v10.10). cytometry software.

### Fluorescence Microscopy

HeLa or U2OS cells were seeded onto glass coverslips in a 12‐well plate. Upon reaching 80 % confluency, the cells were treated with either ISCH (0.1 µm) or RICO‐ISCH‐1 (0.1 µm) for 48 h. Following treatment, the cells were washed three times with PBS and fixed with 4 % PFA (500 µL) in PBS for 10 min at room temperature, followed by three washes with ice‐cold PBS. The cells were then permeabilized with 0.2 % Triton X‐100 in PBS for 10 min at room temperature, followed by three washes with PBS. Blocking was performed using 500 µL of blocking buffer composed of 1 % BSA and 22.52 mg mL^−1^ glycine in PBST (PBS + 0.1 % Tween 20) for 1 h at room temperature. The cells were then incubated with Rabbit Anti‐PML Monoclonal primary antibody (Cell Signaling, Cat. CST‐69789, 1:500 dilution in 1 % BSA in PBST) for 1 h at room temperature, followed by an overnight incubation at 4 °C. The next day, the cells were washed three times with PBS for 5 min each and incubated with the secondary antibody Goat Anti‐Rabbit IgG H&L Alexa Fluor 488 (Abcam, Cat. AB‐ab150077, 1:1000 dilution in 1% BSA in PBS) for 1 h at room temperature. The cells were then washed three times with PBS for 5 min each, followed by mounting with Prolong Gold Antifade Reagent with DAPI (Cell Signaling, Cat. 8961S) and sealing the coverslips with nail polish. The slides were imaged using a NIKON New AX‐R Confocal microscope at 60x magnification. Images were analyzed and processed using NIS‐Elements AR 5.2.

### Colony Formation Assay

U2OS cells were seeded in 6‐well plates at a density of 1000 cells per well and incubated for 12 h. Cells were then treated with either ISCH (1 µm) or RIBO‐ISCH‐1 (1 µm) and cultured for 21 days, with treatment refreshed every 3 days. At the end of the incubation period, cells were fixed and stained with 1% crystal violet solution. Plates were imaged to visualize positively stained colonies in each well.

### Statistical Analysis

Images were processed and analyzed using ImageJ software. Statistical analyses were conducted using GraphPad Prism 10.5.0. All results were presented as mean ± SEM. All experiments were done in triplicate unless otherwise specified. Statistical comparisons between two groups were performed using an unpaired two‐tailed Student's *t*‐test. For multiple comparisons, data were analyzed using one‐way ANOVA. Significance levels were denoted as follows: ^*^ *p* < 0.05, ^**^ *p* < 0.01, ^***^ *p* < 0.001, ^****^ *p* < 0.0001; ns indicates no significant difference.

## Conflict of Interest

R.I. Benhamou is a founder of Renasis.bio and a member of its scientific advisory board.

## Author Contributions

R.I.B. directed the study, conceived the idea, and designed experiments. E.K. designed experiments, conducted biochemical and cellular studies, D.D. synthesized compounds.

## Supporting information



Supporting Information

## Data Availability

The data that support the findings of this study are available in the supplementary material of this article.
