# Supplementary material for: RNA G‐Quadruplex RIBOTAC‐Mediated Targeted Degradation of lncRNA TERRA
Source: Adv Sci (Weinh). 2025 Oct 6;12(48):e12715. doi: 10.1002/advs.202512715 (PMC12752661; doi:10.1002/advs.202512715)
Supplement: Supplementary file 1 — Supporting Information [file ADVS-12-e12715-s001.docx]

**RNA G-Quadruplex RIBOTAC-Mediated Targeted Degradation of lncRNA TERRA**

Elias Khaskia,^[a]^ Dipak Dahatonde,^[a]^ and Raphael I. Benhamou ^[a],*^

^[a]^ The Institute for Drug Research of the School of Pharmacy, Faculty of Medicine, The Hebrew University of Jerusalem, Jerusalem, Israel

*Corresponding author: Raphael I. Benhamou, Email: [raphael.benhamou@mail.huji.ac.il](mailto:raphael.benhamou@mail.huji.ac.il)

|  | General chemistry methods and instruments | 2 |
| --- | --- | --- |
|  | Abbreviations | 3 |
|  | Synthetic procedures | 4-24 |
|  | Biological evaluation methods | 25-33 |
|  | Figures and Tables | 33-73 |
|  | References | 74 |

**Content:**

**1. General chemistry methods and instruments**

Unless otherwise stated, all reactions were performed in non-dry glassware under an air atmosphere and monitored by analytical thin-layer chromatography (TLC). TLC analysis was carried out using SiliCycle aluminum-backed silica gel plates (F-254). After elution, the plate was visualized under UV illumination at 254 nm or, whenever necessary, stained with Iodine, ninhydrin, or KMnO_4_ solution as visualizing agents. Analytical grade solvents for column chromatography were used as received. All chemicals were purchased at the highest commercial quality and used without further purification unless otherwise stated. Compounds were purified using silica gel chromatography (Silica gel, Bio Lab, 60 Å). Another purification was conducted with an Ultimate 3000 semi-preparative HPLC instrument (Thermo Scientific), the system composed of VWD-3400rs Detector, HPG-3200BX Pump, and Fraction Collector F. The system was equipped with an HPLC Column Luna C18 250 x 21.2 mm. Purifications were conducted with a flow rate of 15 mL/min with a gradient of 10-90% MeOH or MeCN (+ 0.1% FA) in water (+ 0.1% FA) over 55 min followed by 5 min at 90% MeOH or MeCN (+ 0.1% FA). Purities of products were analyzed by analytical HPLC by Shimadzu Nexera UHPLC (LC-40). Analyses were conducted with a flow rate of 0.3 mL/min with a gradient of 2-70% MeOH in water. Matrix-assisted laser desorption ionization time-of-flight (MALDI-TOF) mass spectrometry was performed on a BRUKER Microflex LRF MALDI-TOF/TOF instrument using dihydroxy benzoic acid as a matrix. Spectra were acquired using the Bruker Daltonics Flex Control 3.4 and analyzed using Bruker Daltonics Flex Analysis 3.4. High-resolution mass spectra (HR-MS) were measured on a Sciex X500R Q-TOF instrument. ^1^H NMR was recorded on 80, 300, and 500 MHz NMR spectrometers with CDCl_3_ or DMSO-*d*^6^ as the solvent, using TMS as an internal standard (chemical shifts *δ* in ppm unit). Peak multiplicities of ^1^H-NMR signals were designated as s (singlet), brs (broad singlet), d (doublet), dd (doublet of doublet), t (triplet), q (quartet), quintet (quin), m (multiplet), etc. Coupling constants (*J*) are in Hz, and analysis was done via MestReNova 10.0 software.

**2. Abbreviations**

AcOH: Acetic acid, AcOK: potassium acetate, CDCl_3_: chloroform-*d*, CuSO₄.5H₂O: Copper(II) sulfate pentahydrate, CrO_3_: Chromium trioxide, DCM: dichloromethane, DIPEA: *N*,*N*-Diisopropylethylamine, DMF: *N*, *N*-dimethylformamide, DMSO: dimethyl sulfoxide, EDCI: 1-(3-dimethylaminopropyl)-3-ethylcarbodiimide hydrochloride, EtAOc: ethyl acetate, EtOH: ethanol, FA: formic acid, HATU: 1-[Bis(dimethylamino)methylene]-1*H*-1,2,3-triazolo[4,5-*b*]pyridinium3-oxidehexafluorophosphate, HBr: Hydrobromic acid, HCl: hydrogen chloride, HOBt: 1-hydroxybenzotriazole, HPLC: high-performance liquid chromatography, HRMS: high-resolution mass spectrometry, H_2_SO_4_: Sulfuric acid, K_2_CO_3_: potassium carbonate, K_3_PO_4_: tri potassium phosphate, LiBr: Lithium bromide, MALDI-TOF: Matrix-assisted laser desorption ionization time-of-flight, MeCN: acetonitrile, MeI: Iodomethane, MeOH: methanol, MgSO_4_: magnesium sulfate, MS: mass spectrometry, MsCl: Methane sulfonyl chloride, NaCNBH_3_: Sodium cyanoborohydride, NaH: sodium hydride, NaOMe: Sodium methoxide, NH_2_Me: Methylamine, NMR: nuclear magnetic resonance, NaN_3_: sodium azide, NaOH: sodium hydroxide, POCl_3_: Phosphoryl chloride, *p*-TsCl: 4-Toluenesulfonyl chloride, Pd(dppf)_2_Cl_2_ CH_2_Cl_2_: 1,1'-[bis(diphenylphosphino)ferrocene] dichloro palladium dichloromethane, PE: petroleum ether, THF: tetrahydrofuran, TLC: Thin layer chromatography, TEA: triethyl amine, TFA: trifluoroacetic acid, UV: Ultraviolet.

**3. Synthetic procedures:**

**The alkyne and azide intermediates used in this study:**

**3.1.** **Synthesis of alkyne and azide intermediates used in this study**

The above alkyne and azide intermediates were synthesized via the reported procedure and used as such for the reaction.^1–3^

**Final molecules synthesized in the present work:**

**RIBO-ISCH-2**

**RIBO-ISCH-1**


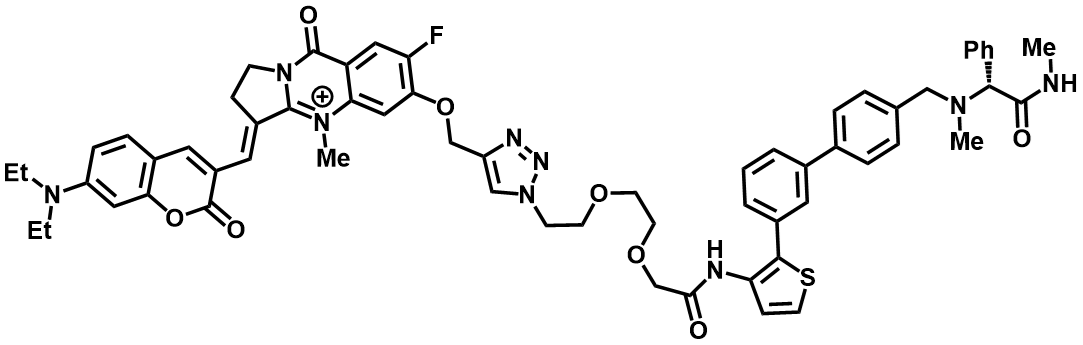


**RIBO-ISCH-3**

**RIBO-ISCH-4**

**Experimental for the synthesis of ethyl (*Z*)-5-(4-(2-(2-(2-azidoethoxy)ethoxy)ethoxy)-3-hydroxybenzylidene)-4-oxo-2-(phenylamino)-4,5-dihydrothiophene-3-carboxylate (Recruiter 1A)**

**Synthesis of Ethyl 4-oxo-2-(phenylamino)-4,5-dihydrothiophene-3-carboxylate (C-1):**

To a 50 mL clean and oven-dried round bottom flask charged ethyl 4-chloro-acetoacetate (1 g, 6.08 mmol) and anhydrous 1,4-dioxane (10 mL) were added. To this solution, portionwise added sodium hydride (0.24 g, 6.08 mmol) at 30 °C, and stirring was continued for another 20 minutes. Then, phenyl isothiocyanate solution in dioxane (10 mL) was added dropwise at 25-30 °C. After completion of the addition, the reaction mixture was warmed to 40 °C and allowed to stir for 1 h. After completion, as checked by TLC, the reaction mixture was cooled to room temperature, and then water was poured (40 mL). The solid material was filtered, wased with water (2 x 4 mL), dried under vacuum and triturated with EtOH to afford a off white solid (**C-1**, 0.95 g, 60%), which was used as such for next reaction without further purification.^1^H NMR (300 MHz, CDCl_3_): *δ* (ppm) = 7.51 – 7.39 (m, 2H), 7.36 (d, *J* = 7.8 Hz, 3H), 4.37 (q, *J* = 7.2 Hz, 2H), 3.64 (s, 2H), 1.40 (t, *J* = 7.1 Hz, 3H). MALDI TOF; calculated MS: 264.07 [M+H]^+^, measured MS: 264.18.

**Reagents and reaction conditions**: a) i. Pyridine, THF, 0 °C-rt, 2.5 h ii. NaN_3_, EtOH, 80 °C, 12 h; b) MsCl, TEA, DCM, 0 °C-rt 2 h; c) LiBr, THF, 45 °C, 12 h; d) K2CO3, DMF, 50 °C, 12 h; e) Piperidine, EtOH, 80 °C, 6 h.

**Synthesis of 2-(2-(2-azidoethoxy)ethoxy)ethan-1-ol (C-2):**

A 100 mL clean and oven-dried round-bottom flask was charged with tri-ethylene glycol (5 g, 33.29 mmol), pyridine (5.37 mL, 66.59 mmol ), and anhydrous THF (30 mL) at room temperature. The resulting reaction mixture was cooled to 0 °C and added portionwise Tosyl chloride (6.35 g, 33.29 mmol) over 10 minutes. Then the reaction mixture was allowed to stir at room temperature for 2.5 h. The reaction progress was monitored using TLC. The reaction mixture was concentrated under vacuum, and the residue was dissolved in DCM (30 mL), washed with 1M NaOH (30 mL), 1M HCl (35 mL), and brine (20 mL). Then the organic layer was dried over anhydrous MgSO_4_ and concentrated under reduced pressure to afford a yellow coloured oil. The yellow oil was dissolved in EtOH (80 mL), and sodium azide (6 g, 92.3 mmol) was added at room temperature. Then the reaction mixture was allowed to stir at 80 °C for 12 h. After completion, the reaction mixture was concentrated under reduced pressure. The residue was dissolved in DCM (30 mL), washed with 1M NaOH (40 mL), dried over anhydrous MgSO_4,_ and concentrated under reduced pressure to afford a yellow oil. The crude product was purified by using silica gel column chromatography (10-30% EtOAc in PE) to afford 2-(2-(2-azidoethoxy)ethoxy)ethan-1-ol as a yellow oil (**C-2**, 4.3 g, 38%).

**Synthesis of 1-azido-2-(2-(2-bromoethoxy)ethoxy)ethane (C-3):**

A 50 mL clean and oven-dried round-bottom flask was charged with 2-(2-(2-azidoethoxy)ethoxy)ethan-1-ol (**C-2**, 1.0 g, 5.71 mmol), TEA (0.8 mL, 5.71 mmol), and anhydrous DCM (10 mL) at room temperature. Then, mesyl chloride (0.65 g, 5.71 mmol) was added dropwise at 0 °C with constant stirring. Further, the reaction mixture was allowed to stir at room temperature for 2 h. After completion, the reaction mixture was diluted with DCM (20 mL) and washed with water (2 x 20 mL), the organic layer was dried over anhydrous MgSO_4,_ and concentrated under reduced pressure. The crude product was purified by silica gel column chromatography in PE: EtOAc (8:2, v/v) as an eluent to afford a yellow oil (0.890 g). THF (8 mL) and LiBr (0.30 g, 3.51 mmol) were added to this yellow oil at room temperature. The resulting reaction mixture was allowed to stir at 45 °C for 12 h. After completion, the reaction mixture was concentrated, directly adsorbed on to silica gel and purified by using column chromatography in PE: EtOAc (9:1, v/v) to afford a yellow oil (**C-3**, 0.43, 51%).^1^H NMR (300 MHz, CDCl_3_): *δ* (ppm) = 3.83 (t, *J* = 6.3 Hz, 2H), 3.74 – 3.65 (m, 6H), 3.49 (t, *J* = 6.2 Hz, 2H), 3.40 (t, *J* = 5.1 Hz, 2H).

**Synthesis of 4-(2-(2-(2-azidoethoxy)ethoxy)ethoxy)-3-hydroxybenzaldehyde (C-4):**

To a solution of 3,4-dihydroxy benzaldehyde (0.25 g, 1.81 mmol) and K_2_CO_3_ (0.27 g, 1.99 mmol) in DMF (3 mL) a solution of 1-azido-2-(2-(2-bromoethoxy)ethoxy)ethane (**C-3**, 0.43 g1.81 mmol) in DMF (1 mL) was added dropwise at room temperature. Then the resulting reaction mixture was allowed to stir at 50 °C for 12 h. The reaction progress was monitored using TLC. After completion, the reaction mixture was diluted with chilled water (30 mL), extracted with EtOAc (3 x 30 mL), and the combined organic layers were dried over anhydrous MgSO_4_, then concentrated under reduced pressure. The crude product was purified by silica gel chromatography in DCM: MeOH (95:5, v/v) as an eluent to afford a yellow oil (**C-4**, 0.25 g, 49%). ^1^H NMR (300 MHz, CDCl_3_): *δ* (ppm) = 9.85 (s, 1H), 7.44 (d, *J* = 2.0 Hz, 1H), 7.40 (dd, *J*_1_= 8.2, *J*_2_ = 2.0 Hz, 1H), 7.00 (d, *J* = 8.2 Hz, 1H), 4.31 – 4.22 (m, 2H), 3.98 – 3.87 (m, 2H), 3.79 – 3.54 (m, 7H), 3.41 (t, *J* = 5.0 Hz, 2H). ^13^C NMR (126 MHz, CDCl3) δ 191.08, 151.19, 147.20, 131.39, 123.84, 115.14, 113.10, 77.28, 77.02, 76.77, 70.80, 70.68, 70.15, 69.36, 69.30, 50.69.

**Synthesis of ethyl (*Z*)-5-(4-(2-(2-(2-azidoethoxy)ethoxy)ethoxy)-3-hydroxybenzylidene)-4-oxo-2-(phenylamino)-4,5-dihydrothiophene-3-carboxylate (Recruiter 1A):**

A solution of compound **C-4** (70 mg, 0.237 mmol), compound **C-1** (62.4 mg, 0.237 mmol), and piperidine (20 mg, 0.237 mmol) in EtOH (4 mL) was heated at 80 °C and allowed to stir for 4 h. The reaction progress was monitored using TLC. After complete consumption of the starting materials, the solvent was evaporated under reduced pressure. The crude product was triturated with the mixture of solvent PE: EtOAc (80:20), filtered, and dried under vacuum to afford a yellow solid (**Recruiter 1A**, 77 mg, , 60%). ^1^H NMR (300 MHz, DMSO-*d*_6_): *δ* (ppm) = 11.21 (brs, 1H), 9.42 (s, 1H), 7.60 – 7.37 (m, 6H), 7.07 – 6.91 (m, 3H), 4.27 (q, *J* = 7.1 Hz, 2H), 4.11 (t, *J* = 4.6 Hz, 2H), 3.74 (t, *J* = 4.6 Hz, 2H), 3.58 (m, 6H), 3.37 (t, *J* = 4.9 Hz, 2H), 1.28 (t, *J* = 7.1 Hz, 3H).

**Experimental for the synthesis of Ethyl (*Z*)-5-(4-(2-azidoethoxy)-3-hydroxybenzylidene)-4-oxo-2-(phenylamino)-4,5-dihydrothiophene-3-carboxylate (Recruiter 1B):**

**Reagents and reaction conditions**: a) NaN_3_, acetone, 60 °C, 10 h; b) *p*-TsCl, pyridine, THF, 25 °C, 2 h; c) K_2_CO_3_, DMF, 50 °C, 12 h; d) piperidine, EtOH, 80 °C, 6 h.

**Synthesis of 2-azidoethan-1-ol (C-5):**


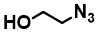
To a 100 mL clean round-bottom flask, 2-bromoethanol (2 g, 8.0 mmol), sodium azide (1.56 g, 9.60 mmol), and a mixture of acetone: water (2:1, 40 mL) were added. The resulting reaction mixture was allowed to stir at 60 °C for 12 h. After completion, as checked by TLC, the reaction mixture was extracted with diethyl ether (4 x 30 mL), organic layers were combined, dried over anhydrous MgSO_4,_ and concentrated under reduced pressure to afford a colourless oil (**C-5**, 0.95 g, 68%).

**Synthesis of 2-azidoethyl 4-methylbenzenesulfonate (C-6):**

2-azidoethan-1-ol (**C-5**) (0.95 g, 10.91 mmol) was added to a 50 mL clean and dry round-bottom flask, then THF (10 mL), and pyridine (1.1 mL, 13.09 mmol) were charged. The reaction mixture was cooled down to 0 °C, and slowly added *p*-TsCl (2.4 g, 12.00 mmol). Further, the reaction mixture was allowed to stir at room temperature for 2 h. After completion, THF was evaporated under reduced pressure, the solution was extracted with DCM (3 x 30 mL) and washed with brine. The combined organic layers were dried over anhydrous MgSO_4_ and evaporated to afford a yellow oil (**C-6**, 0.95 g, 68%).

**Synthesis of 4-(2-azidoethoxy)-3-hydroxybenzaldehyde (C-7):**

A solution of 3,4-dihydroxybenzaldehyde (0.5 g, 3.62 mmol), 2-azidoethyl 4-methylbenzenesulfonate (**C-6**, 0.87 g, 3.62 mmol) and K_2_CO_3_ (0.5 g, 3.62 mmol) in DMF (3 mL) was heated at 50 °C for 12 h. After completion, the reaction mixture was diluted with water (30 mL), extracted by using EtOAc (3 x 30 mL), and the combined organic layers were dried over anhydrous MgSO_4_ and concentrated under reduced pressure. The crude product was purified by silica gel chromatography, PE: EtOAc (80:20, v/v) as an eluent to afford an off-yellow solid (**C-7**, 0.20 g, 26%). ^1^H NMR (500 MHz, CDCl_3_): *δ* (ppm) = 9.86 (s, 1H), 7.48 (d, *J* = 2.0 Hz, 1H), 6.97 (d, *J* = 8.2 Hz, 1H), 4.32 (dd, *J*_1_ = 5.4, *J*_2_ = 4.6 Hz, 2H), 3.72 (t, *J* = 5.0 Hz, 2H). ^13^C NMR (126 MHz, CDCl3) δ 190.91, 150.28, 146.30, 131.41, 124.06, 115.11, 111.48, 77.28, 77.03, 76.77, 68.20, 50.07.

**Synthesis of Ethyl (*Z*)-5-(4-(2-azidoethoxy)-3-hydroxybenzylidene)-4-oxo-2-(phenylamino)-4,5-dihydrothiophene-3-carboxylate (Recruiter 1B):**

A 50 mL clean and dry round bottom flask attached with a reflux condenser was charged with compound **C-7** (50 mg, 0.241 mmol), compound **C-1** (63.5 mg, 0.241 mmol), piperidine (20.6 mg, 0.241 mmol), and EtOH (10 mL). The resulting reaction mixture was heated at 80 ^°^C and allowed to stir for 4 h. The reaction progress was monitored using TLC. After completion, the reaction mixture was cooled to room temperature. The solid material was filtered and dried under vacuum to afford a yellow solid (**Recruiter 1B**, 60 mg, 55%). ^1^H NMR (300 MHz, DMSO-*d*_6_): *δ* (ppm) = 11.24 (s, 1H), 9.50 (s, 1H), 7.62 – 7.34 (m, 6H), 7.10 – 6.92 (m, 3H), 4.27 (q, *J* = 7.1 Hz, 2H), 4.16 (t, *J* = 4.8 Hz, 2H), 3.65 (t, *J* = 4.8 Hz, 2H), 1.29 (t, *J* = 7.1 Hz, 3H). MALDI-TOF; calculated MS: 452.48 [M+H]^+^, Observed MS: 453.47.

**Synthesis of 3-Azidopropanoic acid** (**C-8**):

To a 50 mL clean and dried round-bottom flask was added 3-bromopropionic acid (1 g, 6.54 mmol), sodium azide (0.89 g, 13.07 mmol), and acetonitrile (20 mL). The reaction mixture was allowed to stir at 70 °C for 12 h. After completion, the reaction mixture was cooled to room temperature and acidified to pH = 1 by using concentrated HCl and extracted with diethyl ether (3 x 30 mL). The combined organic layers were washed with brine (40 mL), dried over anhydrous MgSO_4_, and concentrated under vacuum at below 25 °C to afford 3-azidopropanoic acid as a light yellow oil (**C-8**, 0.52 g, 69%).

**Synthesis of 2-(2-(2-azidoethoxy)ethoxy)acetic acid (C-9):**

The compound **C-3** (1.0 g, 5.71 mmol) was dissolved in acetone (60 mL), cooled to 4 °C, then dropwise added freshly prepared Jones reacgent (6 mL, Jones reagent prepared by dissolving 3 g of CrO_3_ in 6 mL of distiiled water, 2.8 mL conc. H_2_SO_4_ and adjusted the final valume of solution to 10 ml by adding distilled water). After the addition, the resulting reaction mixture was allowed to stir at room temperature for 1 h. The reaction progress was monitored using TLC. After complete consumption of the starting material, the reaction mixture was quenched by the addition of propan-2-ol (5 mL). After 20 minutes, acetone (60 mL) was added, and the green precipitate was filtered with Celite 545. The filtrate was concentrated and adsorbed onto silica gel and purified by silica gel column chromatography using DCM: MeOH (96:4, v/v) as an eluent to afford a yellow oil (**C-9**, 0.14 g, 27%).

**Experimental and spectroscopic data for the synthesis of Recruiter 2A and Recruiter 2B:**

Compounds **C-10**, **C-11**, **C-12**, and **C-13** were synthesized according to previously reported methods.^1^

**Reagents and reaction conditions**: a) AcOK, Pd(dppf)Cl_2_, 1,4-dioxane, 90 °C, 12 h; b) K_3_PO_4_, 1,4-dioxane: H_2_O, 90 °C, 2 h; c) NaCNBH_3_, AcOH, MeOH, rt, 12 h; d) NH_2_Me, HATU, DIPEA, DMF, rt, 12 h; e) TFA, DCM, rt, 2 h; f) EDCI, HOBt, DMF, rt, 12 h.

**Synthesis of *Tert*-butyl (2-(4'-formyl-[1,1'-biphenyl]-3-yl)thiophen-3-yl)carbamate (C-10):**

To a 50 mL clean and dry round bottom flask attached with reflux condenser, 3'-bromo-[1,1'-biphenyl]-4-carbaldehyde (0.5 g, 1.91 mmol), bis(pinacolato)diboron (0.729 g, 2.87 mmol) and potassium acetate (0.281 g, 2.87 mmol) were dissolved in anhydrous 1,4-dioxane (10 mL). Then the reaction mixture was purged with argon, and a catalyst 1,1’[bis(diphenylphosphino)ferrocene]dichloropalladium dichloromethane adduct (0.15 g, 0.91 mmol) was charged once. Then the reaction mixture was allowed to stir at 90 °C for 12 h. The reaction progress was monitored using TLC. After complete consumption of starting material, *tert*-butyl(2-bromothiophen-3-yl)carbamate (0.523 g, 1.91 mmol) and tri-potassium phosphate (1.21 g, 5.74 mmol) as a water solution (7 mL) were added, and the reaction mixture was allowed to stir further at 90 °C for 2 h. After completion, the reaction mixture was filtered, and the filtrate was concentrated under vacuum. The crude product was directly adsorbed onto silica gel and separated using column chromatography PE: EtOAc (1-20%) to give a white solid as the desired compound (**C-10**, 0.4 g, 55%). 1H NMR (300 MHz, CDCl_3_) δ 10.08 (s, 1H), 8.01 – 7.94 (m, 2H), 7.78 (d, J = 8.2 Hz, 2H), 7.72 (d, J = 1.7 Hz, 1H), 7.65 – 7.49 (m, 4H), 7.28 (d, J = 5.5 Hz, 1H), 6.60 (s, 1H), 1.50 (s, 10H).

**Synthesis of (*S*)-2-(((3'-(3-((*Tert*-butoxycarbonyl)amino)thiophen-2-yl)-[1,1'-biphenyl]-4-yl)methyl)(methyl)amino)-2-phenylacetic acid (C-11):**

To a stirred solution of compound **C-10** (0.4 g, 1.05 mmol) in MeOH (10 mL), *N*-methyl-L-phenylglycine (0.26 g, 1.57 mmol) and acetic acid (0.34 mL) were added. Then the sodium cyanoborohydride (0.198 g, 3.15 mmol) was added portion-wise, and the resulting reaction mixture was allowed to stir for 12 h at room temperature. As monitored by TLC, on completion, the excess reducing agents were quenched by adding a few drops of water. The reaction mixture was concentrated, and the crude product was purified by silica gel column chromatography using DCM: MeOH (98:2) as eluent to afford a pale yellow solid (**C-11**, 0.35 g, 64%). MALDI-TOF; calculated MS: 528.6 [M+H]^+^, Observed MS: 529.50.

**Synthesis of *Tert*-butyl (*S*)-(2-(4'-((methyl(2-(methylamino)-2-oxo-1-phenylethyl)amino)methyl)-[1,1'-biphenyl]-3-yl)thiophen-3-yl)carbamate (C-12):**

A 50 mL round-bottom flask fitted with a drying tube was added compound **C-11** (0.35 g, 0.67 mmol) in anhydrous DMF. Then, with continuous stirring at room temperature, HATU (0.38 g, 0.99 mmol) was added, followed by DIPEA (0.57 mL, 3.36 mmol). Subsequently, the reaction mixture was allowed to stir at room temperature for 30 minutes. Then N-methylamine in 2M THF solution (0.67 mL, 1.34 mmol) was added, and the reaction mixture was stirred for 12 h at room temperature. After completion, the reaction mixture was diluted with H_2_O (20 mL) and extracted with EtOAc (4 X 20 mL). The combined layer was dried over anhydrous MgSO_4_ and evaporated under reduced pressure to afford a crude residue that was purified by column chromatography over silica gel using DCM: MeOH (95:5, v/v) as eluent to afford a pale yellow solid (**C-12**, 0.24 g, 66%). MALDI-TOF; calculated MS: 541.7 [M+Na]^+^, Observed MS: 542.5.

**Synthesis of (*S*)-2-(((3'-(3-Aminothiophen-2-yl)-[1,1'-biphenyl]-4-yl)methyl)(methyl)amino)-*N*-methyl-2-phenylacetamide (C-13):**

Compound **C-12** (0.2 g, 0.369 mmol) was dissolved in DCM (6 mL). To this reaction mixture, TFA (2 mL) was added dropwise at room temperature. The reaction mixture was allowed to stir at room temperature for 2 h. After complete consumption of the starting material, the reaction mixture was concentrated under vacuum. The residue was dissolved in water (20 mL) and neutralized by using a saturated solution of Na_2_CO_3_. The product was extracted by using DCM (3 x 20 mL). The combined organic layers were dried over anhydrous MgSO_4_ and concentrated under reduced pressure to afford a crude product that was purified by silica gel column chromatography using DCM: MeOH (90:10, v/v) as an eluent to afford a pale yellow solid (**C-13**, 120 mg, 73%). ^1^H NMR (500 MHz, CDCl_3_) *δ* (ppm) = 7.73 (d, *J* = 1.9 Hz, 1H), 7.72 – 7.68 (m, 2H), 7.65 – 7.62 (m, 2H), 7.55 – 7.45 (m, 9H), 7.19 (d, *J* = 5.4 Hz, 1H), 6.79 (d, *J* = 5.3 Hz, 1H), 5.53 (s, 1H), 4.55 (d, *J* = 12.8 Hz, 1H), 4.28 (d, *J* = 12.9 Hz, 1H), 2.81 (d, *J* = 4.7 Hz, 3H), 2.70 (s, 3H). MALDI-TOF; calculated MS: 442.2 [M+H]^+^, observed MS: 442.1.

**Synthesis of (*S*)-2-(((3'-(3-(2-(2-(2-azidoethoxy)ethoxy)acetamido)thiophen-2-yl)-[1,1'-biphenyl]-4-yl)methyl)(methyl)amino)-*N*-methyl-2-phenylacetamide (Recruiter 2A):**

To a 25 mL dry round bottom flask, 2-(2-(2-azidoethoxy)ethoxy)acetic acid **C-9** (25 mg, 132.2 µmol) was added with anhydrous DMF. Then, 1-hydroxybenztriazole (58.4 mg, 132 µmol) was added, and after 5 minutes, 1-(3-dimethylaminopropyl)-3-ethylcarbodiimide hydrochloride (27 mg, 173.8 µmol) was added with constant stirring, under an argon atmosphere. The resulting reaction mixture was allowed to stir at room temperature for 20 minutes. Then amine **C-13** (58 mg, 132 µmol**)** was added, and the reaction mixture was stirred for 12 h. After completion, the reaction mixture was diluted with water (20 mL), and the aqueous layer was extracted with EtOAc (3 x 20 mL). The organic layers were combined, dried over anhydrous MgSO_4_, and concentrated under reduced pressure. The crude product was purified by silica gel chromatography with DCM: MeOH (97:3, v/v) as an eluent to yield **Recruiter 2A** (34 mg, yield 34%). MALDI-TOF; calculated MS: 612.7 [M+H]^+^, observed MS: 613.2. This compound was directly used for the next step without further charectrization.

**Synthesis of (*S*)-3-azido-*N*-(2-(4'-((methyl(2-(methylamino)-2-oxo-1-phenylethyl)amino)methyl)-[1,1'-biphenyl]-3-yl)thiophen-3-yl)propanamide (Recruiter 2B):**

To a 25 mL dry round-bottom flask, 3-azidopropanoic acid **C-8** (20 mg, 173.8 µmol) was dissolved in anhydrous DMF. To this solution, 1-hydroxybenztriazole (61.4 mg, 173.8 µmol) was added under an argon atmosphere, and after 5 minutes, 1-(3-dimethylaminopropyl)-3-ethylcarbodiimide hydrochloride (27 mg, 173.8 µmol) was added. The resulting reaction mixture was allowed to stir at room temperature for 20 minutes. Then, amine **C-13** (61 mg, 139.0 µmol) was added, and the reaction mixture was allowed to stir at room temperature for 12 h. After completion, the reaction mixture was diluted with water (20 mL), and the aqueous layer was extracted with EtOAc (3 x 20 mL). The organic layers were combined, dried over anhydrous MgSO_4_, and concentrated under reduced pressure. The crude product was purified by silica gel chromatography with DCM: MeOH (97:3, v/v) as an eluent to yield **Recruiter** **2B** (48 mg, 51%). MALDI-TOF; calculated MS: 538.6 [M+H]^+^, observed MS: 539.2. This compound was directly used for the next step without further charectrization.

**Experimental and spectroscopic data for the synthesis of 7-(Diethylamino)-2-oxo-2*H*-chromene-3-carbaldehyde (C-15)**

Compounds **C-14** and **C-15** were synthesized according to previously reported methods.^4^

To a 50 mL round-bottom flask, 4-(diethylamino)-2-hydroxybenzaldehyde (1 g, 5.17 mmol), dimethyl malonate (1.37 g, 10.35 mmol), piperidine (0.44 g, 5.17 mmol), and EtOH (20 mL) were added. The resulting mixture was refluxed for 12 h. The reaction progress was monitored using TLC. After completion of the reaction, the EtOH was evaporated under reduced pressure. Subsequently, a mixture of HCl: AcOH (1:1, 20 mL) was added to the reaction and allowed to stir at reflux conditions for 24 hours. After completion, the reaction mixture was cooled to room temperature and poured onto ice-cold water (40 mL). Brown precipitate formed after adjusting the pH up to 5 by using a 40% NaOH solution. Subsequently, the solid was filtered, washed with water four times, and dried under reduced pressure. Compound **C-14** was obtained as a yellowish brown solid (0.8 g, 71% yield). The compound is used as such for the next stage without further purification.

To a 50 mL clean and oven-dried round-bottom flask, anhydrous DMF (1.78 mL) was added and phosphoryl oxychloride (POCl_3,_ 0.71 mL, 7.55 mmol) was added dropwise at 0 °C under a nitrogen atmosphere. The reaction mixture was allowed to stir at 0 °C for 30 minutes to afford pale red material. Then, compound **C-14** (1.0 g, 4.60 mmol) dissolved in anhydrous DMF (2 mL) was added dropwise to the reaction mixture over 30 min at 0 °C. Subsequently, the reaction mixture was warmed to room temperature and allowed to stir for 30 minutes, followed by heating at 60 °C and stirred for 1 h. The reaction progress was monitored using TLC. After completion, the reaction mixture was slowly added into ice-cold saturated Na_2_CO_3_ (40 mL) solution, and the resulting yellow solid precipitate was allowed to stir at room temperature for 30 minutes. The solid precipitate was filtered, washed with water (2x 5 mL), and dried under reduced pressure. The precipitate (**C-15**, 0.81 g, 71%) had enough purity to use as such for the next reaction.

**Experimental and spectroscopic data for the synthesis of ISCH:**

Compounds **C-16, C-17, C-18, C-19, C-20** and **ISCH** were synthesized according to previously reported methods.^3^

**Reagents and reaction conditions**: a) POCl_3_, 105 °C, 12 h; b) NaOMe, MeOH, 60 °C, 18 h; c) AcOH: HBr (1:1), 140 °C, 48 h; d) Propargyl Bromide, K_2_CO_3_, acetone, 50 °C, 15 h; e) MeI, MeCN, 50 °C, 24 h; f) Cat. Piperidine, EtOH, 50 °C, 12 h.

**Synthesis of 6,7-Difluoro-2,3-dihydropyrrolo[2,1-*b*]quinazolin-9(1*H*)-one (C-16):**

A 50 mL clean and oven-dried round-bottom flask attached with a reflux condenser was charged with 2-amino-4,5-difluoro-benzoic acid (1 g, 5.78 mmol), 2-pyrrolidone (1.1 mL, 14.44 mmol). POCl_3_ (4.32 mL, 46.21 mmol) was added dropwise for 30 minutes at 0 °C. Further, the reaction mixture was allowed to stir at 105 °C for 12 h. Upon completion, the reaction mixture was quenched by slowly adding ice-cold water, and the pH was adjusted to 9 using concentrated NaOH solution. The solid material formed was filtered and dried under reduced pressure. The crude product was purified by using column chromatography PE: EtOAc (1:1 v/v), to afford a white solid (**C-16**, 1.04 g, 81%).

**Synthesis of 7-Fluoro-6-methoxy-2,3-dihydropyrrolo[2,1-*b*]quinazolin-9(1*H*)-one** (**C-17**):

A 50 mL round bottom flask was charged with compound **C-16** (1 g, 4.50 mmol), sodium methoxide (0.97 g, 18.00 mmol), and MeOH (25 mL). Then the reaction mixture was allowed to stir at 60 °C for 18 h. The reaction progress was monitored using TLC. After completion, the reaction mass was filtered, and the filtrate was evaporated to dryness. The crude product was triturated with water, the solid was filtered, washed with water (2x 5 mL), and dried under reduced pressure to afford a pale purple solid (**C-17**, 0.85 g, 75%). MALDI TOF; calculated MS: 234.230 [M+H]^+^, observed MS: 235.264.

**Synthesis of 7-Fluoro-6-hydroxy-2,3-dihydropyrrolo[2,1-*b*]quinazolin-9(1*H*)-one** (**C-18**):

A 50 mL round-bottom flask was charged with compound **C-17** (0.8 g, 3.42 mmol) in a mixture of acids AcOH: HBr (10 mL, 1:1). Then, the reaction mixture was heated to reflux at 140 °C for 48 h. The reaction progress was monitored using TLC. After completion, the reaction mass was filtered, and the filtrate was evaporated to dryness. The residue was neutralized to pH 5 using concentrated NaOH solution. The solid material was filtered and dried under reduced pressure. The crude product was purified using column chromatography DCM: MeOH (95:5, v/v), to afford a white solid (**C-18**, 0.258 g, 34%).

**Synthesis of 7-Fluoro-6-(prop-2-yn-1-yloxy)-2,3-dihydropyrrolo[2,1-*b*]quinazolin-9(1*H*)-one (C-19)**:

To a 50 mL clean and oven-dried round-bottom flask, compound **C-18** (0.25 g, 1.14 mmol), anhydrous K_2_CO_3_ (0.19 g, 1.43 mmol), and acetone (10 mL) were added. Then, propargyl bromide (0.15 g, 1.26 mmol) dissolved in acetone (2 mL) was added dropwise at room temperature over 5 minutes. Then, the resulting reaction mixture was allowed to stir at 50 °C for 15 h. The reaction progress was monitored using TLC. Upon completion, the reaction mixture was filtered, and the filtrate was concentrated under reduced pressure. Then, the crude product was dissolved in water (10 mL) and extracted with EtOAc (3 x 15 mL). The organic layers were combined, dried over anhydrous Na_2_SO_4,_ and concentrated to afford a white solid (**C-19**, 0.213 g, 72%).

**Synthesis of 7-Fluoro-4-methyl-9-oxo-6-(prop-2-yn-1-yloxy)-1,2,3,9-tetrahydropyrrolo[2,1-*b*]quinazolin-4-ium iodide (C-20)**:

A 25 mL clean and oven-dried round-bottom flask was charged with compound **C-19** (0.166 g, 0.642 mmol), MeI (0.73 g, 5.14 mmol), and MeCN (2 mL). The reaction mixture was allowed to stir at 60 °C for 24 h. The reaction progress was monitored using TLC. After cooling, the reaction mass was filtered, washed with anhydrous diethyl ether, and dried under reduced pressure to afford a white solid (**C-20**, 0.149 g, 84%), which was used as such without further purification for the next reaction. MALDI TOF; calculated MS: 273.287 [M-I]^+^, observed MS: 273.231.

**Synthesis of ISCH:**

****To a 25 mL clean and dry round bottom flask, compound **C-20** (0.4 g, 1 mmol), compound **C-15** (0.29 g, 1.20 mmol), a catalytic amount of piperidine, and EtOH (10 mL) were added. The reaction mixture was allowed to stir at 80 °C for 12 h. After cooling the reaction mixture to room temperature, the solvent was evaporated under reduced pressure. The crude product was directly adsorbed onto silica gel, purified by using column chromatography with DCM: MeOH (50:1, v/v) as an eluent to afford a brownish black solid (**ISCH**, 0.40 g, yield 59%). Purity: 98.9% by HPLC, MALDI TOF; calculated MS: 500.550 [M-I]^+^, observed MS: 500.612.

**Experimental and spectroscopic data for the synthesis of RIBO-ISCH-1 and RIBO-ISCH-2**

**Synthesis of RIBO-ISCH-1:**

To a stirred solution of (*E*)-3-((7-(diethylamino)-2-oxo-2*H*-chromen-3-yl)methylene)-7-fluoro-4-methyl-9-oxo-6-(prop-2-yn-1-yloxy)-1,2,3,9-tetrahydropyrrolo[2,1-*b*]quinazolin-4-ium iodide (**ISCH**, 15 mg, 23.91 µmol) and ethyl (*Z*)-5-(4-(2-(2-(2-azidoethoxy)ethoxy)ethoxy)-3-hydroxybenzylidene)-4-oxo-2-(phenylamino)-4,5-dihydrothiophene-3-carboxylate (**Recruiter 1A**, 12.92 mg, 23.91 µmol) in a mixture of DMF: H_2_O (1.5:0.5 mL) was added CuSO₄.5H₂O (1.0 mg, 2.39 µmol) and Sodium Ascorbate (1.0 mg, 4.78 µmol) at 60 °C for 12 h. After checking for completion of the reaction using TLC, the solvent was removed under reduced pressure, and the crude was purified by silica gel column chromatography DCM: MeOH (2-3%, v/v) and then was purified using UV-HPLC (10 to 90% MeOH in H_2_O + 0.1% FA, 50 min gradient) to afford a product (*E*)-3-((7-(diethylamino)-2-oxo-2*H*-chromen-3-yl)methylene)-6-((1-(2-(2-(2-(4-((*Z*)-(4-(ethoxycarbonyl)-3-oxo-5-(phenylamino)thiophen-2(3*H*)-ylidene)methyl)-2-hydroxyphenoxy)ethoxy)ethoxy)ethyl)-1*H*-1,2,3-triazol-4-yl)methoxy)-7-fluoro-4-methyl-9-oxo-1,2,3,9-tetrahydropyrrolo[2,1-*b*]quinazolin-4-ium iodide (**RIBO-ISCH-1**, 5.0 mg, 18% yield) as a blackish red solid. Purity by HPLC: 98% HR-MS (ESI); calculated MS: 1040.3659 [M-I]^+^, measured MS: 1040.3633. ^1^H NMR (500 MHz, CDCl_3_) δ 7.89 (s, 0H), 7.69 (d, J = 6.8 Hz, 1H), 7.59 (t, J = 2.8 Hz, 0H), 7.53 (d, J = 8.6 Hz, 0H), 7.47 (ddt, J = 8.4, 6.0, 2.9 Hz, 1H), 7.40 – 7.36 (m, 1H), 7.35 (d, J = 1.6 Hz, 1H), 7.29 (dd, J = 9.0, 3.1 Hz, 0H), 7.16 (d, J = 12.4 Hz, 0H), 7.12 (dd, J = 8.6, 2.5 Hz, 0H), 7.08 (t, J = 2.5 Hz, 0H), 6.99 (dd, J = 8.4, 2.3 Hz, 0H), 6.89 (d, J = 8.3 Hz, 0H), 6.65 – 6.55 (m, 0H), 6.48 (d, J = 2.5 Hz, 0H), 6.30 (dd, J = 13.8, 7.1 Hz, 0H), 4.56 (t, J = 4.9 Hz, 1H), 4.40 (q, J = 7.1 Hz, 1H), 4.23 – 4.08 (m, 1H), 3.91 (q, J = 5.8, 4.9 Hz, 2H), 3.71 – 3.66 (m, 1H), 3.66 – 3.62 (m, 1H), 3.60 (s, 2H), 3.44 (q, J = 7.0 Hz, 2H), 3.13 – 3.03 (m, 1H), 2.83 (d, J = 4.3 Hz, 1H), 1.46 – 1.40 (m, 2H), 0.86 – 0.81 (m, 4H). ^13^C NMR (126 MHz, CDCl_3_) δ 129.89, 128.35, 124.65, 124.46, 123.96, 123.72, 119.10, 116.27, 109.37, 97.20, 96.03, 77.22, 70.58, 70.46, 69.35, 69.27, 62.79, 50.50, 45.02, 43.66, 33.71, 31.94, 31.45, 30.20, 30.05, 29.94, 29.71, 29.67, 29.37, 26.71, 24.57, 22.70, 14.47, 14.13, 12.49, 1.03.

**Synthesis of RIBO-ISCH-2**

To a stirred solution of (*E*)-3-((7-(diethylamino)-2-oxo-2*H*-chromen-3-yl)methylene)-7-fluoro-4-methyl-9-oxo-6-(prop-2-yn-1-yloxy)-1,2,3,9-tetrahydropyrrolo[2,1-*b*]quinazolin-4-ium iodide (**ISCH**, 15 mg, 23.91 µmol) and ethyl (*Z*)-5-(4-(2-azidoethoxy)-3-hydroxybenzylidene)-4-oxo-2-(phenylamino)-4,5-dihydrothiophene-3-carboxylate (**Recruiter 1B**, 12.96 mg, 28.64 µmol) in a mixture of DMF: H_2_O (1.5:0.5 mL) was added CuSO₄.5H₂O (1.0 mg, 2.39 µmol) and Sodium Ascorbate (1.0 mg, 4.78 µmol) at 60 °C for 12 h. After checking for completion of the reaction using TLC, the solvent was removed under reduced pressure, and the crude was purified by silica gel column chromatography DCM: MeOH (2-3%, v/v) and then was purified using UV-HPLC (10 to 90% MeOH in H_2_O + 0.1% FA, 50 min gradient) to afford a product **RIBO-ISCH-2** (*E*)-3-((7-(diethylamino)-2-oxo-2*H*-chromen-3-yl)methylene)-6-((1-(2-(4-((*Z*)-(4-(ethoxycarbonyl)-3-oxo-5-(phenylamino)thiophen-2(3*H*)-ylidene)methyl)-2-hydroxyphenoxy)ethyl)-1*H*-1,2,3-triazol-4-yl)methoxy)-7-fluoro-4-methyl-9-oxo-1,2,3,9-tetrahydropyrrolo[2,1-*b*]quinazolin-4-ium iodide (4.0 mg, 17% yield) as a blackish red solid. Purity by HPLC: 93% HR-MS (ESI); calculated for MS: 952.3135 [M-I]^+^, measured MS: 952.3133.

**Experimental and spectroscopic data for the synthesis of RIBO-ISCH-3 and RIBO-ISCH-4**

**Synthesis of RIBO-ISCH-3**

To a stirred solution of (*E*)-3-((7-(diethylamino)-2-oxo-2*H*-chromen-3-yl)methylene)-7-fluoro-4-methyl-9-oxo-6-(prop-2-yn-1-yloxy)-1,2,3,9-tetrahydropyrrolo[2,1-*b*]quinazolin-4-ium iodide (**ISCH**, 10 mg, 15.94 µmol) and (*S*)-2-(((3'-(3-(2-(2-(2-azidoethoxy)ethoxy)acetamido)thiophen-2-yl)-[1,1'-biphenyl]-4-yl)methyl)(methyl)amino)-*N*-methyl-2-phenylacetamide (**Recruiter 2A**, 11.72 mg, 19.12 µmol) in a mixture of DMF: H_2_O (1.5:0.5 mL) was added CuSO₄.5H₂O (0.4 mg, 1.59 µmol) and Sodium Ascorbate (0.6 mg, 3.19 µmol) at 60 °C for 12 h. After checking for completion of the reaction using TLC, the solvent was removed under reduced pressure, and the crude was purified by silica gel column chromatography DCM: MeOH (2-3%, v/v) and then was purified using UV-HPLC (10 to 90% MeOH in H_2_O + 0.1% FA, 50 min gradient) to afford a product **RIBO-ISCH-3** (*S, E*)-3-((7-(diethylamino)-2-oxo-2*H*-chromen-3-yl)methylene)-7-fluoro-4-methyl-6-((1-(2-(2-(2-((2-(4'-((methyl(2-(methylamino)-2-oxo-1-phenylethyl)amino)methyl)-[1,1'-biphenyl]-3-yl)thiophen-3-yl)amino)-2-oxoethoxy)ethoxy)ethyl)-1*H*-1,2,3-triazol-4-yl)methoxy)-9-oxo-1,2,3,9-tetrahydropyrrolo[2,1-*b*]quinazolin-4-ium iodide (6.0 mg, 30% yield) as a blackish red solid. Purity by HPLC: 96% HR-MS (ESI); calculated for MS: 1112.4499 [M-I]^+^, measured MS: 1112.4494.

**Synthesis of RIBO-ISCH-4**

To a stirred solution of (*E*)-3-((7-(diethylamino)-2-oxo-2*H*-chromen-3-yl)methylene)-7-fluoro-4-methyl-9-oxo-6-(prop-2-yn-1-yloxy)-1,2,3,9-tetrahydropyrrolo[2,1-*b*]quinazolin-4-ium iodide (**ISCH**, 12 mg, 19.12 µmol) and (*S*)-3-azido-*N*-(2-(4'-((methyl(2-(methylamino)-2-oxo-1-phenylethyl)amino)methyl)-[1,1'-biphenyl]-3-yl)thiophen-3-yl)propanamide (**Recruiter 2B**, 12.36 mg, 22.95 µmol) in a mixture of DMF: H_2_O (1.5:0.5 mL) was added CuSO₄.5H₂O (0.5 mg, 1.19 µmol) and Sodium Ascorbate (1.0 mg, 3.82 µmol) at 60 °C for 12 h. After checking for completion of the reaction using TLC, the solvent was removed under reduced pressure, and the crude was purified by silica gel column chromatography DCM: MeOH (2-3%, v/v) and then was purified using UV-HPLC (10 to 90% MeOH in H_2_O + 0.1% FA, 50 min gradient) to afford a product **RIBO-ISCH-4** (*S, E*)-3-((7-(diethylamino)-2-oxo-2*H*-chromen-3-yl)methylene)-7-fluoro-4-methyl-6-((1-(3-((2-(4'-((methyl(2-(methylamino)-2-oxo-1-phenylethyl)amino)methyl)-[1,1'-biphenyl]-3-yl)thiophen-3-yl)amino)-3-oxopropyl)-1*H*-1,2,3-triazol-4-yl)methoxy)-9-oxo-1,2,3,9-tetrahydropyrrolo[2,1-*b*]quinazolin-4-ium iodide (2.5 mg, 13% yield) as a blackish red solid. Purity by HPLC: 93% HR-MS (ESI); calculated for MS: 1038.4131 [M-I]^+^, observed MS: 520.7175 [M+2H]^+2^

**4. Biological evaluation methods:**

**Preparation of Recombinant RNase L-GST Protein**

RNase L-GST protein was purchased from Gene-Script company (Lot: U282HCNSG0-8/P2IH001), expression host: E. coli, purification: protein was obtained from the supernatant of cell lysate, GST column + Superdex 200 column, purity: ≥80 % (SDS-PAGE under reducing condition, storage: 40 mM HEPES, 140 mM NaCl, 4 mM MgCl2, 2 mM DTT, 30 % Glycerol, pH 7.4), Protein Length = 741.

MESRDHNNPQEGPTSSSGRRAAVEDNHLLIKAVQNEDVDLVQQLLEGGANVNFQEEEGGWTPLHNAVQMSREDIVELLLRHGADPVLRKKNGATPFILAAIAGSVKLLKLFLSKGADVNECDFYGFTAFMEAAVYGKVKALKFLYKRGANVNLRRKTKEDQERLRKGGATALMDAAEKGHVEVLKILLDEMGADVNACDNMGRNALIHALLSSDDSDVEAITHLLLDHGADVNVRGERGKTPLILAVEKKHLGLVQRLLEQEHIEINDTDSDGKTALLLAVELKLKKIAELLCKRGASTDCGDLVMTARRNYDHSLVKVLLSHGAKEDFHPPAEDWKPQSSHWGAALKDLHRIYRPMIGKLKFFIDEKYKIADTSEGGIYLGFYEKQEVAVKTFCEGSPRAQREVSCLQSSRENSHLVTFYGSESHRGHLFVCVTLCEQTLEACLDVHRGEDVENEEDEFARNVLSSIFKAVQELHLSCGYTHQDLQPQNILIDSKKAAHLADFDKSIKWAGDPQEVKRDLEDLGRLVLYVVKKGSISFEDLKAQSNEEVVQLSPDEETKDLIHRLFHPGEHVRDCLSDLLGHPFFWTWESRYRTLRNVGNESDIKTRKSESEILRLLQPGPSEHSKSFDKWTTKINECVMKKMNKFYEKRGNFYQNTVGDLLKFIRNLGEHIDEEKHKKMKLKIGDPSLYFQKTFPDLVIYVYTKLQNTEYRKHFPQTHSPNKPQCDGAGGASGLASPGC

**Cell Lines**

Compounds were tested in HeLa human cervical cancer cell line (AC-free, ECACC 08011102) and U2OS human osteosarcoma cell line (HTB-96; ATCC).

**Cell Culture**

HeLa cells (AC-free, ECACC 08011102) were obtained from the European Collection of Authenticated Cell Cultures and cultured in DMEM medium containing Earle's salts and L-glutamine, supplemented with 10% (v/v) fetal bovine serum (FBS; Sigma-Aldrich, F9665) and 1% penicillin/streptomycin solution (Diagnovum, D910). U2OS cells (ATCC HTB-96) were purchased from the American Type Culture Collection and maintained in McCoy's 5A medium with L-glutamine, supplemented with 10% (v/v) fetal bovine serum (FBS; Sigma-Aldrich, F9665) and 1% penicillin/streptomycin solution (Diagnovum, D910). Both cell lines were cultured at 37 °C in a humidified incubator with 5% CO₂, and were confirmed to be mycoplasma-free before use.

**Fluorescence Studies**

Binding selectivity of synthesized compounds toward G-quadruplex-forming RNA (RNA TERRA, DNA TERRA, and MT3) over non-G4 controls (anti-TERRA and TERRA-mutant) was evaluated by monitoring changes in fluorescence intensity. Briefly, increasing concentrations of RNA or DNA sequences were titrated into a fixed concentration of compounds in 1× folding buffer (10 mM Tris-HCl, pH 7.6, and 100 mM KCl). Prior to titration, nucleic acids were folded by heating to 95 °C for 5 minutes and gradually cooled to room temperature. Fluorescence measurements were performed using the Synergy H1 Hybrid Multi-Mode Microplate Reader (BioTek, Agilent), and changes in fluorescence were analyzed to assess the relative binding preferences of each compound.

Half-maximal effective concentration (EC₅₀) measurements for the interaction between synthesized compounds and 5'–FAM–labeled TERRA RNA were performed by monitoring changes in fluorescence intensity as a function of compound concentration. Briefly, the RNA was folded in 1× folding buffer (10 mM Tris-HCl, pH 7.6, and 100 mM KCl) by heating at 95 °C for 5 minutes, followed by slow cooling to room temperature. Compound solutions were prepared in the same buffer. Compounds were titrated into a fixed concentration of RNA (1 µM), starting from 20 µM and serially diluted (1:2) down to 7.32 nM, yielding a total of 24 concentrations. EC₅₀ values were determined from plots of compound concentration versus fluorescence change, measured using the Synergy H1 Hybrid Multi-Mode Microplate Reader (BioTek, Agilent).

For the nuclease sensitivity assay, cells were seeded in 96-well plates and, at ~90% confluency, fixed with 4% (w/v) paraformaldehyde in PBS at room temperature, washed ×3 with cold PBS, and permeabilized with 0.2% Triton X-100 for 30 min at 37 °C. After washing, cells were incubated for 3 h with DNase I or RNase A (200 U mL⁻¹ in PBS) or left untreated, followed by addition of RIBO-ISCH-1 (20 µM). Kinetic fluorescence readouts were collected on a Synergy H1 Hybrid Multi-Mode Microplate Reader (BioTek, Agilent) for 2.5 h using the appropriate excitation/emission settings for the fluorophore, and the fluorescence change (ΔF relative to the pre-addition baseline) was calculated.

For determining binding affinity (Kd), RNA TERRA, DNA TERRA, RNA MT3, TERRA-mutant, or anti-TERRA were folded in 1× folding buffer (10 mM Tris-HCl, pH 7.6, 100 mM KCl) by heating at 95 °C for 5 min followed by slow cooling to room temperature. Increasing concentrations of the folded nucleic acid were titrated into a fixed concentration of RIBO-ISCH-1 prepared in the same buffer. Titrations comprised 24 twofold serial dilutions from a top strand concentration selected to reach signal saturation. Fluorescence was recorded on a Synergy H1 Hybrid Multi-Mode Microplate Reader (BioTek, Agilent).

**Fluorescence-Based *In vitro* RNA Cleavage Assay**

RNA degradation was assessed by monitoring the decrease in fluorescence upon addition of RNase L to pre-folded RNA-compound mixtures. Briefly, RNA was folded in 1× folding buffer (10 mM Tris-HCl, pH 7.6, and 100 mM KCl) by heating at 95 °C for 5 minutes, followed by slow cooling to room temperature. Folded RNA was then mixed at a fixed concentration with synthesized compounds at a fixed concentration. RNase L was prepared in its activation buffer (7 mM β-mercaptoethanol, 50 µM ATP, and 1 mM MgCl₂), heated at 95 °C for 5 minutes, and rapidly cooled on ice for 5 minutes. It was then added to one set of triplicate wells. A second triplicate containing RNA, compound, and activation buffer (without RNase L) served as a negative control. Changes in fluorescence were measured using the Synergy H1 Hybrid Multi-Mode Microplate Reader (BioTek, Agilent).

**General protocol for mRNA RT-qPCR**

HeLa or U2OS cells were seeded in 6-well plates at a density of approximately 250,000 cells per well (60–70% confluency) and incubated for 12 hours. Cells were then treated with the indicated concentrations of synthesized compounds for the specified durations. Following treatment, total RNA was extracted using the Quick-RNA Miniprep Kit (Zymo Research) according to the manufacturer’s protocol, including on-column DNase I treatment. Reverse transcription was performed on 1 µg of total RNA using the LunaScript® RT SuperMix Kit (New England BioLabs, cat #M3010), following the manufacturer's instructions. Approximately 30 ng of cDNA was used for each qPCR reaction, which was carried out using the Luna® Universal qPCR Master Mix (New England BioLabs, cat. #E3010G) on a CFX Opus 384 Real-Time PCR System (Bio-Rad). Relative expression levels of *NRAS*, *KRAS*, *FGF2*, *BCL2*, *ADAM10*, *MT3*, *VEGFA*, *APC*, *ACVR1C*, *CCND3*, *CTNNB1*, *CTSB*, *GRIA1*, *HIRA*, *IGF2*, *THRA, RNase L, TERRA7p, TERRA13q, TERRA15q,* and *TERA20q* were quantified by normalizing to *GAPDH* expression using the ΔΔCt method.

**Competition treatment**

HeLa cells were seeded in 6-well plates at a density of approximately 250,000 cells per well (60–70% confluency) and incubated for 12 hours. Cells were first treated with increasing concentrations of ISCH for 5 hours, followed by treatment with a fixed concentration of RIBO-ISCH-1 for an additional 48 hours.

**SiRNase L / TERRA ASO treatment**

For experiments involving siRNA or ASO treatments, INTERFERin® transfection reagent (Polyplus, cat. #101000028) was used. For qPCR analysis, cells were transfected with siRNase L (10 nM) 6 hours prior to treatment with either ISCH or RIBO-ISCH-1. ASO or scramble control treatments (50 nM) were applied for 12 hours in qPCR, FACS, and Western blot experiments.

**General protocol for western blotting**

U2OS cells were grown in 6-well plates at about 60% confluency in complete growth medium, and the cells were treated with different synthesized compounds and controls at the indicated concentration or vehicle for 48 hr. Total protein was extracted using RIPA cell lysis buffer (BioPrep) containing protease inhibitor, and protein concentration was measured using BCA protein assay (Sigma-Aldrich) according to the manufacturer's protocol. Approximately 50 μg of total protein was resolved on a 10% SDS-acrylamide gel and then transferred to a PVDF membrane (Immobilon FL transfer membrane- Merck). The membrane was washed with 1X Tris-buffered saline (TBS) containing 0.1% (v/v) Tween-20 (TBST; Tris-base, pH 7.6, NaCl and Tween-20), and then blocked in 1x TBST containing 5% (w/v) BSA for 2 hr at room temperature. The membrane was then incubated with 1:1000 dilution of rabbit anti-FANCD2 (Abcam: ab108928), 1:1000 dilution of mouse anti-RAD51 (Abcam: ab88572), 1:1000 dilution of rabbit anti-cleaved caspase-3 (Cell Signaling Technology: #9661), 1:1000 dilution of rabbit anti-RNase L (Cell Signaling Technology: #27281), 1:1000 dilution of rabbit anti-GAPDH (Cell Signaling Technology:14C10) or 1:1000 dilution of rabbit anti-Vinculin (Cell Signaling Technology: CS13901) in 1x TBST containing 5% (w/v) BSA overnight at 4 °C. The membrane was then washed with 1x TBST and incubated with 1:10,000 anti-rabbit IgG horseradish-peroxidase secondary antibody conjugate (Cell Signaling: CS7074) or anti-mouse IgG horseradish-peroxidase secondary antibody conjugate (Cell Signaling: CS7076) at room temperature for 2 hr. After washing three times with 1× TBST (10 min per wash), the target protein was detected by using SuperSignal West Pico PLUS Chemiluminescent Substrate (Thermo Scientific) on Azure C300 imaging System. The fold change of the target protein expression was calculated by normalizing the band intensity to housekeeping protein Vinculin or GAPDH band intensity using ImageJ.

**Cell Counting- Proliferation assay**

U2OS cells were seeded in 96-well plates to 60% confluency and treated with synthesized compounds or controls at four different concentrations (0.01 µM –10 µM) for 48 hours. Cell proliferation was assessed using the Resazurin Assay Kit (Abcam, ab228554), where each sample was incubated with 1X WST-8/CCK8 solution for 1 hour. After incubation, fluorescence was measured by absorbance at 460 nm using the Synergy H1 Hybrid Multi-Mode Microplate Reader (Biotek, Agilent). Cell proliferation was calculated as the percentage decrease in fluorescence of treated cells compared to untreated controls. All experiments were performed in triplicate.

**Flow Cytometry**

U2OS cells were seeded onto 6 well p[lates and treated with ISCH, RIBO-ISCH-1, ASO, or Scramble for 48 hours then was collected into 2 mL microcentrifuge tubes and washed with 1 mL of FACS buffer (0.1% BSA in PBS), followed by centrifugation at 400 × g for 5 minutes. The cell pellets were resuspended in 200 µL of 2% paraformaldehyde (PFA) in PBS and incubated at room temperature for 10 minutes in the dark. After fixation, cells were washed twice with FACS buffer and resuspended in 200 µL of 0.2% Triton X-100 in PBS for permeabilization. Samples were incubated for 10 minutes at room temperature, followed by a PBS wash. Cells were then incubated with anti-γH2AX primary antibody (Cell Signaling, cat. CST- 9718, 1:200 dilution in FACS buffer (500 µL per sample)) for 1 hour on ice. Following primary antibody staining, cells were washed 2–3 times with FACS buffer and incubated with Alexa Fluor–conjugated secondary antibody (Abcam, Cat. AB-ab150077, 1:2000 dilution in FACS buffer) for 45 minutes on ice, protected from light. After staining, cells were washed 2–3 times with FACS buffer and resuspended in 1 mL PBS. Prior to acquisition, samples were passed through a 70 µm cell strainer into FACS tubes and kept on ice. Data were acquired using CytoFLEX cytometer and analysis was performed using FlowJo software (FlowJo_v10.10). cytometry software.

**Fluorescence Microscopy**

HeLa or U2OS cells were seeded onto glass coverslips in a 12-well plate. Upon reaching 80 % confluency, the cells were treated with either ISCH (0.1 µM) or RICO-ISCH-1 (0.1 µM) for 48 hours. Following treatment, the cells were washed three times with PBS and fixed with 4 % PFA (500 μL) in PBS for 10 minutes at room temperature, followed by three washes with ice-cold PBS. The cells were then permeabilized with 0.2 % Triton X-100 in PBS for 10 minutes at room temperature, followed by three washes with PBS. Blocking was performed using 500 μL of blocking buffer composed of 1 % BSA and 22.52 mg/mL glycine in PBST (PBS + 0.1 % Tween 20) for 1 hour at room temperature. The cells were then incubated with Rabbit Anti-PML Monoclonal primary antibody (Cell Signaling, Cat. CST-69789, 1:500 dilution in 1 % BSA in PBST) for 1 hour at room temperature, followed by an overnight incubation at 4 °C. The next day, the cells were washed three times with PBS for 5 minutes each and incubated with the secondary antibody Goat Anti-Rabbit IgG H&L Alexa Fluor® 488 (Abcam, Cat. AB-ab150077, 1:1000 dilution in 1 % BSA in PBS) for 1 hour at room temperature. The cells were then washed three times with PBS for 5 minutes each, followed by mounting with Prolong Gold Antifade Reagent with DAPI (Cell Signaling, Cat. 8961S) and sealing the coverslips with nail polish. The slides were imaged using a NIKON New AX-R Confocal microscope at 60x magnification. Images were analyzed and processed using NIS-Elements AR 5.2.

**Colony Formation Assay**

U2OS cells were seeded in 6-well plates at a density of 1,000 cells per well and incubated for 12 hours. Cells were then treated with either ISCH (1 µM) or RIBO-ISCH-1 (1 µM) and cultured for 21 days, with treatment refreshed every 3 days. At the end of the incubation period, cells were fixed and stained with 1% crystal violet solution. Plates were imaged to visualize positively stained colonies in each well.

**Quantification and statistical analysis**

Images were processed and analyzed using ImageJ software. Statistical analyses were conducted using GraphPad Prism 10.5.0. All results were presented as mean ± SEM. All experiments were done in triplicate unless otherwise specified. Statistical comparisons between two groups were performed using an unpaired two-tailed Student's *t*-test. For multiple comparisons, data were analyzed using one-way ANOVA. Significance levels were denoted as follows: * *P* < 0.05, ** *P* < 0.01, *** *P* < 0.001, **** *P* < 0.0001; ns indicates no significant difference.

**5. Figures and Tables**

**Table S1.** Sequences for RNA and DNA oligonucleotides used in this study.

| **Structure** | **Name** | **Sequence 5’ to 3’** | **Supplier** |
| --- | --- | --- | --- |
| DNA | TERRA | TTAGGGTTAGGGTTAGGGTTAGGG | IDT |
|  | Anti-TERRA | CCCTAACCCTAACCCTAACCCTAA | IDT |
| RNA | TERRA | UUAGGGUUAGGGUUAGGGUUAGGG | IDT |
|  | TERRA-mutant | UUACCGUUACCGUUACCGUUACCG | IDT |
|  | MT3 | GGGAGGGAGGGAGAGGGA | IDT |
|  | 5’FAM-TERRA | FAM-UUAGGGUUAGGGUUAGGGUUAGGG | IDT |
| ASO | ASO-TERRA | TAACCCTAACCCTAAC | IDT |
|  | Scramble-ASO | CACGTCTATACACCAC | IDT |
| SiRNA | SiRNase-L  hs.Ri.RNASEL.13.1 | GAGAACAGUCACUUGGUGACAUUCT | IDT |
|  | Scramble-SiRNA | CAACCUACUUUCGAGAGGAAGAAGA | IDT |

**Table S2.** Primers used for RT-qPCR.

| **Name** | **Sequence (5’ to 3’)** | **Supplier** |
| --- | --- | --- |
| ADAM10 F primer | AGCAACATCTGGGGACAAAC | IDT |
| ADAM10 R primer | CCCAGGTTTCAGTTTGCATT | IDT |
| Bcl2 F primer | ACTGGCTCTGTCTGAGTAAG | IDT |
| Bcl2 R primer | CCTGATGCTCTGGGTAAC | IDT |
| FGF2 F primer | CTGGCTATGAAGGAAGATGGA | IDT |
| FGF2 R primer | TGCCCAGTTCGTTTCAGTG | IDT |
| GAPDH F primer | AGCCACATCGCTCAGACAC | IDT |
| GAPDH R primer | GCCCAATACGACCAAATCC | IDT |
| KRAS F primer | GCCTGCTGAAAATGACTG | IDT |
| KRAS R primer | TCCTGTAGGAATCCTCTATTG | IDT |
| MT3 F primer | CAAGTGGCAGGGATGCAAATGC | IDT |
| MT3 R primer | GCACTTCTCTGCTTCTGCCTCA | IDT |
| NRAS F primer | CAGAGGCAGTGGAGCTTGA | IDT |
| NRAS R primer | GCTTTTCCCAACACCACCT | IDT |
| RNase L F primer | AAGGCTGTTCAAGAACTACACTTG | IDT |
| RNase L R primer | TGGATCTCCAGCCCACTTGATG | IDT |
| HIRA F primer | ТCTATGGACGGCTCTGTGGCAT | IDT |
| HIRA R primer | TAGGCTCTTGCCATAGGTGGAC | IDT |
| VEGF F primer | TTGCCTTGCTGCTCTACCTCCA | IDT |
| VEGF R primer | GATGGCAGTAGCTGCGCTGATA | IDT |
| APC F primer | AGGCTGCATGAGAGCACTTGTG | IDT |
| APC R primer | CACACTTCCAACTTCTCGCAACG | IDT |
| ACVR1C F primer | TGCTAGTGGTCTGGCACACCTT | IDT |
| ACVR1C R primer | CTTCACAGCCAACCCTAAGTCC | IDT |
| CCND3 F primer | ACGCAAGACAGGTAGCGATCCA | IDT |
| CCND3 R primer | ACGCAAGACAGGTAGCGATCCA | IDT |
| CTSB F primer | GCTTCGATGCACGGGAACAATG | IDT |
| CTSB R primer | CATTGGTGTGGATGCAGATCCG | IDT |
| CTNNB1 F primer | CACAAGCAGAGTGCTGAAGGTG | IDT |
| CTNNB1 R primer | GATTCCTGAGAGTCCAAAGACAG | IDT |
| GRIA1 F primer | GGATGCTCTTTCAGGACCTGGA | IDT |
| GRIA1 R primer | GTAGTGGTAGCCGATGCCATTC | IDT |
| IGF2 F primer | TGGCATCGTTGAGGAGTGCTGT | IDT |
| IGF2 R primer | ACGGGGTATCTGGGGAAGTTGT | IDT |
| THRA F primer | TGGATGACACGGAAGTGGCTCT | IDT |
| THRA R primer | TACGCCTCCTGACTCTTCTCGA | IDT |
| 7p sub-telomeric F primer | GGAGGCTGAGGCAGGAGAA | IDT |
| 7p sub-telomeric R primer | CAATCTCGGCTCACCAATC | IDT |
| 13q sub-telomeric F primer | CTGCCTGCCTTTGGGATAA | IDT |
| 13q sub-telomeric R primer | AAACCGTTCTAACTGGTCTCTG | IDT |
| 15q sub-telomeric F primer | GCTGCATTAAAGGGTCCAGT | IDT |
| 15q sub-telomeric R primer | AACCCTAACCACATGAGCAACG | IDT |
| 20q sub-telomeric F primer | GAAGTTGCTGGGTTCTATGG | IDT |
| 20q sub-telomeric R primer | ATGGTGCAGACACTGTGG | IDT |

**Figure S1.** Fluorescence titration of 5 µM **ISCH** with stepwise addition of different sequences in 10 mM Tris-HCl buffer, 100 mM KCl, pH 7.4, λex = 550 nm. (A) Describes the changes in emission intensity upon adding RNA TERRA. (B) Describes the change in emission intensity upon adding TERRAmut, antiTERRA, DNA TERRA, and MT3.


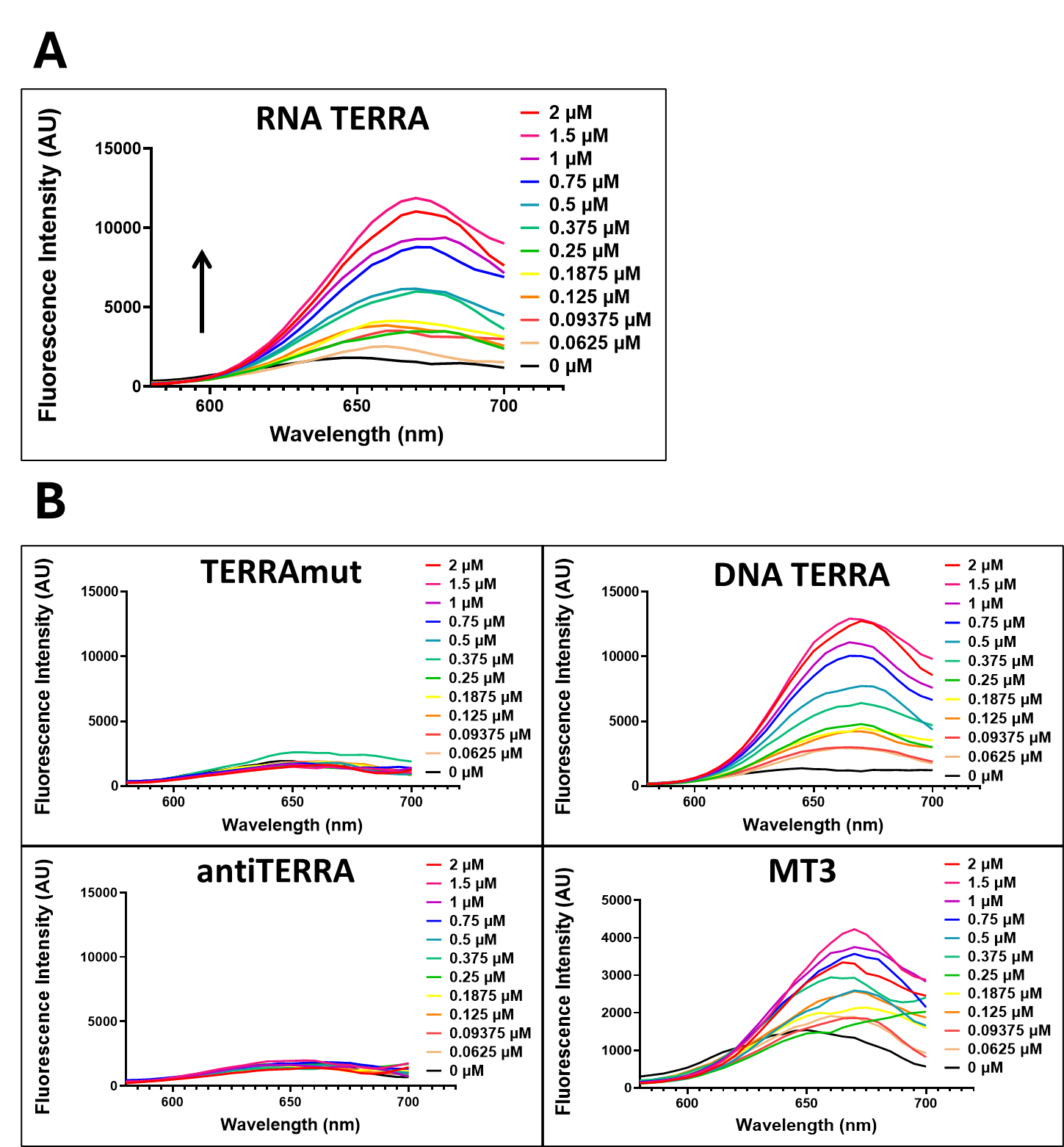


**Figure S2.** Fluorescence titration of 40 µM **RIBO-ISCH-1** with stepwise addition of different sequences in 10 mM Tris-HCl buffer, 100 mM KCl, pH 7.4, λex = 550 nm.

**
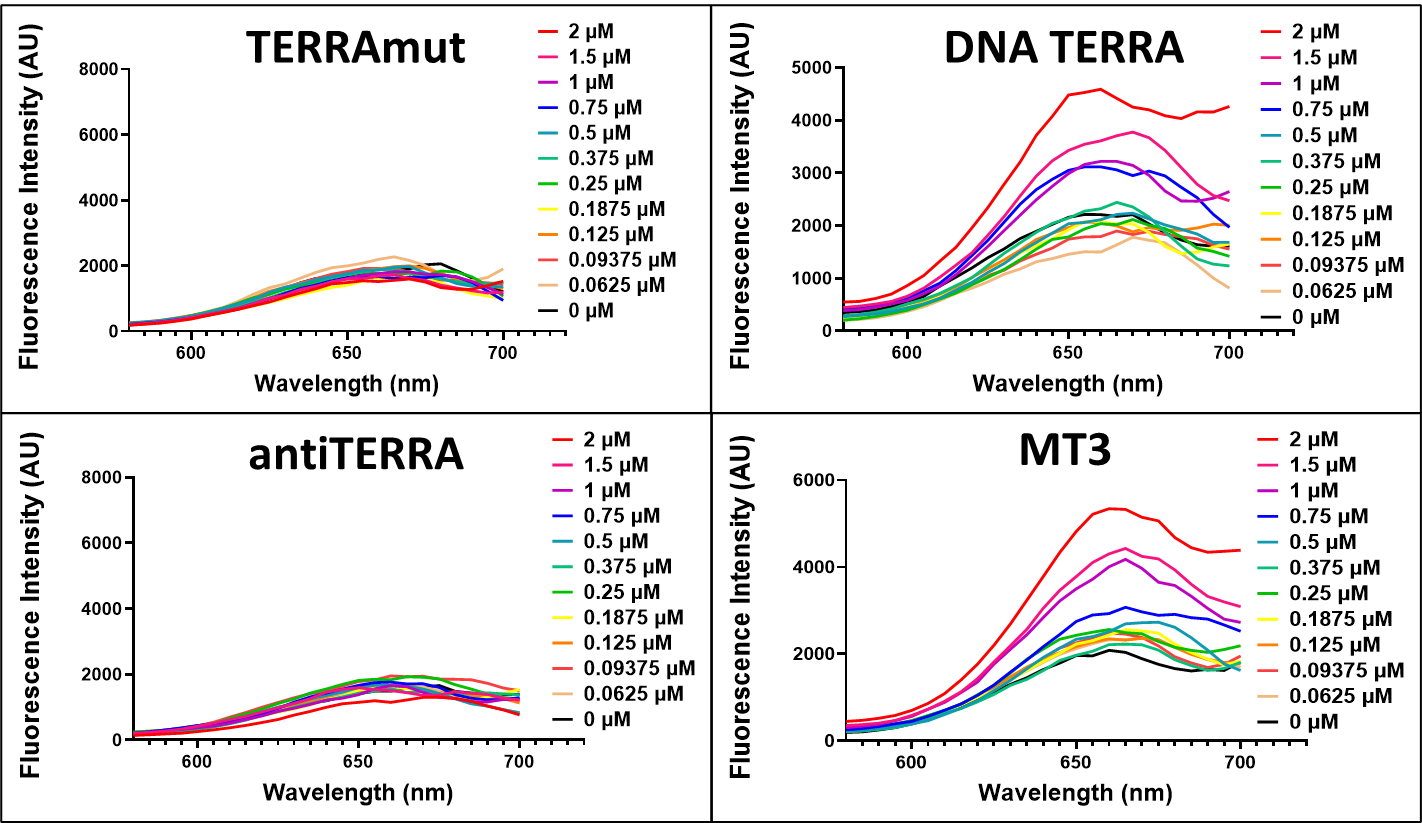
**

**
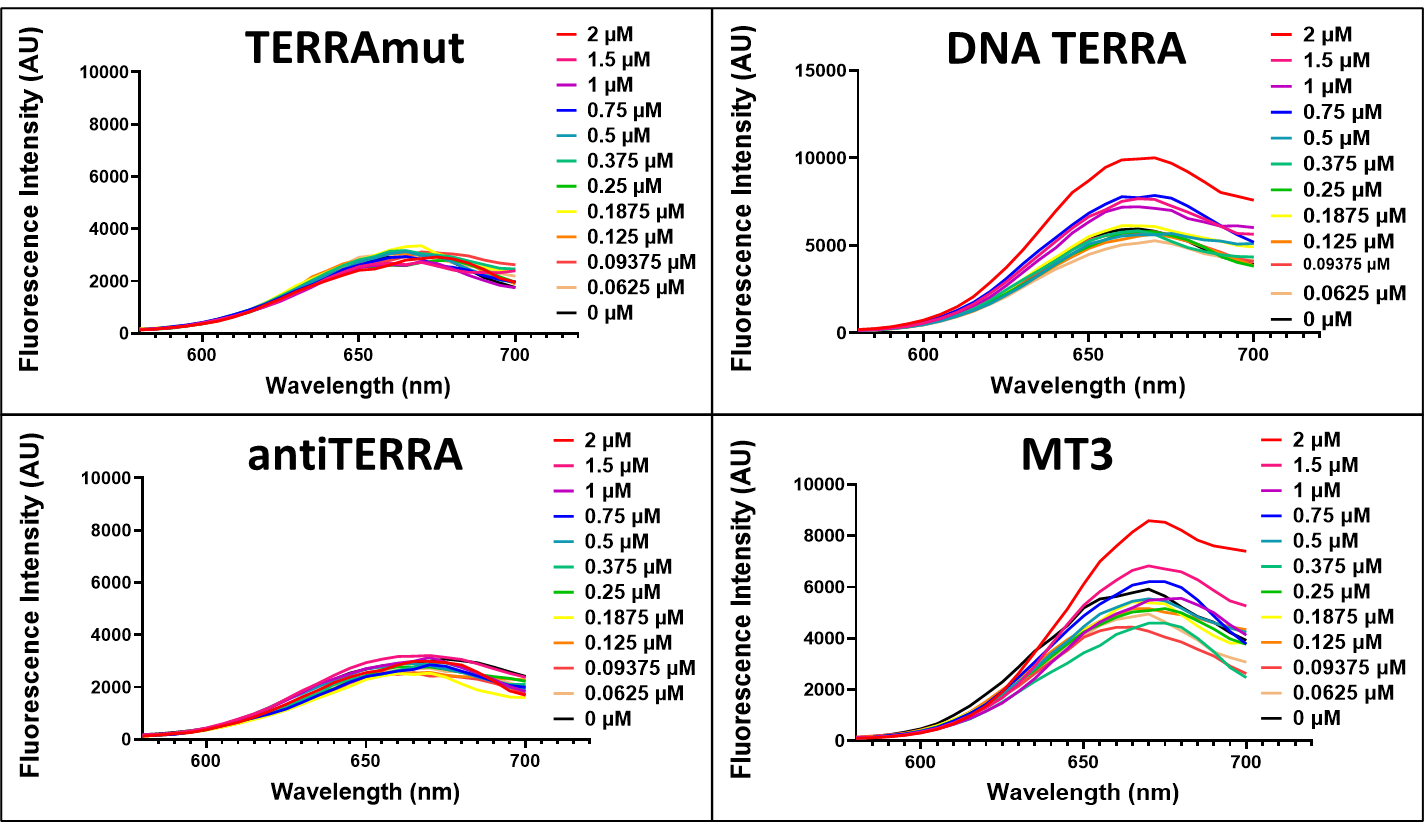
Figure S3.** Fluorescence titration of 40 µM **RIBO-ISCH-2** with stepwise addition of different sequences in 10 mM Tris-HCl buffer, 100 mM KCl, pH 7.4, λex = 550 nm.

**Figure S4.** Fluorescence titration of 40 µM **RIBO-ISCH-3** with stepwise addition of different sequences in 10 mM Tris-HCl buffer, 100 mM KCl, pH 7.4, λex = 550 nm.

**
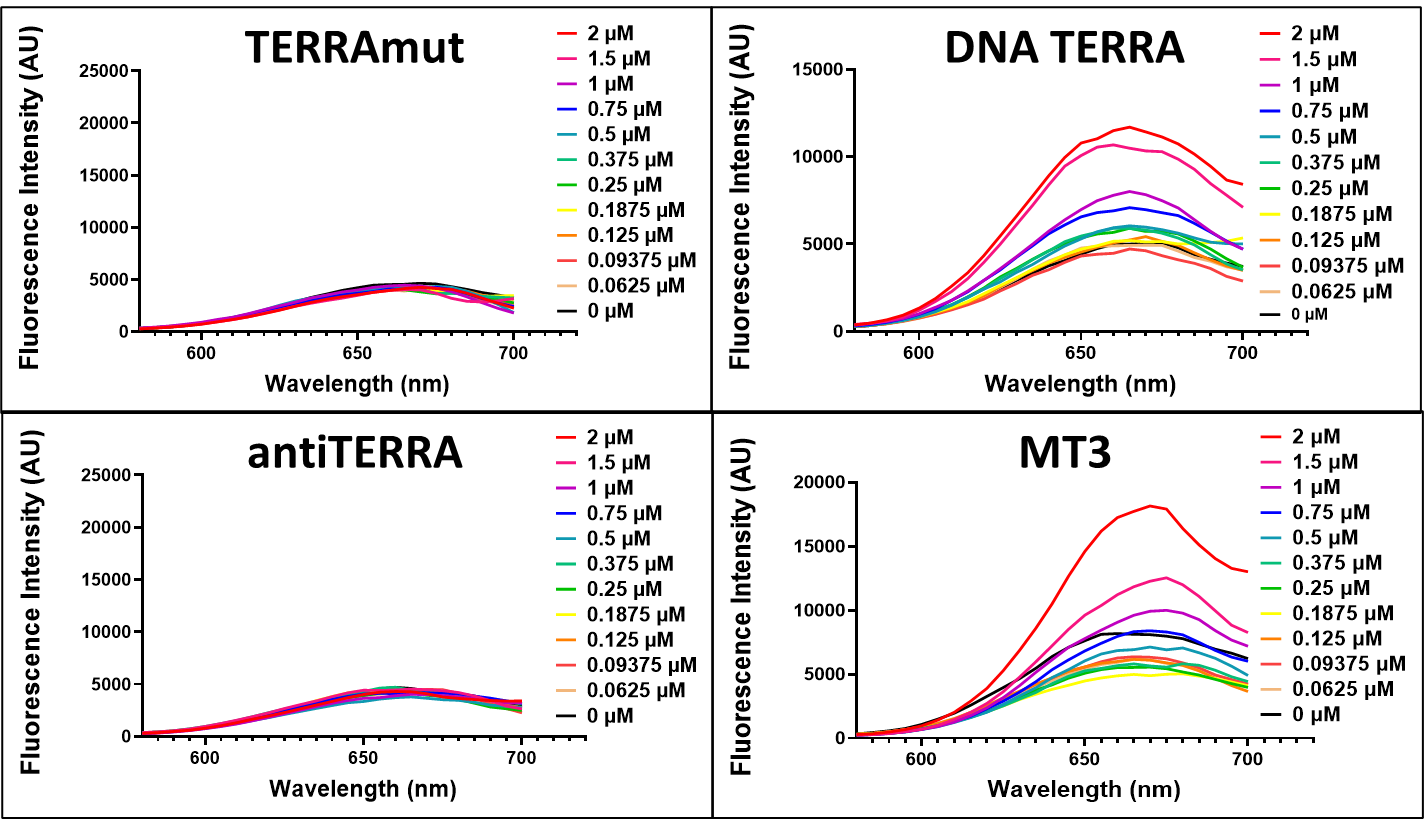
**

**
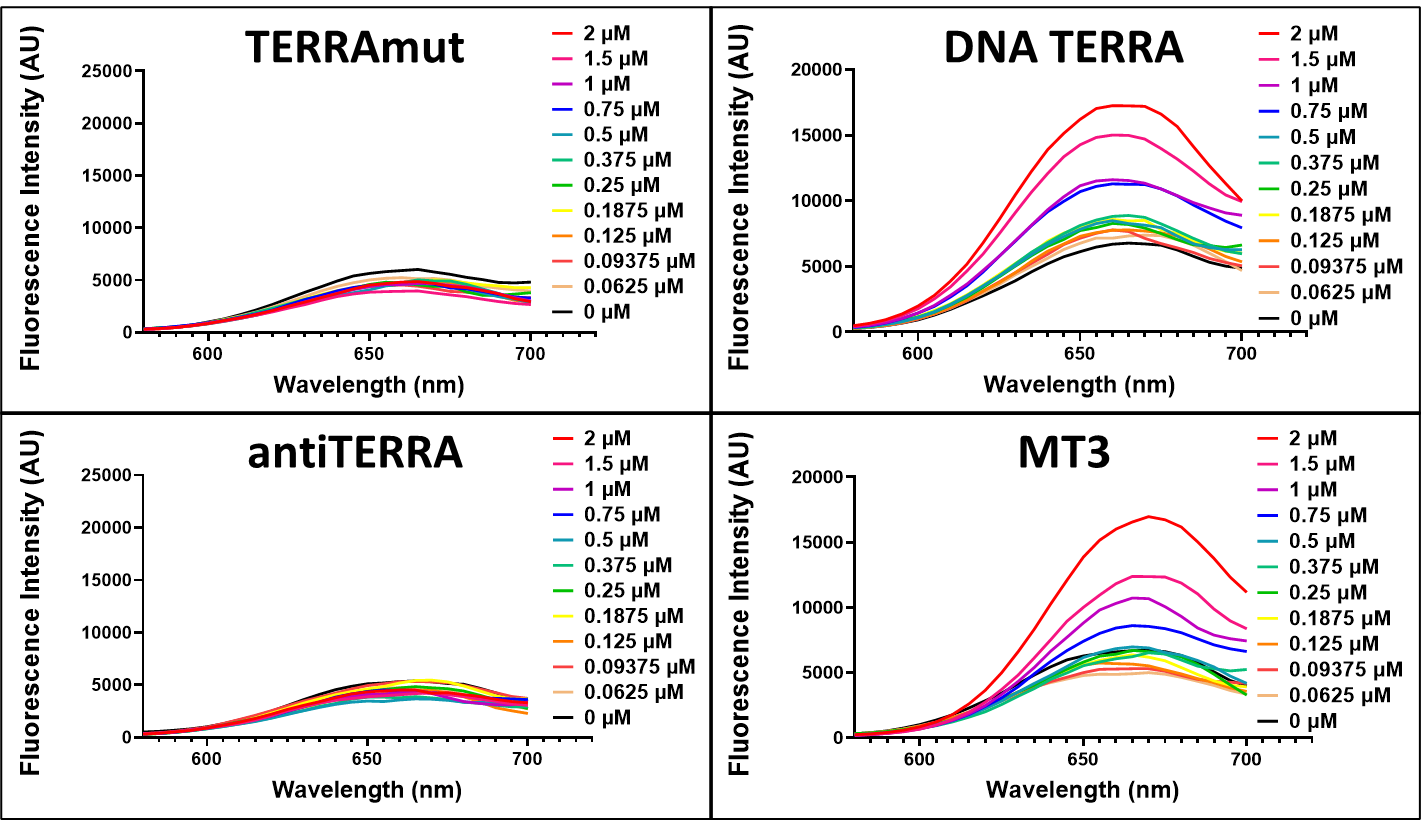
Figure S5.** Fluorescence titration of 40 µM **RIBO-ISCH-4** with stepwise addition of different sequences in 10 mM Tris-HCl buffer, 100 mM KCl, pH 7.4, λex = 550 nm.

**Figure S6.** Fluorescence-based binding assay of RIBO-ISCH-1 with various nucleic acid structures. Binding was assessed by titrating increasing concentrations of each sequence into a fixed concentration of 40 µM RIBO-ISCH-1 in 10 mM Tris-HCl buffer, 100 mM KCl, pH 7.4. Sequences tested included RNA TERRA, MT3, DNA TERRA, TERRA-mut, and anti-TERRA. (λex = 550 nm, λem = 660 nm)

**
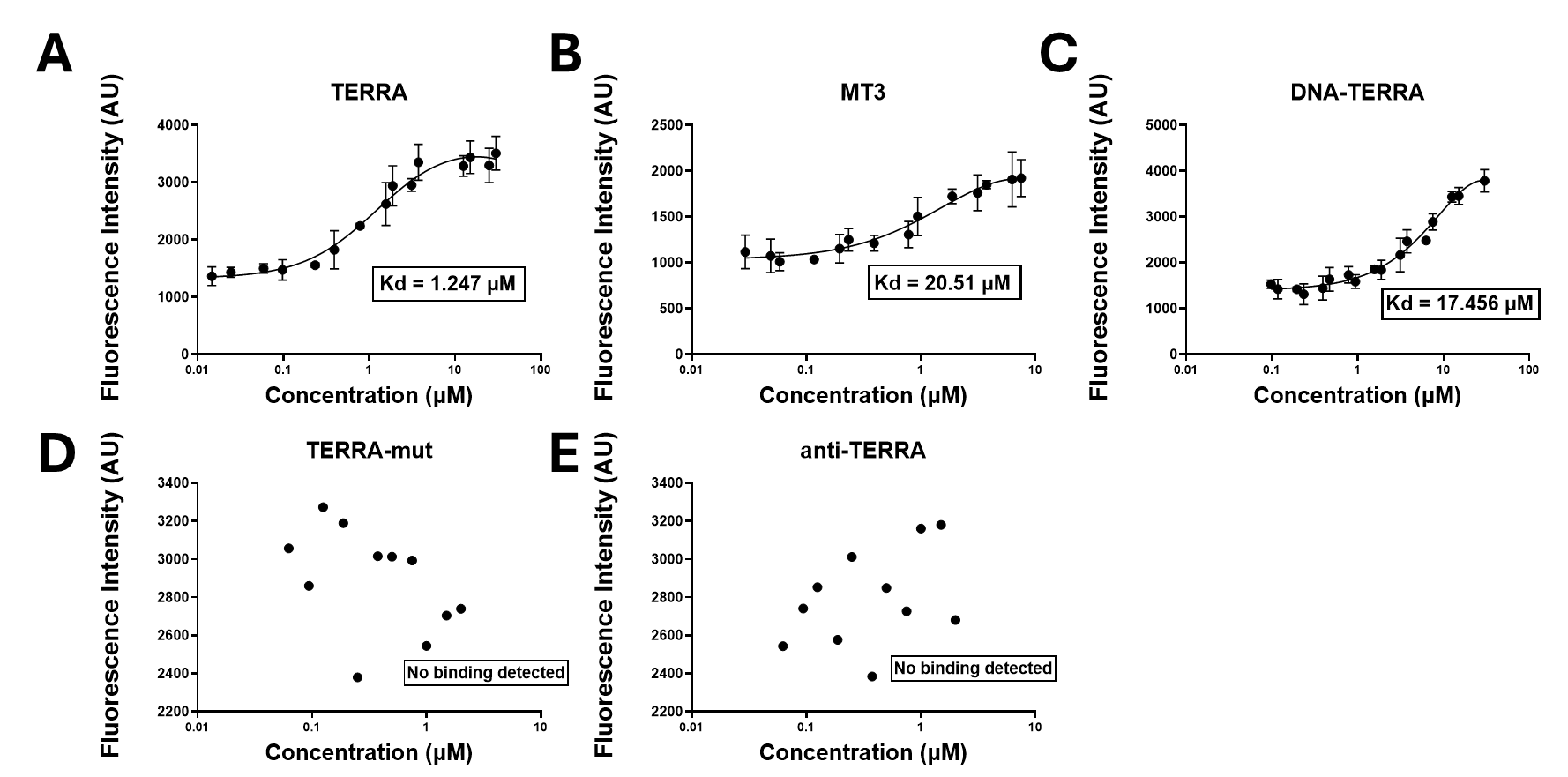
**

**Figure S7.** Fluorescence intensities at 660 nm of **ISCH** (5 µM) or RIBOTACs (40 µM) incubated with TERRA, TERRAmut, or antiTERRA (2 µM) in KCl-based G-quadruplex folding buffer, or with TERRA in CuSO₄-based unfolding buffer. Statistical comparison was performed by unpaired *t*-test (*****p* < 0.0001).

**
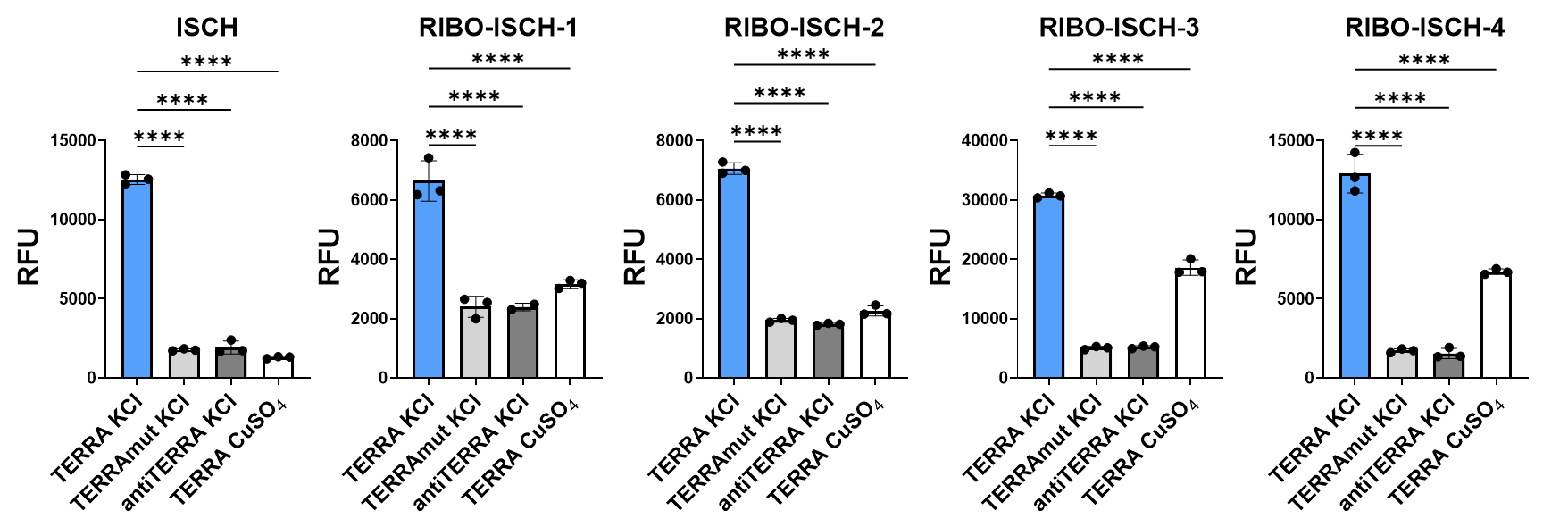
**

**Figure S8.** Binder-only control for RNase L background activity. Normalized fluorescence intensity of pre-folded TERRA (2 µM) complexed with ISCH (5 µM) following RNase L addition, measured at t_0_, 1 h, 2 h, and 2.5 h (150 min). Bars represent mean ± SEM.

**Figure S9.** Validation of RT-qPCR primers used to measure levels of *TERRA-7p*, *TERRA-13q*, *TERRA-15q*, and *TERRA-20q*, *RNase L*, and *GAPDH* genes by RT-qPCR. (A) CT values across serial cDNA dilutions from reverse transcription. (B) Melting curves confirm specific amplification of a single product per gene. (C) No-template controls show no amplification (Ct > 32).


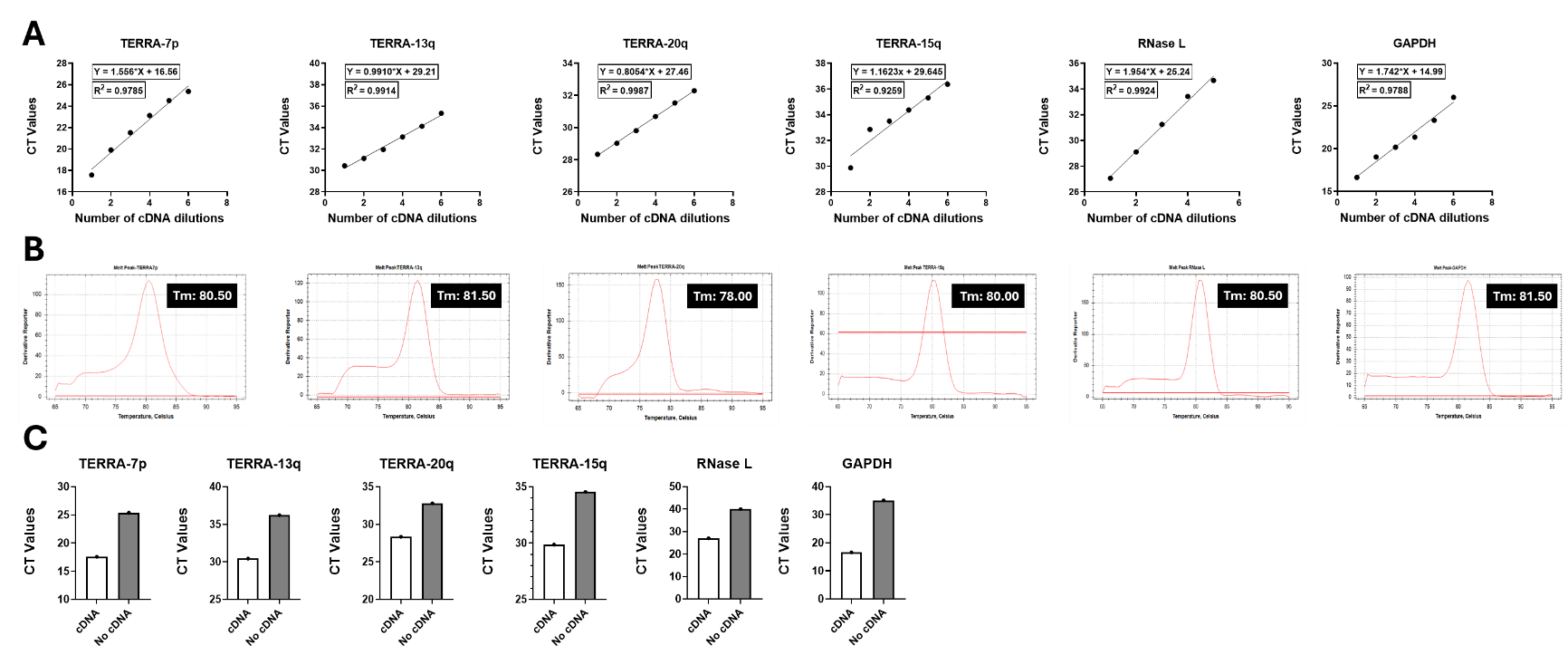


**Figure S10.** Validation of RT-qPCR primers used to measure levels of *NRAS*, *KRAS*, *FGF2*, *BCL2*, *ADAM10*, and *MT3* genes by RT-qPCR. (A) CT values across serial cDNA dilutions from reverse transcription. (B) Melting curves confirm specific amplification of a single product per gene. (C) No-template controls show no amplification (Ct > 32).


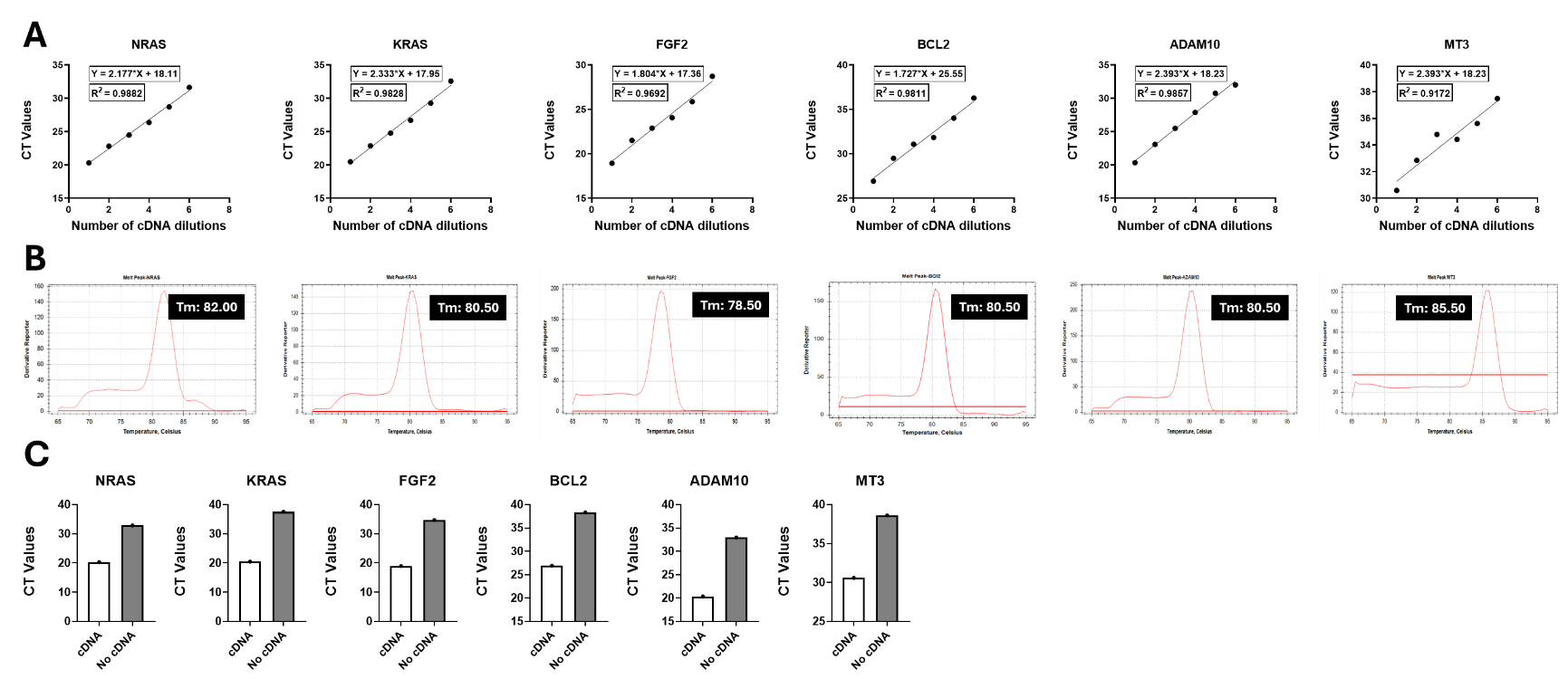


**Figure** **S11.** Time-dependent evaluation in HeLa cells treated with 0.1 µM **RIBO-ISCH-1**. (A) Relative abundance of *TERRA-7p,* as determined by RT-qPCR, (B) Relative abundance of *TERRA-20q*, as determined by RT-qPCR. Data are normalized by the mean expression level in untreated control cells. Statistical comparison was performed by one-way ANOVA (*****p* < 0.0001; ***p* < 0.01; **p* < 0.05).

**
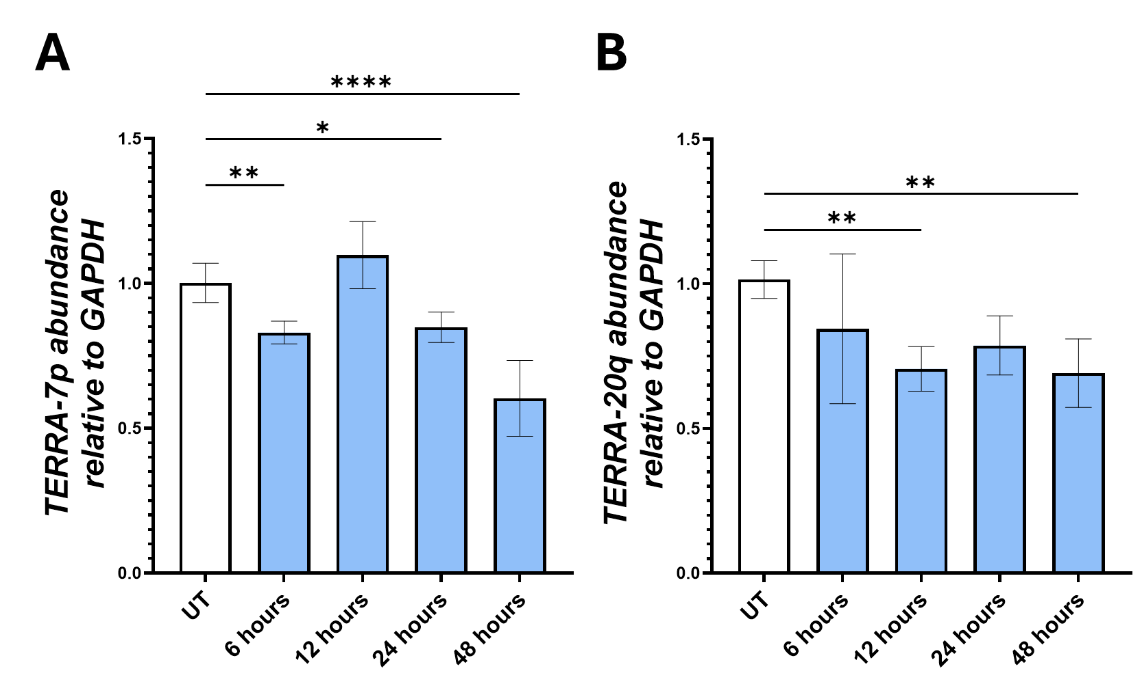
**

**Figure S12.** (A) Relative abundance of *TERRA-7p* in HeLa cells treated with Mock, scramble control, or ASO-TERRA, as determined by RT-qPCR. (B) Relative abundance of *TERRA-13q* in HeLa cells treated with Mock, scramble control, or ASO-TERRA, as determined by RT-qPCR. (C) Relative abundance of *TERRA-20q* in HeLa cells treated with Mock, scramble control, or ASO-TERRA, as determined by RT-qPCR. Data are normalized by the mean expression level in untreated control cells. Statistical comparisons were performed using unpaired t-test (****p* < 0.001; ***p* < 0.01; **p* < 0.05).

**
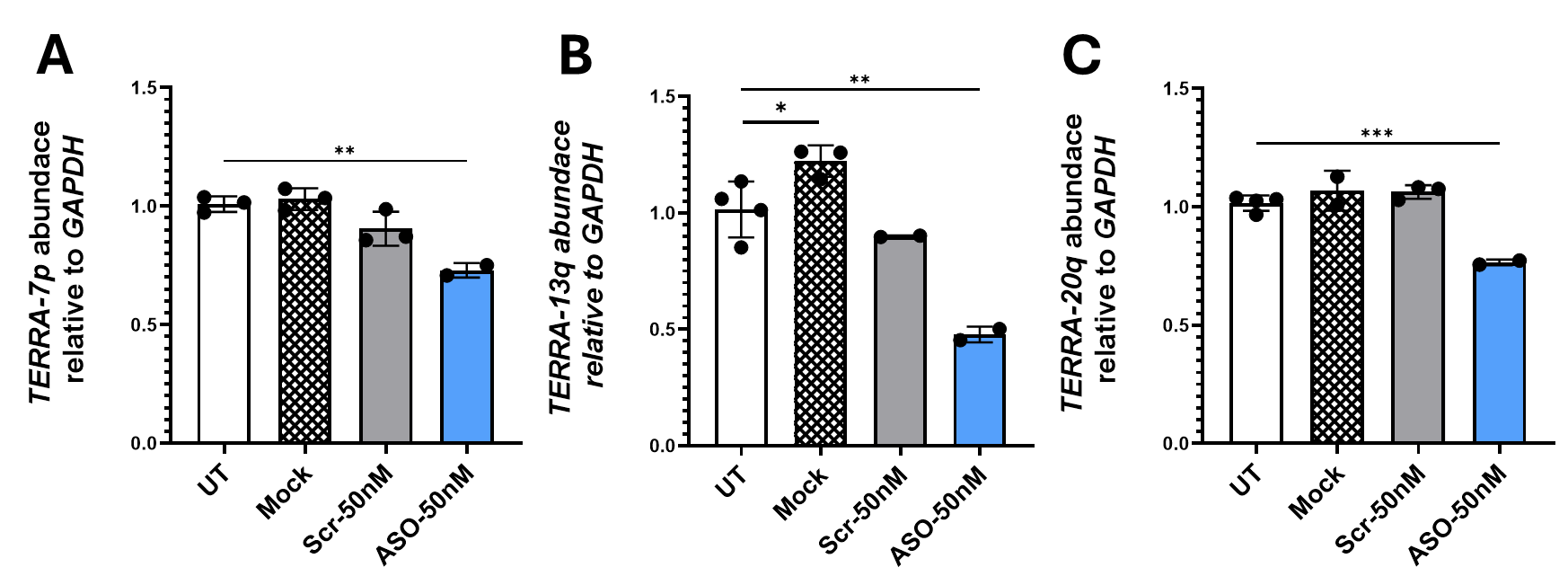
**

**Figure S13.**  Relative abundance of *TERRA-15q* in U2OS cells following dose-dependent treatment with **RIBO-ISCH-1** and a fixed 1 µM dose of ISCH for 48 hours, as determined by RT-qPCR. Data are normalized by the mean expression level in untreated control cells. Statistical comparison was performed by one-way ANOVA (****p* < 0.001; **p* < 0.05).

**
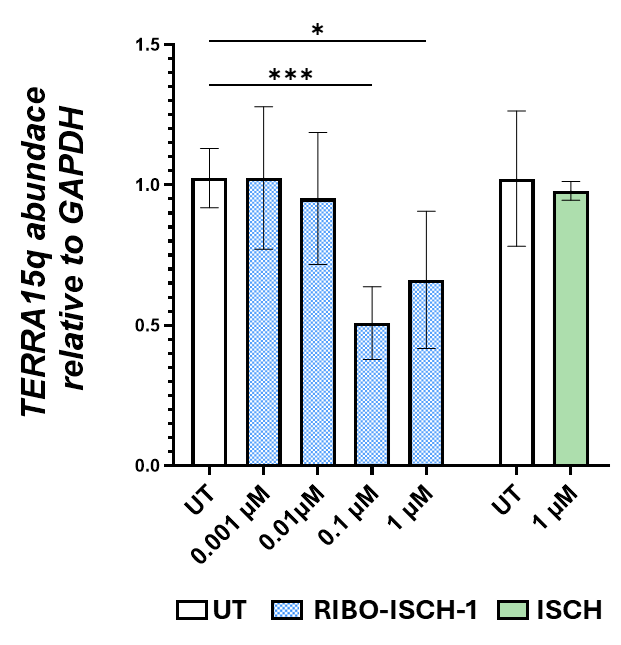
**

**Figure** **S14.** Bioinformatic prioritization of G-quadruplex candidates.
(A) Candidate genes ranked by G% (x-axis). Bars are colored by the Scott Lab bioinformatics classification: blue = True, green = False. Asterisks denote genes selected for follow-up experiments. (B) QGRS-mapper scores for genes taken forward to RT-qPCR selectivity analyses. Score interpretation: >30 = strong, 40–60 = very strong, >60 = exceptional predicted G4-forming potential.

Database URL: http://scottgroup.med.usherbrooke.ca/G4RNA/


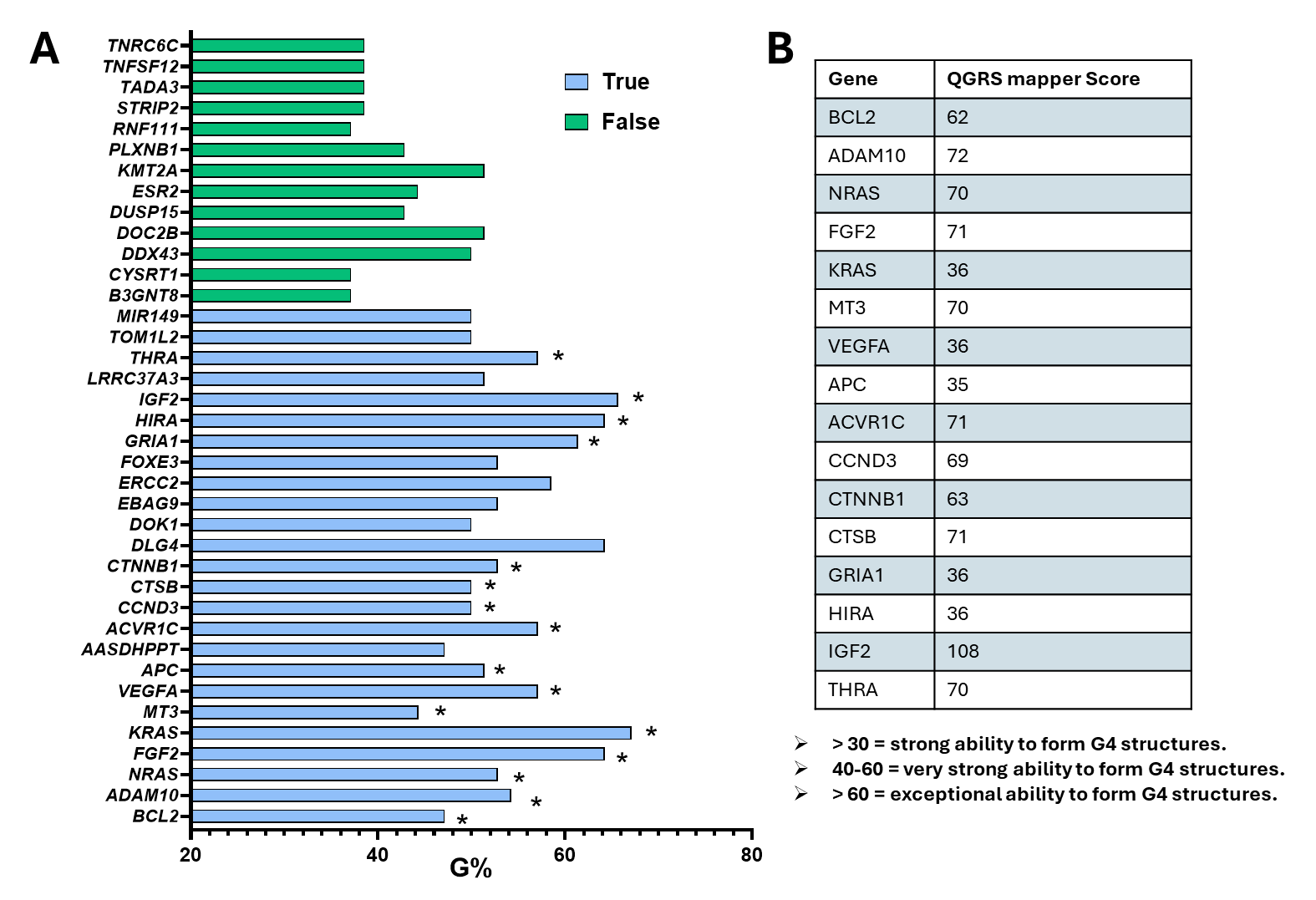


**Figure** **S15.** Relative abundance of *TERRA* and other G4-containing transcripts (*NRAS*, *KRAS*, *FGF2*, *ADAM10*, *BCL2*, and *MT3*) following treatment with RIBO-ISCH-1 (100 nM) for 48 hours in HeLa cells, as determined by RT-qPCR. Data are normalized by the mean expression level in untreated control cells. Statistical comparisons were performed using unpaired *t-*test (**p* < 0.05; ****p* < 0.001).

**Figure** **S16.** (A) Relative abundance of G4 RNAs in HeLa cells treated with 0.1 µM **RIBO-ISCH-2** for 48 hours, as determined by RT-qPCR. Data are normalized by the mean expression level in untreated control cells. Statistical comparisons were performed using unpaired t-test (**p* < 0.05). (B) Relative abundance of G4 RNAs in HeLa cells treated with 0.1 µM **ISCH** for 48 hours, as determined by RT-qPCR. (C) Relative abundance of G4 RNAs in U2OS cells treated with 0.1 µM **ISCH** for 48 hours, as determined by RT-qPCR.

**
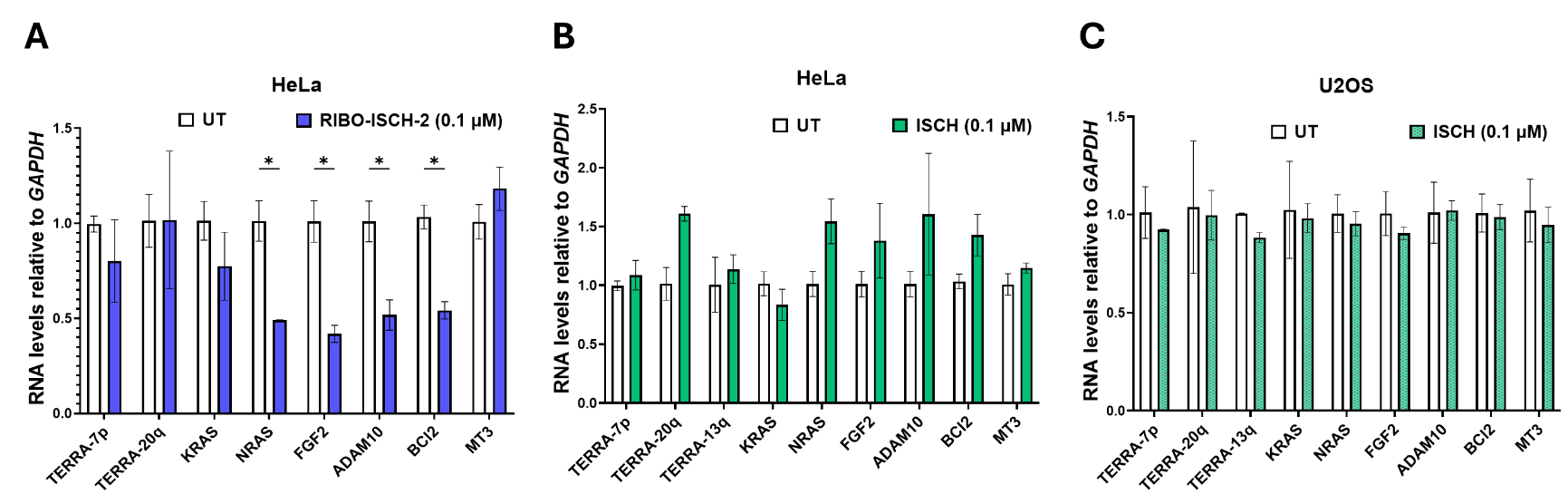
**

**Figure S17.** (A) Relative abundance of *RNase L* in HeLa cells treated with Mock, siRNase L, or scramble control, as determined by RT-qPCR. Data are normalized by the mean expression level in untreated control cells. Statistical comparisons were performed using unpaired t-test (*****p* < 0.0001). (B) Relative expression of RNase L in U2OS cells treated with Mock or siRNase L for 48 hours, as determined by western blot. Data are normalized to the housekeeping protein GAPDH and to untreated control cells. (C) Relative abundance of *TERRA-7p, TERRA-13q, TERRA-20q, BCL2, FGF2,* and *NRAS* in HeLa cells treated with Mock, siRNase L, or scramble control, as determined by RT-qPCR.

**
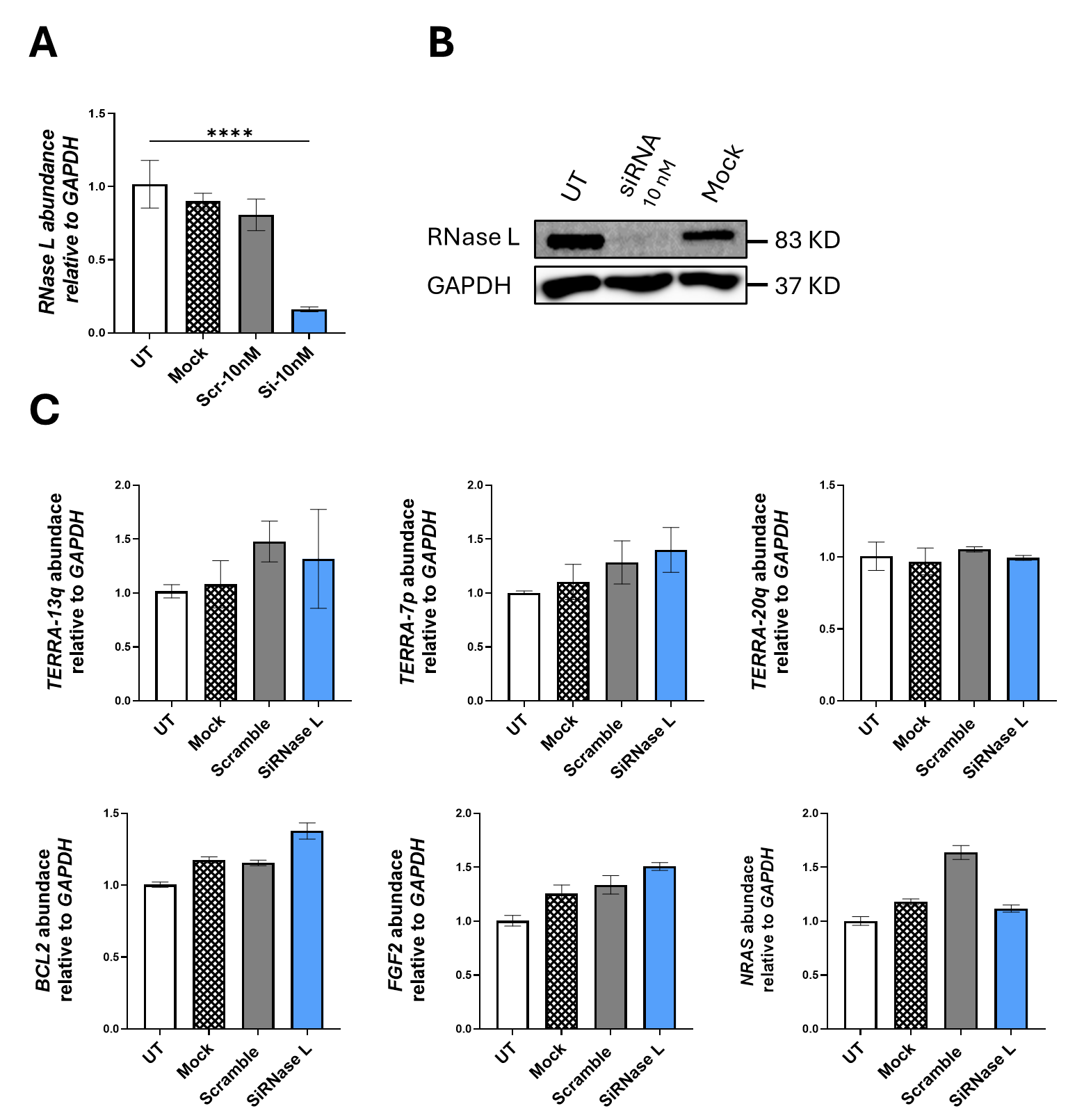
**

**Figure S18.** Relative abundance of *FGF2* following treatment with **RIBO-ISCH-2** (0.1 µM) for 48 hours in the presence or absence of siRNA targeting RNase L (10 nM) or non-targeting scramble control (10 nM), as determined by RT-qPCR. Data are normalized by the mean expression level in untreated control cells. Statistical comparisons were performed using unpaired t-test (**p* < 0.05).

**Figure S19.** (A) Schematic illustration of the nuclease sensitivity assay. Cells were treated with DNase I or RNase A and then with RIBO-ISCH-1. (B) Bar plot of emission change for RIBO-ISCH-1 (100 nM) alone, +DNase I (200 units/mL), or +RNase A (200 units/mL). Statistical comparisons were performed using unpaired t-test (**p* < 0.05). (λex = 550 nm, λem = 660 nm). Statistical comparisons were performed using unpaired t-test (**p* < 0.05).

**
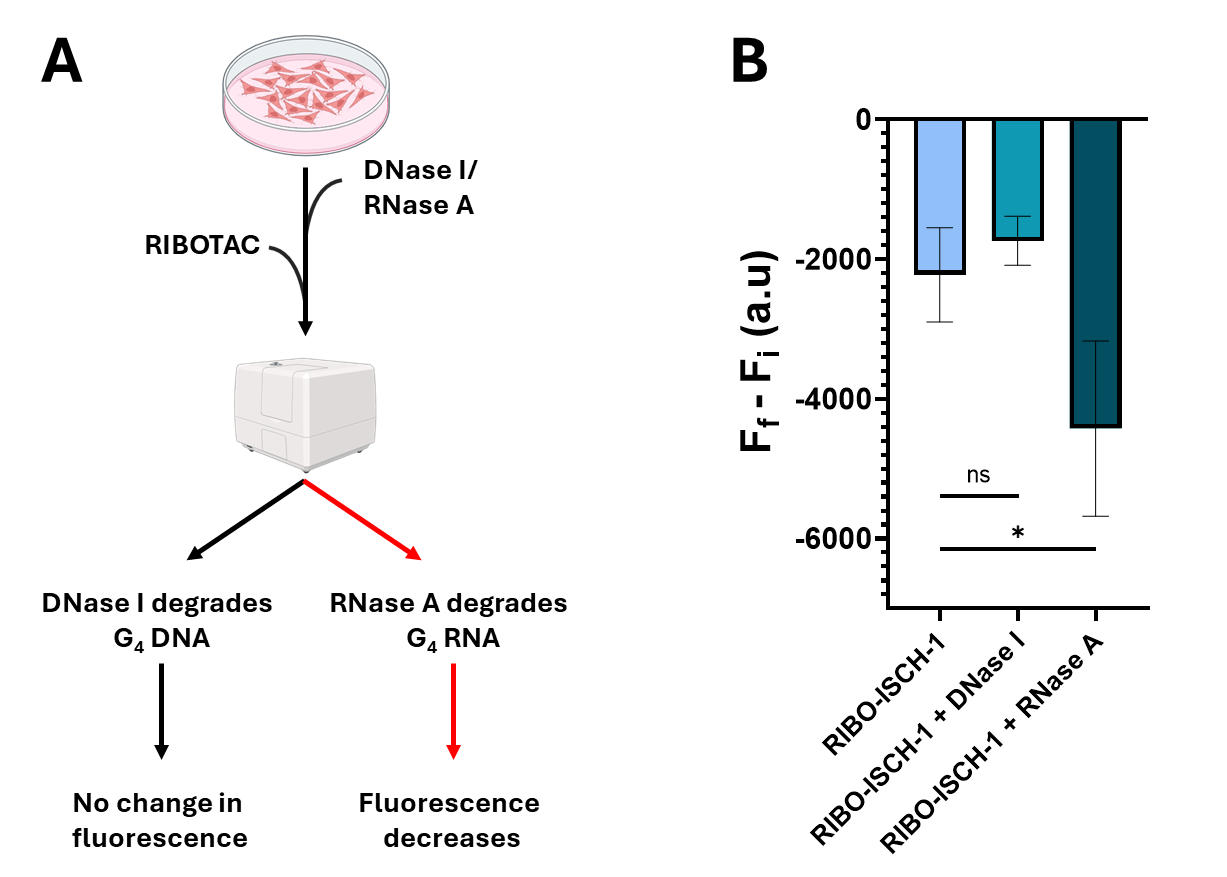
**

**Figure S20.** (A) Western blot analysis of RAD51 expression following treatment of U2OS cells with ISCH (100 nM), RIBO-ISCH-1 (100 nM), ASO (50 nM) or Scr (50 nM) for 48 hours. (B) Western blot analysis of FANCD2 expression following treatment of U2OS cells with ISCH (100 nM), RIBO-ISCH-1 (100 nM) ASO (50 nM) or Scr (50 nM) for 48 hours. The experiment was done as a duplicate and was performed twice.

**
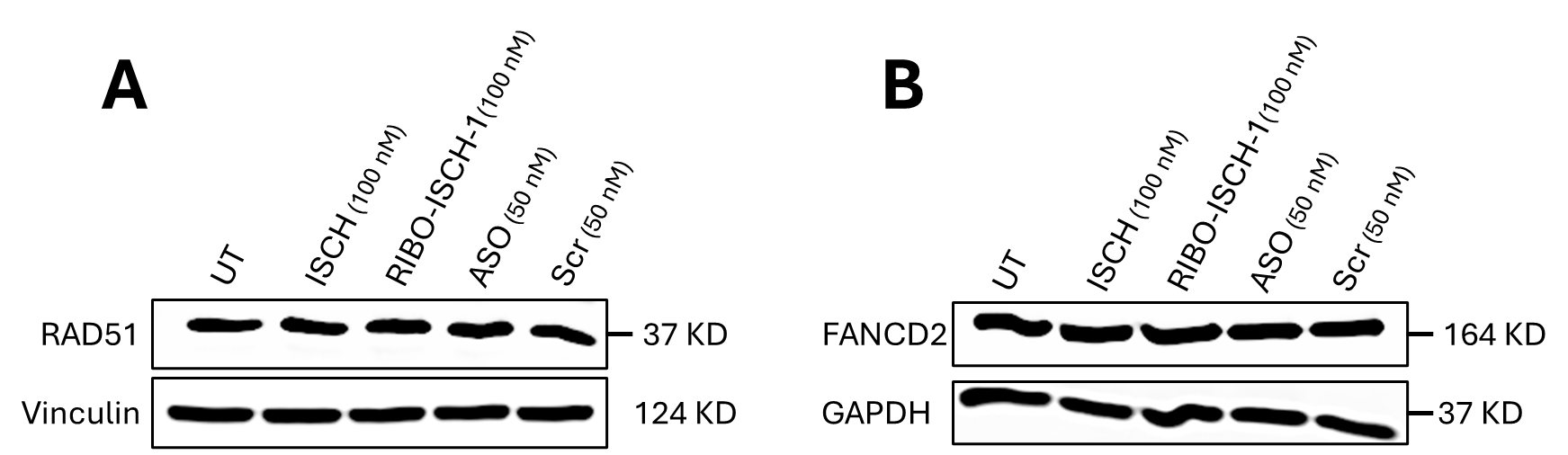
**

**
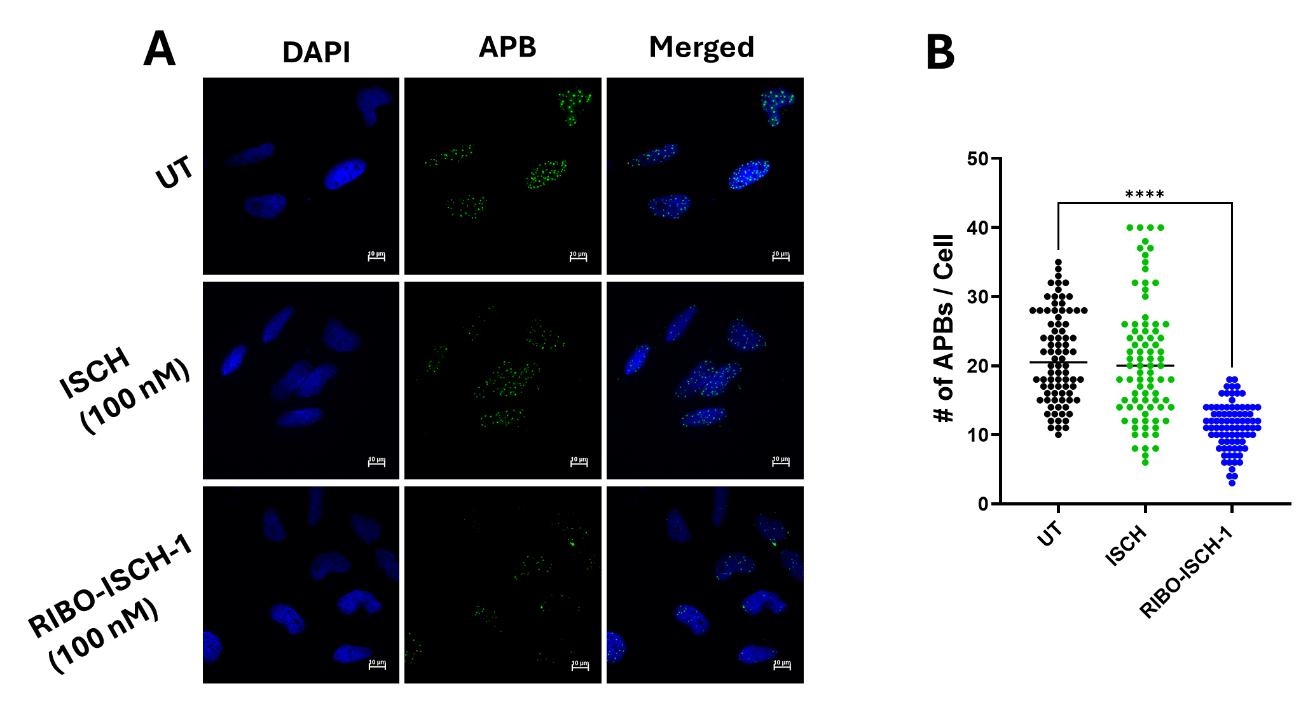
Figure S21.** (A) Immunofluorescence images of ALT-associated PML bodies (APBs) in HeLa cells stained with anti-PML antibody (green) and DAPI (blue) after treatment with ISCH or RIBO-ISCH-1 (0.1 µM) for 48 hours. (B) Quantification of APB number per nucleus in treated U2OS cells. (n=120 cells for all treatments over 3 different experiments), Statistical comparison was performed by two-tailed unpaired t-test (*****p* < 0.0001).

**Figure S22.** (A) Proliferation assay for U2OS cells treated with RIBO-IACH-1 for 48 hours, ASO (50 nM) for 12 hours, or Doxorubicin (3.5 µM) for 24 hours. (B) Western blot analysis of caspase-3 expression following treatment of U2OS cells with ISCH (100 nM), RIBO-ISCH-1 (100 nM), ASO (50 nM), Scramble (50 nM) or doxorubicin (3.5 µM) for 48 hours. Statistical comparison was performed by one-way ANOVA for RIBO-ISCH-1 (****p* < 0.001; *****p* < 0.0001) and by two-tailed unpaired t-test for ASO and doxorubicin (****p* < 0.001).

**
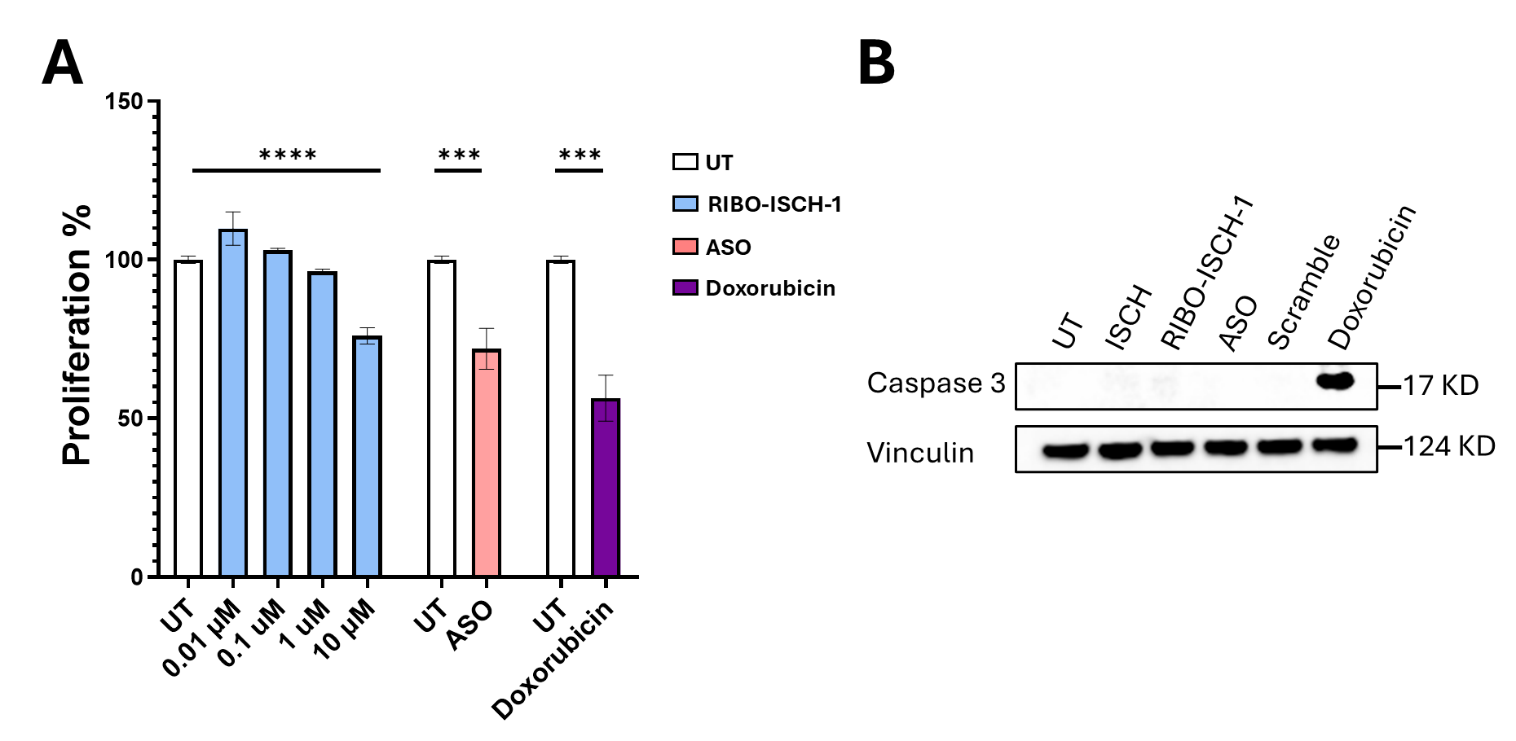
**

**Figure S23.** Analytical HPLC of G-Quadruplex binder (**ISCH**):


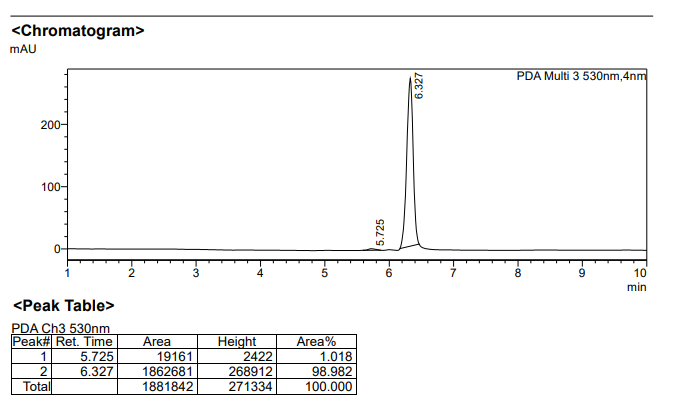


**Figure S24.** Analytical HPLC data for **RIBO-ISCH-1**:


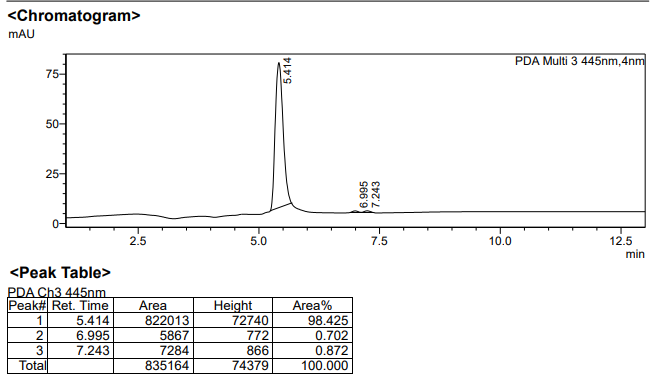


**Figure S25.** HRMS data for **RIBO-ISCH-1**:


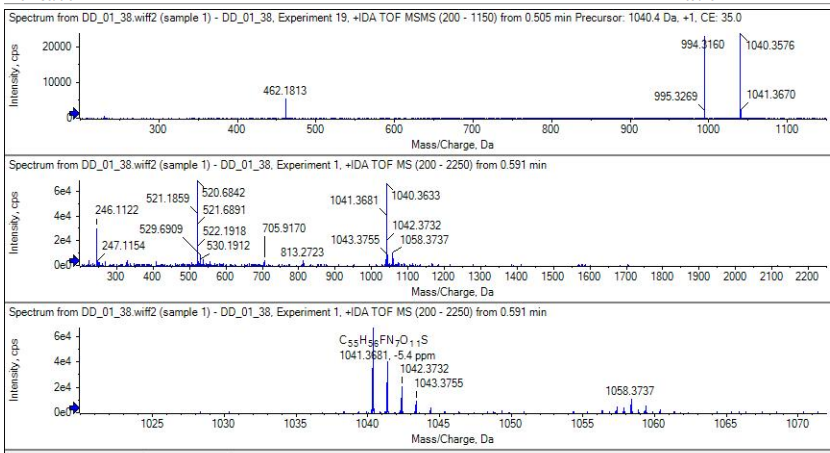


**Figure S26.** Analytical HPLC for **RIBO-ISCH-2**:


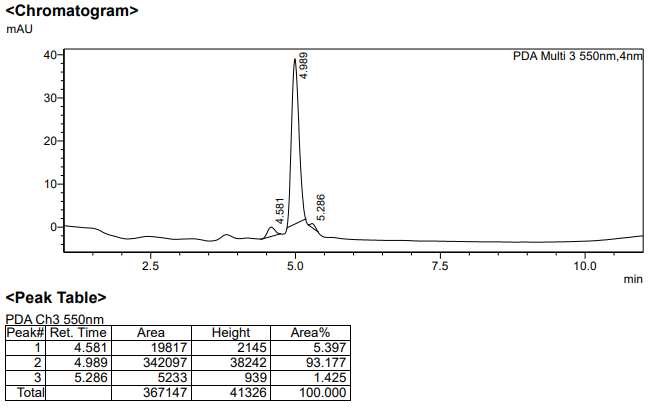


**Figure S27.** HRMS data for **RIBO-ISCH-2**:


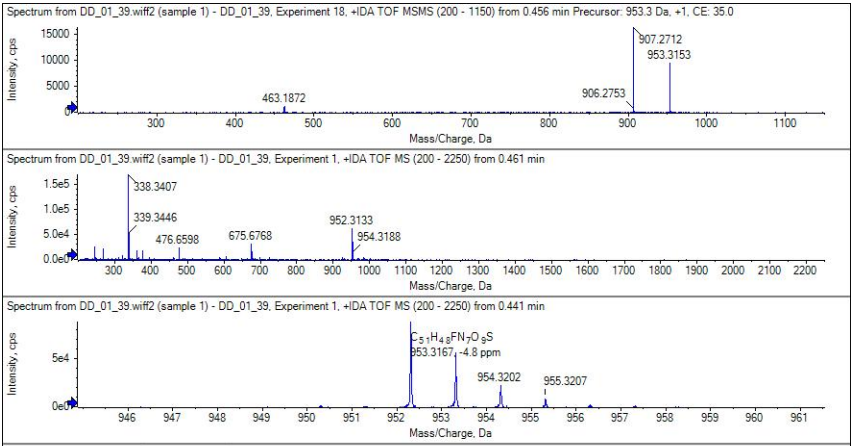


**Figure S28.** Analytical HPLC for **RIBO-ISCH-3**:


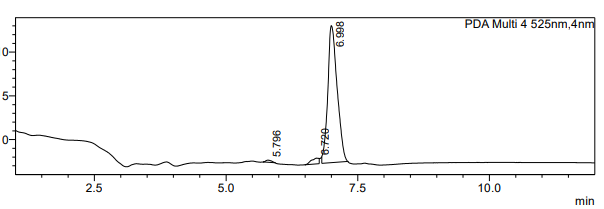


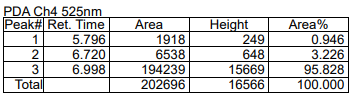


**Figure S29.** HRMS data for **RIBO-ISCH-3**:


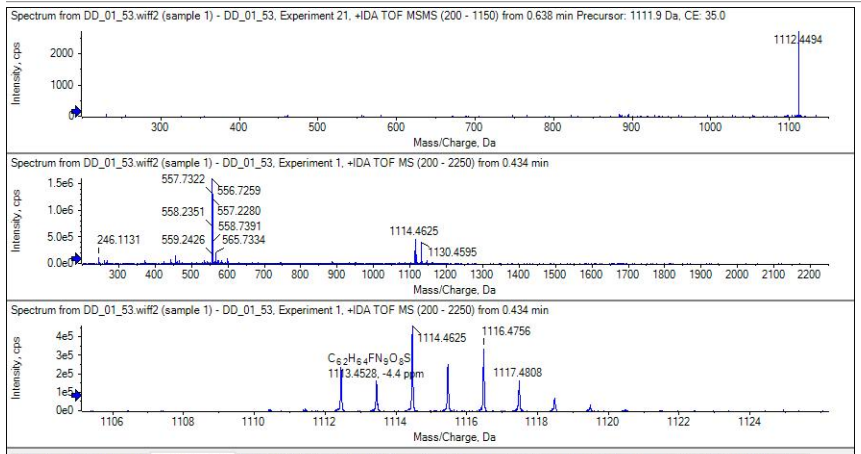


**Figure S30.** Analytical HPLC for **RIBO-ISCH-4**:


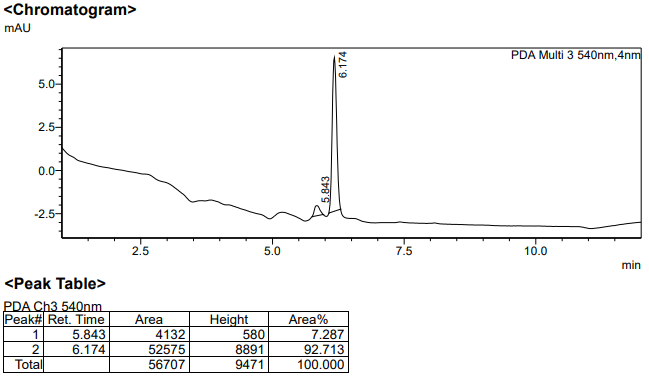


**
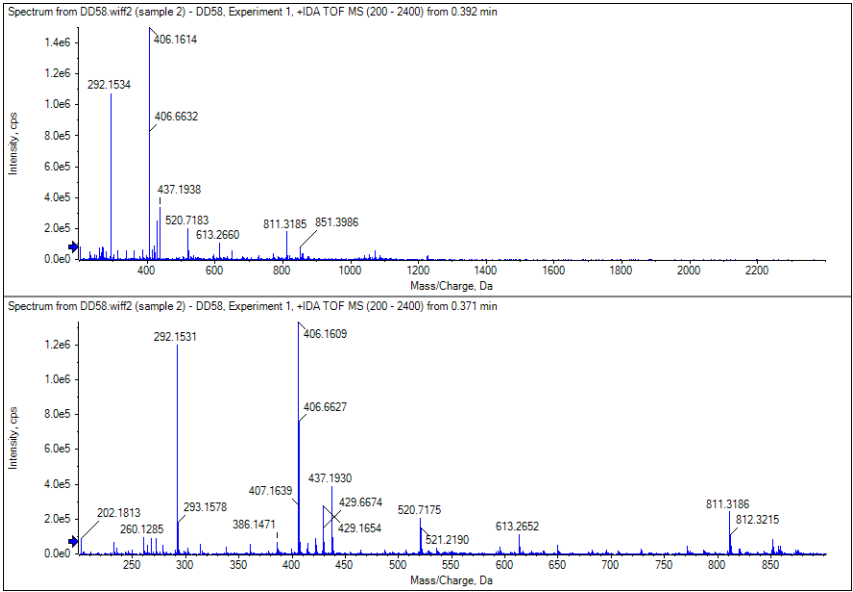
Figure S31.** HRMS for **RIBO-ISCH-4**

**Figure S32.** The ^1^H-NMR spectrum of ethyl-4-chloroacetoacetate. (**C-1**) in CDCl_3_. (300 MHz)

******Figure S33.** The ^1^H-NMR spectrum of 1-azido-2-(2-(2-bromoethoxy)ethoxy)ethane (**C-3**) in CDCl_3_. (300 MHz)

**Figure S34.** The ^1^H-NMR spectrum of 4-(2-(2-(2-azidoethoxy)ethoxy)ethoxy)-3-hydroxybenzaldehyde (**C-4**) in CDCl_3_. (300 MHz)

**
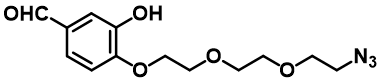

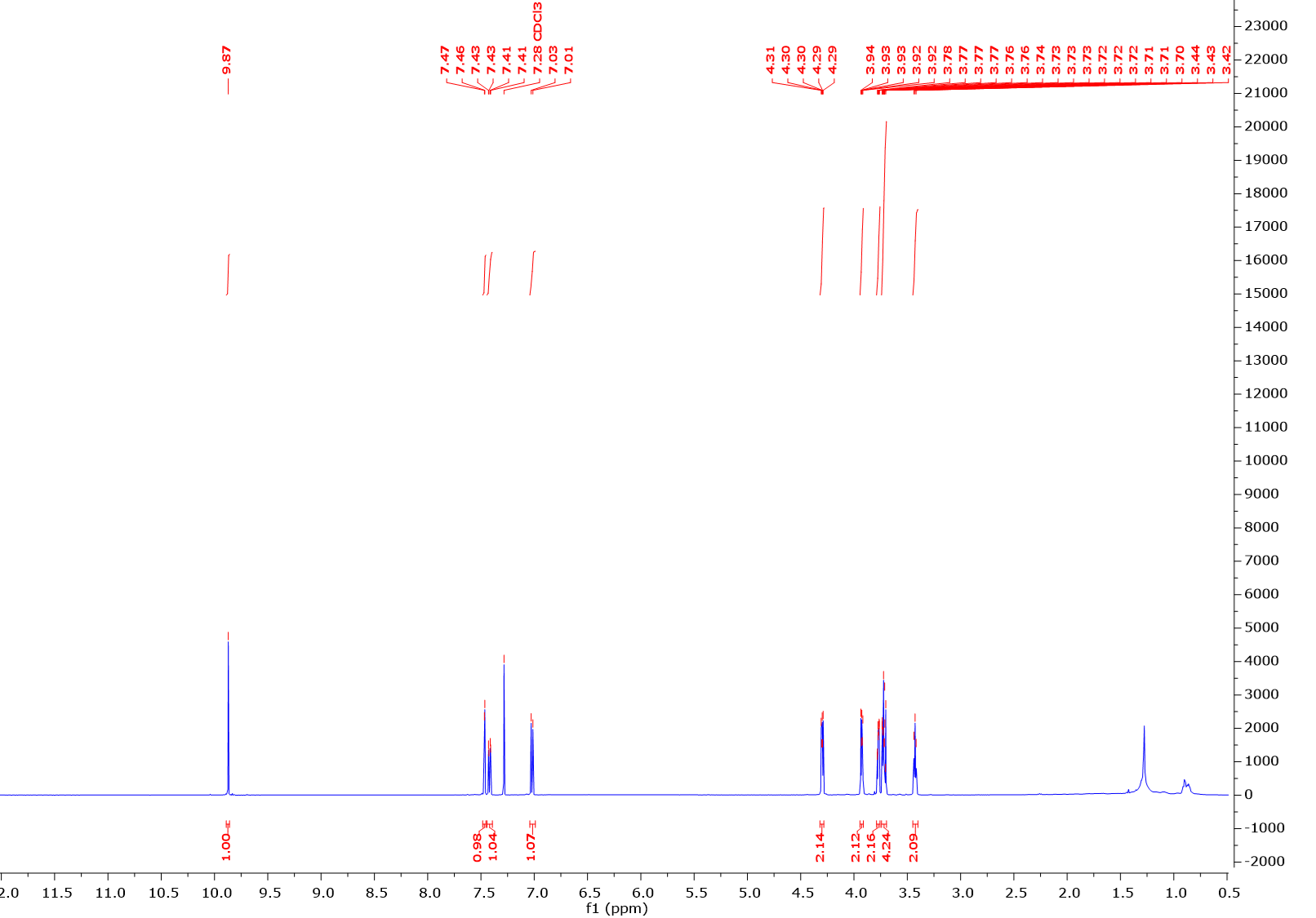
**

**
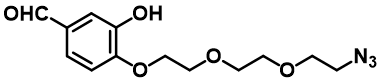
**
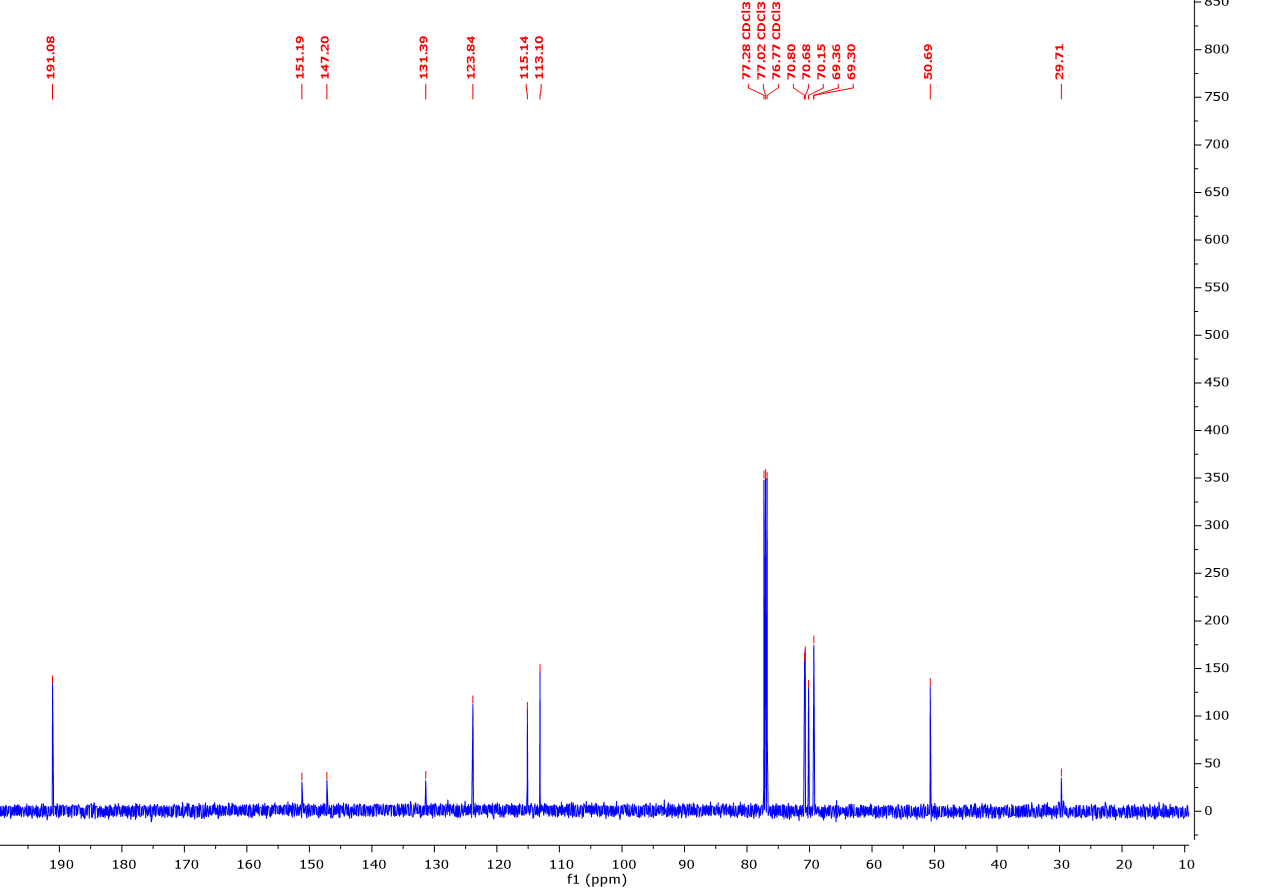
**Figure S35.** The ^13^C-NMR spectrum of 4-(2-(2-(2-azidoethoxy)ethoxy)ethoxy)-3-hydroxybenzaldehyde (**C-4**) in CDCl_3_. (300 MHz)

**Figure S36.** The ^1^H-NMR spectrum of ethyl (*Z*)-5-(4-(2-(2-(2-azidoethoxy)ethoxy)ethoxy)-3-hydroxybenzylidene)-4-oxo-2-(phenylamino)-4,5-dihydrothiophene-3-carboxylate (**Recruiter 1A**) in DMSO-*d_6_*. (300 MHz)

**Figure S37.** The ^1^H-NMR spectrum of 4-(2-azidoethoxy)-3-hydroxybenzaldehyde (**C-7**) in CDCl_3_. (500 MHz)


 **Figure S38.** The ^13^C-NMR spectrum of 4-(2-azidoethoxy)-3-hydroxybenzaldehyde (**C-7**) in CDCl_3_. (500 MHz)

**Figure S39.** The ^1^H-NMR spectrum of ethyl (*Z*)-5-(4-(2-azidoethoxy)-3-hydroxybenzylidene)-4-oxo-2-(phenylamino)-4,5-dihydrothiophene-3-carboxylate (**Recruier 1B**) in DMSO-*d_6_.* (300 MHz)


**
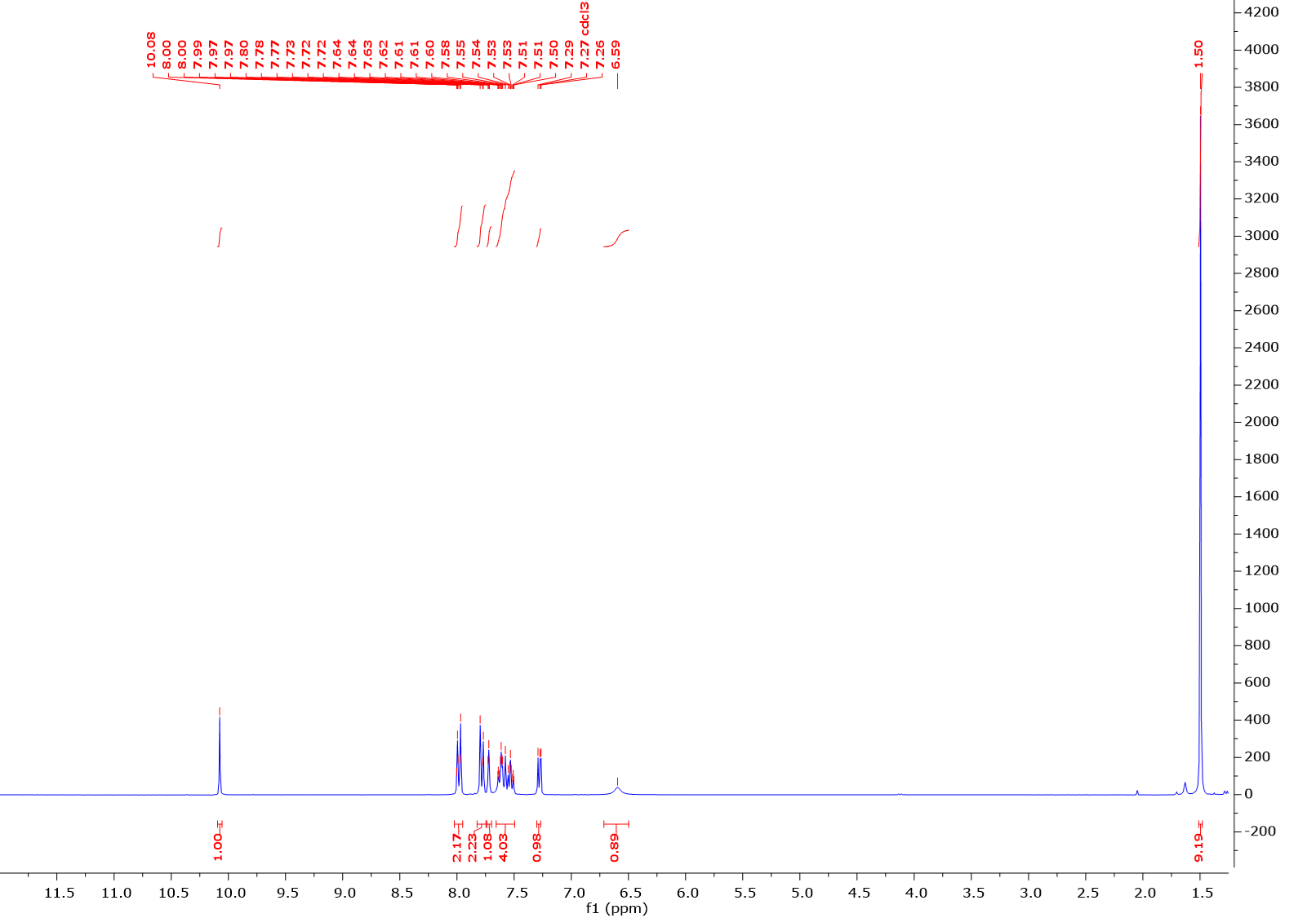
Figure S40.** The ^1^H-NMR spectrum of *Tert*-butyl (2-(4'-formyl-[1,1'-biphenyl]-3-yl)thiophen-3-yl)carbamate (**C-10**) in CDCl_3_. (300 MHz)

**Figure S41.** The ^1^H-NMR spectrum of (*S*)-2-(((3'-(3-Aminothiophen-2-yl)-[1,1'-biphenyl]-4-yl)methyl)(methyl)amino)-*N*-methyl-2-phenylacetamide (**C-13**) in CDCl_3_. (500 MHz)

**
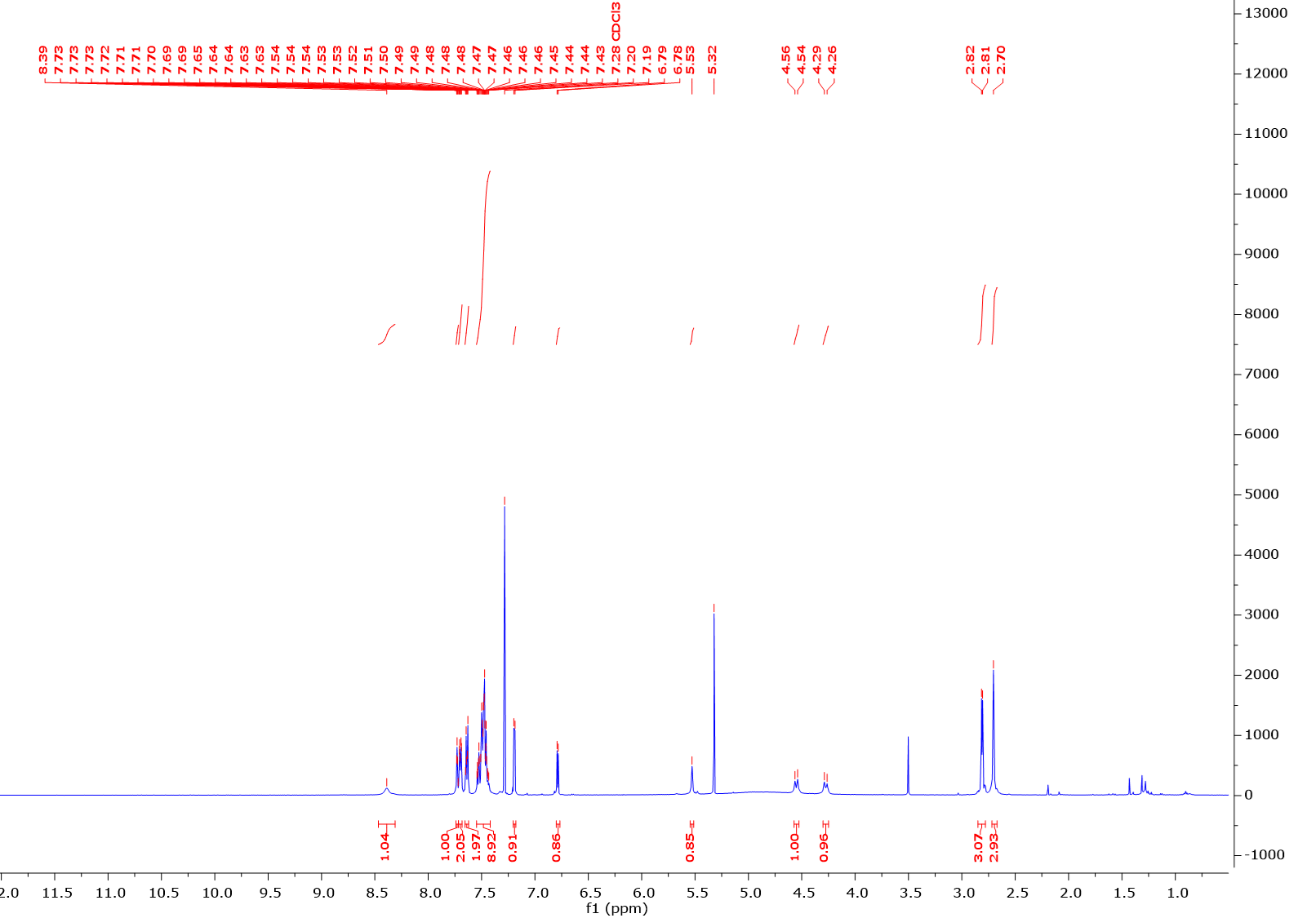
**

**Figure S42.** The ^1^H-NMR spectrum of **RIBO-ISCH-1** in CDCl_3_. (500 MHz)

**Figure S43.** The ^13^C-NMR spectrum of **RIBO-ISCH-1** in CDCl_3_. (500 MHz)

**6. References**

(1) Meyer, S. M.; Tanaka, T.; Zanon, P. R. A.; Baisden, J. T.; Abegg, D.; Yang, X.; Akahori, Y.; Alshakarchi, Z.; Cameron, M. D.; Adibekian, A.; Disney, M. D. DNA-Encoded Library Screening To Inform Design of a Ribonuclease Targeting Chimera (RiboTAC). *J Am Chem Soc* **2022**, *144* (46), 21096–21102. https://doi.org/10.1021/jacs.2c07217

(2) Costales, M. G.; Aikawa, H.; Li, Y.; Childs-Disney, J. L.; Abegg, D.; Hoch, D. G.; Velagapudi, S. P.; Nakai, Y.; Khan, T.; Wang, K. W.; Yildirim, I.; Adibekian, A.; Wang, E. T.; Disney, M. D. Small-Molecule Targeted Recruitment of a Nuclease to Cleave an Oncogenic RNA in a Mouse Model of Metastatic Cancer. *Proc Natl Acad Sci U S A* **2020**, *117* (5), 2406–2411. https://doi.org/10.1073/PNAS.1914286117

(3) Chen, S. Bin; Hu, M. H.; Liu, G. C.; Wang, J.; Ou, T. M.; Gu, L. Q.; Huang, Z. S.; Tan, J. H. Visualization of NRAS RNA G-Quadruplex Structures in Cells with an Engineered Fluorogenic Hybridization Probe. *J Am Chem Soc* **2016**, *138* (33), 10382–10385. https://doi.org/10.1021/jacs.6b04799

(4) Rahal, M.; Graff, B.; Toufaily, J.; Hamieh, T.; Noirbent, G.; Gigmes, D.; Dumur, F.; Lalevée, J. 3-Carboxylic Acid and Formyl-Derived Coumarins as Photoinitiators in Photo-Oxidation or Photo-Reduction Processes for Photopolymerization upon Visible Light: Photocomposite Synthesis and 3D Printing Applications. *Molecules 2021, Vol. 26, Page 1753* **2021**, *26* (6), 1753. https://doi.org/10.3390/molecules26061753
